# Two-Component Signaling System VgrRS Directly Senses Extracytoplasmic and Intracellular Iron to Control Bacterial Adaptation under Iron Depleted Stress

**DOI:** 10.1371/journal.ppat.1006133

**Published:** 2016-12-30

**Authors:** Li Wang, Yue Pan, Zhi-Hui Yuan, Huan Zhang, Bao-Yu Peng, Fang-Fang Wang, Wei Qian

**Affiliations:** 1 State Key Laboratory of Plant Genomics, Institute of Microbiology, Chinese Academy of Sciences, Beijing, China; 2 School of Biological Sciences, University of Chinese Academy of Sciences, Beijing, China; University of Toronto, CANADA

## Abstract

Both iron starvation and excess are detrimental to cellular life, especially for animal and plant pathogens since they always live in iron-limited environments produced by host immune responses. However, how organisms sense and respond to iron is incompletely understood. Herein, we reveal that in the phytopathogenic bacterium *Xanthomonas campestris* pv. *campestris*, VgrS (also named ColS) is a membrane-bound receptor histidine kinase that senses extracytoplasmic iron limitation in the periplasm, while its cognate response regulator, VgrR (ColR), detects intracellular iron excess. Under iron-depleted conditions, dissociation of Fe^3+^ from the periplasmic sensor region of VgrS activates the VgrS autophosphorylation and subsequent phosphotransfer to VgrR, an OmpR-family transcription factor that regulates bacterial responses to take up iron. VgrR-VgrS regulon and the consensus DNA binding motif of the transcription factor VgrR were dissected by comparative proteomic and ChIP-seq analyses, which revealed that in reacting to iron-depleted environments, VgrR directly or indirectly controls the expressions of hundreds of genes that are involved in various physiological cascades, especially those associated with iron-uptake. Among them, we demonstrated that the phosphorylated VgrR tightly represses the transcription of a special TonB-dependent receptor gene, *tdvA*. This regulation is a critical prerequisite for efficient iron uptake and bacterial virulence since activation of *tdvA* transcription is detrimental to these processes. When the intracellular iron accumulates, the VgrR-Fe^2+^ interaction dissociates not only the binding between VgrR and the *tdvA* promoter, but also the interaction between VgrR and VgrS. This relieves the repression in *tdvA* transcription to impede continuous iron uptake and avoids possible toxic effects of excessive iron accumulation. Our results revealed a signaling system that directly senses both extracytoplasmic and intracellular iron to modulate bacterial iron homeostasis.

## Author Summary

The biological function of iron is like a “double-edge sword” to all cellular life since iron starvation or iron excess leads to cell death. For animal and plant pathogens, they have to compete for iron with their hosts since iron-limitation generally is an immune response against microbial infection. However, how pathogens detect extracellular and intracellular iron concentrations remains unclear. Here we show that a plant bacterial pathogen employs a membrane-bound sensor histidine kinase, VgrS, to directly detect extracytoplasmic iron starvation and activate iron uptake accordingly. As a prerequisite, VgrS phosphorylates cognate VgrR to shut down the transcription of a downstream gene, *tdvA*, whose expression is harmful to absorb iron and bacterial virulence. However, as intracellular iron concentration increases, the ferrous iron binds to VgrR to release its repression on the *tdvA* transcription, which results in the block of continuous iron uptake to avoid toxic effect of the metal. Therefore, VgrS and VgrR detect extracytoplasmic and intracellular iron, respectively, and systematically modulate cellular homeostasis to promote bacterial survival in iron-depleted environments, such as in host plant.

## Introduction

As the fourth most abundant element in the Earth’s crust, iron is a metal essential for all cellular life. However, either iron limitiation or iron excess is harmful to organisms. The ferrous iron (Fe^2+^) acts as a cofactor that is required for essential physiological processes such as respiration, photosynthesis, energy metabolism and biosynthesis of multiple macromolecules [1]. But under aerobic conditions, excess iron can be extremely toxic to cells because it catalyzes the generation of highly reactive damaging hydroxyl radicals from superoxide and hydrogen peroxide [2]. Therefore, organisms must use effective mechanisms to detect both extracytoplasmic and intracellular iron concentrations and respond accordingly. When living under iron starvation conditions, iron is taken up into cells to maintain an appropriate level. Under excess iron conditions, iron should be exported outside cells or chelated to quench its toxic activity [3, 4]. However, how cellular organisms precisely balance the two opposite processes to achieve iron homeostasis remains unknown.

Pathogenic bacteria that invade host plants or animals live in an iron-limited environment where the hosts retain iron by complexing the metal with various proteins or small-molecular chemicals [3, 5]. Consequently, iron limitation is one of the innate immune responses of hosts to defend against microbial infection [2]. To obtain necessary iron, bacteria secrete various iron-carriers, named siderophores, to chelate environmental iron (usually ferric Fe^3+^), and take up the iron-containing siderophores into cells. The ferric iron is then reduced to the biological active ferrous iron (Fe^2+^) in the cytosol [6]. To achieve this, most bacteria employ the ferric uptake regulator (Fur) or the diphtheria toxin repressor (DtxR) to regulate iron-associated genes. When bound to Fe^2+^, Fur or DtxR homodimers generally bind the *cis*-regulatory elements of iron uptake-associated genes—such as those encoding outer membrane TonB-dependent receptors, the TonB-ExbB-ExbD transport complex or iron-storage proteins—to repress their transcription by preventing the binding of RNA polymerase [1, 7]. In iron-depleted conditions, transcriptional repression by Fur/DtxR is relieved to trigger iron uptake [4]. Bacterial two-component signal transduction systems (TCSs) are also involved in regulating iron metabolism. The TCS is the dominant sense-response molecular machinery in prokaryotes, usually comprising a sensor histidine kinase (HK) and a response regulator (RR). The HK monitors environmental stimuli and responds to them by autophosphorylation. HK then transfers the phosphoryl group to the cognate RR, and the activated RR modulates the expression of downstream genes or cellular behavior via its output domain [8]. In Gram-negative bacteria, the HKs PmrB and BqsS were observed to detect iron [9–11]. In Gram-positive bacteria, such as *Staphylococcus aureus* and *Bacillus subtilis*, the TCS HssR-HssS is employed to sense iron-containing heme and control heme resistance during infection [12, 13]. However, how other bacteria sense and respond to iron limitiation remains unclear.

The Gram-negative bacterium *Xanthomonas campestris* pathovar (pv.) *campestris* is a model organism to study plant-microbe interactions. It is the causative agent of black rot disease of cruciferous plants and causes serious yield loss in vegetable production worldwide. Genomic annotation revealed that this bacterium is equipped with a robust iron uptake system. It encodes a Fur protein, TonB-ExbB-ExbD transport system, and at least 72 TonB-dependent receptors, which is almost nine times that of *Escherichia coli* K12 [14]. Genome-wide mutagenesis experiments showed that inactivation of two genes encoding TonB-dependent receptors, *iroN* and *suxA*, significantly attenuated bacterial virulence [14, 15]. Similar to other pathogenic bacteria, mutation in the *fur* gene of *X*. *campestris* pv. *campestris* resulted in increased production of siderophores, reduced resistance to oxidative stress and decreased virulence [16]. Recently, a two-component signal transduction system, VgrR–VgrS (also named ColR-ColS), was identified as a canonical modulator controlling virulence of *X*. *campestris* pv. *campestris* [17, 18]. In *X*. *oryzae* pv. *oryzae*, a close relative of *X*. *campestris* pv. *campestris* and the causal agent of rice bacterial blight disease, this system controls the bacterial stress response to iron and the expression of two iron-uptake related genes, *feoB* and *xssE* [19]. These previous studies provided clues that suggested that VgrR–VgrS regulates iron metabolism during pathogenesis of *Xanthomonas* spp.

The present work analyzed the roles of VgrS–VgrR in regulating iron homeostasis during bacterial pathogenesis. We found that VgrS is a membrane-bound HK that detects extracytoplasmic iron starvation via its periplasmic sensor, whereas VgrR is a response regulator that detects intracellular iron excess. Under iron-depleted conditions, Fe^3+^ disassociates from the periplasmic sensor of VgrS and releases the inhibition of VgrS’s autophosphorylation, which triggers the uptake of extracellular iron. As one of the prerequisites of the process, the transcription of a special TonB-dependent receptor gene (*tdvA*, *XC1241*), whose expression is detrimental to iron uptake and bacterial virulence, must be tightly repressed by the phosphorylated VgrR. However, when iron accumulates to a high concentration within bacterial cells, Fe^2+^ directly binds to VgrR, causing dissociation between VgrR and the *cis*-regulatory element of *tdvA*, which leads to alleviation of *tdvA* repression. Thereafter, increased transcription of *tdvA* effectively inhibits the uptake of excessive iron from the environment. Our study revealed a simple but elegant biochemical system by which bacteria detect extracytoplasmic and intracellular iron concentrations, and regulate their pathogenesis accordingly.

## Results

### *vgrR*–*vgrS* is required for bacterial growth under both iron-replete and depleted conditions

**Fig 1:**
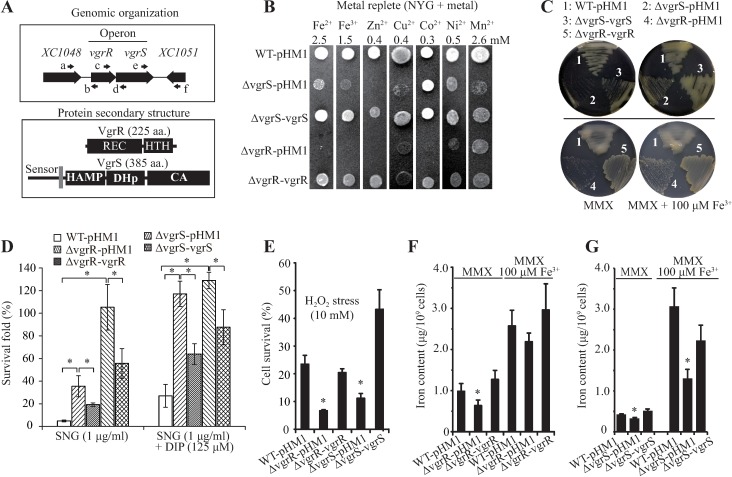
*vgrR-vgrS* modulates bacterial responses to iron-replete and depleted conditions. (A) Schematic view of the genomic organization of the *vgrR-vgrS* locus and the putative secondary structures of their protein products. Upper panel: large black arrows indicate genes and their transcription directions. Small arrows indicate the location of primers used for RT-PCR in Fig 2C. Lower panel: names of the protein domains are according to the pfam database. REC: receiver domain; HTH: helix-turn-helix domain; HAMP: domain presents in histidine kinases, adenylate cyclases, methyl accepting proteins and phosphatases; HisKA = DHp: dimerization and histidine phosphotransfer domain; CA = HATPase_C: catalytic domain of histidine kinase. A gray frame indicates the transmembrane region of VgrS. (B) Bacterial growth under different metal stresses. Bacterial strains were inoculated onto NYG plates containing different metals and incubated at 28°C for 48–72 h. Strain names are the same as in S1 Table. (C) Bacterial growth under iron-depleted condition. Strains were grown at 28°C on minimal MMX medium or MMX plus 100 μM FeCl_3_. Bacterial growth curves in liquid media are shown in S1C and S1D Fig. (D) Survival rates of bacterial strains subjected to streptonigrin treatment. Bacterial cultures (OD_600_ = 0.6) in NYG media were treated with streptonigrin (SNG) for 16 h and the survival rates were calculated by counting the cell numbers before and after the stress challenge. 2, 2′-dipyridyl (DIP) (125 μM) was added into NYG medium to chelate free iron ions. Vertical bars represent standard deviations (n = 3). (E) Survival rate of bacterial strains subjected to H_2_O_2_ stress. Bacterial cultures (OD_600_ = 0.6) in NYG media were treated with H_2_O_2_ (10 mM) for 15 minutes and survival rates were calculated by counting cell numbers before and after the stress challenge. Vertical bars represent the standard deviations (n = 3). (F and G) Concentration of total iron in bacterial cells. Equal volumes of bacterial cells were cultured in minimal medium MMX or MMX plus 100 μM Fe^3+^. The amounts of total iron in the cells were measured using inductively coupled plasma spectroscopy (ICP-OES). Vertical bars represent the standard deviations (n = 3). In (D, E, F and G), * indicate significant differences (Student’s *t*-test, P < 0.05) between samples and the wild-type (WT) strain under the same test conditions or as indicated.

VgrS is an orthodox HK, containing a dimerization and histidine phosphotransfer (DHp) domain and a catalytic histidine kinase (CA) domain. VgrR is an OmpR-family transcription factor (TF) that has an N-terminal receiver domain and a C-terminal DNA binding domain (Fig 1A). To investigate whether *vgrR*–*vgrS* of *X*. *campestris* pv. *campestris* is involved in the bacterial response to metal stress, an in-frame deletion mutant of *vgrS* (Δ*vgrS*) was constructed. Repeated efforts to construct a *vgrR* in-frame deletion mutant have failed. This might be caused by the fact that *vgrR* mutation suppresses recombination since no correct clone of the second homologous cross-over was obtained. Thereafter, a marker-exchange mutant (Δ*vgrR*) was constructed successfully by substituting the gene with a sequence encoding a tetracycline resistance cassette. Plant inoculation confirmed that the mutants of *vgrR* and *vgrS* were attenuated in virulence against host plant *Brassica oleraceae* cv. Jingfeng No. 1, and genetic complementation restored the deficiency towards that of the wild-type strain (S1A and S1B Fig). Under the optimized metal stress conditions of Fe^2+^ (2.5 mM), Fe^3+^ (1.5 mM), Zn^2+^ (0.4 mM), or Cu^2+^ (0.4 mM), the *vgrR* and *vgrS* mutants exhibited remarkably slower growth compared with those of the wild-type (WT) strain, and genetic complementation completely or partially suppressed the growth deficiencies (Fig 1B). High concentrations of Co^2+^ (0.3 mM), Ni^2+^ (0.5 mM), and Mn^2+^ (2.6 mM) also showed weaker growth inhibitory effects on the *vgrR* and *vgrS* mutants (Fig 1B). Pathogenic bacteria grow under iron-depleted conditions during pathogenesis; therefore, we also measured bacterial growth under the iron-depleted conditions. As shown in Fig 1C and S1C Fig and S1D Fig, addition of Fe^3+^ into minimal MMX medium substantially improved the growth of the *vgrR* or *vgrS* mutants. These results suggested that *vgrR–vgrS* are required for bacterial growth in both iron-replete and iron-depleted environments.

To further characterize the relationship between iron supply and *vgrR–vgrS* regulation, bacterial resistance to antibiotic streptonigrin (SNG) was measured. SNG is a chemical that requires iron for its bactericidal action and high levels of intracellular iron enhances its toxic effect [20, 21]. As shown in Fig 1D, under both iron-replete (rich NYG medium) and iron-depleted conditions (NYG plus 125 μM excessive iron-specific, membrane-permeable chelator 2,2-dipyridyl, DIP), the survival rate of *vgrR* or *vgrS* mutants in the presence of SNG was significantly higher than that of the WT strain (by 4.3–21.6 fold), and the genetic complementation decreased the survival capability of the mutants towards that of the WT strain. Bacterial cells exhibiting hypersensitivity to SNG usually have reduced superoxide dismutase (SOD) activity [22], treatment of these strains by H_2_O_2_ stress (10 mM for 15 min) reduced the survival rates of the *vgrR* and *vgrS* mutants by 0.29–0.48 fold compared with that of the WT strain. Genetic complementation completely restored or even increased the survival rate compared with the WT strain (Fig 1E). Collectively, these results suggest that the intracellular concentrations of iron are decreased in *vgrR* and *vgrS* mutants, which made them relatively insensitive to SNG treatment but more susceptible to superoxide stress.

Based on these results, the intracellular concentration of total iron in the *vgrR* and *vgrS* mutants were quantified using inductively coupled plasma optical emission spectrometry (ICP-OES). After growth for 3 h in the iron-depleted MMX medium, the iron accumulation in cells of *vgrR* and *vgrS* mutants significantly decreased to 64.7% and 71.5% of the levels in the WT strain, respectively, and genetic complementation completely restored this deficiency (Fig 1F and 1G). In the iron-replete medium (MMX + 100 μM FeCl_3_), the iron concentration in the *vgrR* and *vgrS* mutants also decreased to 85.1% and 42.5% of the levels in the WT strain (Fig 1F and 1G). Under iron-replete condition, mutation in *vgrS* resulted in a more decrease of iron content than that of the *vgrR* mutant. Taken together, these results supported the view that *vgrR*–*vgrS* regulates iron homeostasis in *X*. *campestris* pv. *campestris*, especially under iron-depleted conditions.

### VgrR–VgrS is a *bona-fide* two-component signaling system

**Fig 2:**
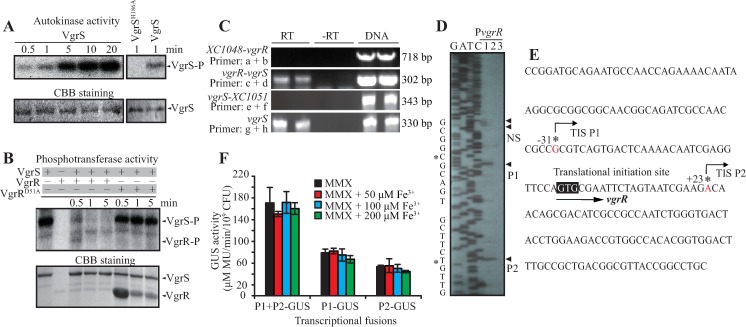
VgrR-VgrS is a two-component signaling system. (A) VgrS has autokinase activity. Inverted membrane vesicles of full-length VgrS and VgrS^H186A^ recombinant proteins (5 μM) were incubated with 100 μM ATP containing 10 μCi [γ-^32^P]ATP. (B) VgrS transferred phosphoryl group to VgrR. VgrS (5 μM) was autophosphorylated as in (A) for 2 min. Twenty μM VgrR or 80 μM VgrR^D51A^ proteins were added into the reaction, respectively. In both (A) and (B), the reaction was stopped by loading buffer before SDS-PAGE separation and autoradiography. The gel was stained by Coomassie brilliant blue (CBB) to check the amount of proteins (lower panels). Each experiment was repeated three times. (C) *vgrR* and *vgrS* constitute a bicistronic operon. RT-PCR was used to amplify cDNA. RT: amplification using cDNA transcribed from total RNA as template.–RT: negative control lacking reverse transcriptase during cDNA synthesis. DNA: amplification using bacterial DNA as template. Amplification of *vgrS* cDNA was used as a positive control. Location of the primers is shown in Fig 1A (upper panel). The assay was repeated three times. (D) Mapping the transcription initiation site (TIS) of the *vgrR-vgrS* operon. Primer extension using total RNA of *X*. *campestris* pv. *campestris* as the template. The G-A-T-C lanes show the dideoxy chain termination sequencing reaction in the same promoter region (note that the top of the ladder is blur for high GC content of the template). 1 and 2: *vgrR* mRNA was reverse transcribed at 42°C and 52°C, respectively, 3: △vgrR mRNA template (with primer binding site being deleted) was reverse transcribed at 42°C as negative control. TIS sites (P1 and P2) are shown as asterisks. NS: non-specific bands. The experiment was repeated independently twice. (E) Nucleotide sequence of the 5′ region upstream of *vgrR*. (F) GUS activity assay of promoters. GUS activity assays were conducted among all the recombinant bacterial strains with transcriptional fusions of P1-GUS, P2-GUS and P1+P2-GUS, respectively. Bacterial strains were induced in iron depleted (MMX) or iron replete (MMX plus Fe^3+^) conditions. Vertical bars represent the standard deviations (n = 3).

To biochemically verify that VgrR–VgrS is a TCS, full-length VgrR and VgrS were expressed and purified. *In vitro* phosphorylation assays revealed that VgrS has an autokinase activity by phosphorylating itself in the presence of ATP, while substitution of the conserved phosphorylation site (His residue, VgrS^H186A^) completely abolished the autokinase activity (Fig 2A). Addition of 20 μM of recombinant VgrR into the reaction mixture resulted in a substantial decrease of the level of phosphorylated VgrS (VgrS-P), and a band representing phosphorylated VgrR (VgrR-P) was observed at the 0.5 min after VgrR addition (Fig 2B). If a recombinant VgrR (VgrR^D51A^) with a substituted residue in its conserved phosphorylation site (Asp^51^) was added, the VgrS-P level was unaffected and no VgrR-P signal appeared (Fig 2B). In addition, the soluble, truncated VgrS containing linker-DHp-CA regions fused with an N-terminal maltose-binding protein (MBP) tag also has autokinase and phosphotransferase activities as the full-length form (S2 Fig). These biochemical results demonstrated that VgrS is a HK and transfers a phosphoryl group onto the conserved Asp residue of VgrR; therefore, they constitute a *bona fide* TCS. In addition, the half-life of VgrR-P is likely shorter than 0.5–1.0 min under the reaction conditions used.

Currently, the operon organization of *vgrRS* locus and biochemical nature of this putative TCS remains unclear. Reverse transcription PCR (RT-PCR) analysis was used to dissect the structure of the *vgrRS* operon. As shown in Fig 2C, RT-PCR amplification obtained a product that corresponded to the transcript from intergenic region between *vgrR* (*XC1049*) and *vgrS* (*XC1050*), but did not amplify a product from the intergenic regions between *XC1048* and *vgrR*, or between *vgrS* and *XC1051*. This result supported the view that *vgrR* and *vgrS* form a bicistronic operon and are under the control of the same *cis*-regulatory elements.

To map the transcription initiation site (TIS) of the *vgrR*–*vgrS* operon, primer extension analysis was conducted using total RNA of *X*. *campestris* pv. *campestris* as the template. Two unambiguous, reproducible bands were obtained, indicating two TISs at an upstream guanosine at −31 (P1) and a downstream adenosine at +23 (P2) relative to the translational initiation codon (GTG, Fig 2D and 2E). We assumed that the *vgrRS* operon is transcribed from the −31 site, while the biological function of the +23 intragenic TIS is unknown. To verify the promoters corresponding to the P1 and P2 TISs and measure their activity under low iron, we constructed three recombinant pHM2 vectors, each containing a DNA insert in which P1, P2, or the DNA region containing P1 and P2 was transcriptionally fused to the coding sequences of a *GUS* reporter gene. The three recombinant vectors were transformed into WT cells of *X*. *campestris* pv. *campestris* separately. As shown in Fig 2F, all three constructs produced GUS activity. In addition, neither the iron-replete nor the iron-depleted growth conditions affected the activities of the GUS reporters, suggesting that the transcription of the *vgrR*–*vgrS* operon itself did not respond to different iron concentrations. Therefore, similar to other TCSs, VgrR–VgrS’s regulation in response to iron probably occurs by phosphorylation-dependent control by the TF VgrR.

### Comparative proteomic analysis revealed that VgrR represses *XC1241* transcription in iron-depleted condition and *in planta*

**Fig 3:**
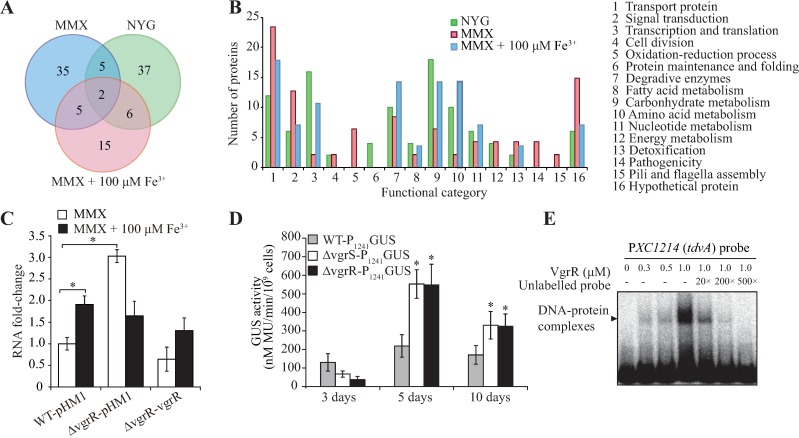
Comparative proteomics revealed that VgrR is the repressor of *XC1241* transcription. (A) Venn diagram showing the numbers of differently abundant proteins identified by a comparative proteomic approach. Total proteins of the wild-type (WT) strain and *vgrR* mutant grown in NYG, MMX, and MMX plus Fe^3+^ (100 μM) media, respectively, were analyzed by 2-DE together with mass spectrometry identification. The experiment was repeated independently three times. (B) Functional categories of differentially abundant proteins. The complete protein list is shown in S3–S5 Tables. (C) *vgrR* repressed *XC1241* transcription under iron-depleted conditions. qRT-PCR assays were used to quantify the amount of mRNA in the bacterial strains. WT-pHM1: wild-type strain containing a blank pHM1 vector. Δ*vgrR*-pHM1: *vgrR* mutant containing a blank pHM1 vector. Δ*vgrR*-*vgrR*: genetic complementation strain. A representative of three independent experiments is shown. (D) GUS activity assay of the *XC1241* promoter in bacteria grown *in planta*. Bacterial strains containing promoter-GUS fusions were inoculated into host plants before quantification of GUS activity. In both (C) and (D), vertical bars indicate the standard deviation (n = 3). * indicates significantly difference compared to those of the WT strain (Student’s *t*-test, P < 0.05). (E) Electrophoretic mobility shift assay showing that VgrR binds directly to the *XC1241* promoter region. Each lane contains 4 fmol of DNA probe labeled by [γ-^32^P]ATP. Increasing amounts of unlabeled probes (20–500 times) were used as competitors. The assay was repeated three times.

The VgrR–VgrS regulon has not been investigated, and thus a comparative proteomic method was employed to identify differentially abundant proteins under various growth conditions. Overnight cultured bacterial cells of the WT strain and the *vgrR* mutant were collected, washed, and then grown for 3 h in the rich NYG (iron-replete), minimal MMX (iron-depleted), and MMX plus iron (100 μM FeCl_3_) media, respectively. Bacterial total proteins were extracted, separated by two-dimensional electrophoresis (2DE), and the differentially abundant proteins were identified by matrix-assisted laser desorption ionization–time of flight mass spectrometry (MALDI-TOF-MS/MS). The numbers of differentially abundant proteins (change > 1.5 fold) between the WT strain and *vgrR* mutant were 50, 47, and 28 in NYG, MMX, and MMX plus iron media, respectively (Fig 3A and S3–S5 Tables). In total, the comparative proteomic analysis revealed that VgrR directly or indirectly controls the expression of 105 proteins involved in transportation, signal transduction, detoxification, cell division, biosynthesis of macromolecules and cellular metabolism (Fig 3B and S3–S5 Tables).

Notably, under iron-depleted condition (MMX), the amounts of four TonB-dependent receptors (TBDRs) (XC0806, XC2194, XC1644 and XC4053) were decreased in the *vgrR* mutant, whereas the TBDRs (XC1122, XC1241 and XC1619) were increased (S4 Table). The number of differentially abundant TBDRs was remarkably higher than that in NYG medium (two TBDRs) or in iron-replete conditions (two TBDRs), suggesting the VgrR modulates the bacterial iron uptake process. The induction of TBDR gene expression by transcription factors during bacterial infection has been studied extensively because the products are responsible for iron uptake [6]. However, the biological significance of repression of TBDR genes when iron is limited remains unclear. Among the three upregulated TBDR genes, semi-quantitative RT-PCR showed that the transcription of *XC1122* and *XC1619* was stable compared with the WT strain, suggesting that changes in their protein levels were not caused by altered transcription (S3 Fig).

For *XC1241*, a quantitative real-time reverse transcription PCR (qRT-PCR) assay revealed that in the WT strain, its mRNA level was significantly decreased to 52.3% level when bacteria were grown under the iron-depleted condition (Fig 3C), suggesting that its expression is repressed in this circumstance. However, for the *vgrR* mutant, the repression of *XC1241* transcription was completely released: *XC1241* mRNA levels in bacteria grown under iron-depleted conditions were significantly higher than those grown under iron-replete conditions (by 184.0%). Genetic complementation of *vgrR* restored the pattern of *XC1241* transcription to that of the WT strain (Fig 3C). This result agreed with that of the comparative proteomic analysis. Further, to measure the *XC1241* transcription when bacteria were grown *in planta*, we constructed a recombinant pHM2 vector with a DNA insert containing a transcriptional fusion of the promoter region of *XC1241* and a GUS reporter coding sequence. The vector was transformed into the WT strain, the *vgrR* and *vgrS* mutants, separately. The bacterial strains were cultured, inoculated into host plants, and the activity of the GUS reporter was measured. As shown in Fig 3D, after inoculation into host tissues, the GUS activity from the *vgrR* or *vgrS* mutants was significantly higher than that of the WT strain. For example, at 5 days after inoculation, the GUS activities of the *vgrR* and *vgrS* mutants were 2.53 and 2.51 times higher than those of the WT strain, suggesting that the VgrR–VgrS system also represses the transcription of *XC1241* when the bacteria are grown *in planta*.

To detect whether VgrR regulates *XC1241* transcription directly, a 188 bp PCR product of the 5′ sequence upstream of the *XC1241* ORF was labeled by [γ-^32^P]ATP and used as DNA probe in an electrophoretic mobility shift assay (EMSA). The result showed that the VgrR protein binds to this double-stranded DNA probe. Competition by an unlabeled DNA probe completely eliminated the isotopic signal of the VgrR–DNA complex, supporting the view that the protein–DNA interaction is specific (Fig 3E). Collectively, the results demonstrated that VgrR binds to the promoter region of *XC1241* directly. In addition, the TF represses *XC1241* transcription under iron-depleted conditions and in host plants. In iron-replete conditions, it is likely that VgrR does not repress the transcription of *XC1241* because the *vgrR* and *vgrS* mutations did not lead to significant change in the *XC1241* transcription level.

### Identification and verification of the VgrR binding DNA motif

**Fig 4:**
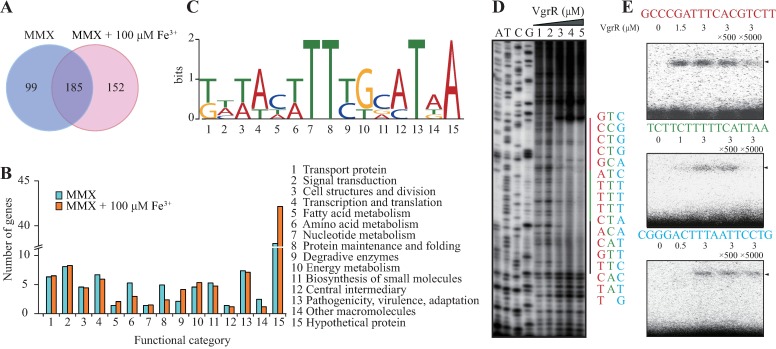
Dissection of the VgrR binding consensus motif. (A) Venn diagram showing the number of VgrR-regulated genes identified by ChIP-seq. (B) Functional categories of the VgrR-regulated genes identified by ChIP-seq. Details of the genes are listed in S6 and S7 Tables. (C) Deduced consensus VgrR-binding DNA motif based on ChIP-seq data. Weblogo was used to show the nucleotide composition. (D) Mapping the VgrR protected DNA region in the 5′ upstream sequence of *XC1241* (*tdvA*) by DNase I footprinting. The amounts of VgrR protein used in the reactions were 1: zero; 2: 0.08 μM; 3: 0.8 μM; 4: 3.2 μM; and 5: 8.0 μM. The DNA regions protected by VgrR are shown on the right of the footprinting results, with the three possible VgrR-binding motifs shown in red, green, and black, respectively. A-T-C-G lanes are the DNA ladders obtained by a dideoxy-mediated chain-termination method using the same DNA sequence as the template. (E) Electrophoretic mobility shift assay verified the DNA motif of the *XC1241* promoter bound by VgrR. The DNA probes were chemically synthesized according to those shown in (D). Sequences of the promoter region of *XC1241* are listed above each panel. Each DNA probe was labeled by [γ-^32^P]ATP. Triangles indicate the VgrR-DNA complexes. All experiments were repeated three times.

To identify the consensus VgrR binding motif, chromatin immunoprecipitation together with high throughput sequencing (ChIP-seq) was used to screen for VgrR-binding DNA sequences on a genome-wide scale. A recombinant strain that contained a pHM1::*vgrR*-his_6_ vector under the Δ*vgrR* background was constructed. The strain was cultured in iron-depleted and iron-replete conditions, and a monoclonal anti-His_6_ antibody was used to retrieve the VgrR-His_6_ protein bound to its DNA. After high throughput sequencing, peak calling revealed that VgrR binds to 464 and 655 genomic sites under iron-depleted and iron-replete conditions, respectively. Among them, the TF binds to the putative promoter regions of 284 and 337 genes in iron-depleted and iron-replete conditions, respectively, with 185 of them being shared (Fig 4A). Functional classification of these genes is summarized briefly in Fig 4B (the details are listed in S6 and S7 Tables). It is likely that the transcription of these 436 genes is regulated directly by VgrR. Together with the results from the comparative proteomic analysis, these genes represent VgrR regulon under the tested conditions.

Based on the ChIP-seq results, a consensus VgrR binding DNA motif was predicted from the putative promoter regions of the VgrR-regulated genes (Fig 4C). To verify the motif experimentally, the promoter region of *XC1241* was selected and DNase I footprinting was used to map the VgrR protected double-stranded DNA region. As shown in Fig 4D, VgrR protected a 50 bp DNA region in the 5′ upstream sequence of *XC1241*. EMSA also confirmed the binding between VgrR and this DNA region (S4 Fig). In this region, three degenerate DNA motifs similar to the predicted motif were found (Fig 4D). EMSA using synthetic DNA sequences corresponding to these motifs as probes demonstrated that VgrR specifically binds to two of them (Fig 4E, the upper two panels), while the third is non-specific as the unlabeled DNA probe failed to compete with the labeled probe (Fig 4E, the lowest panel). Taken together, these results revealed the consensus binding motif of VgrR in the genome of *X*. *campestris* pv. *campestris*. The promoter region of *XC1241* contains two such *cis*-regulatory elements.

### *XC1241* (*tdvA*) expression is detrimental to iron uptake and virulence

**Fig 5:**
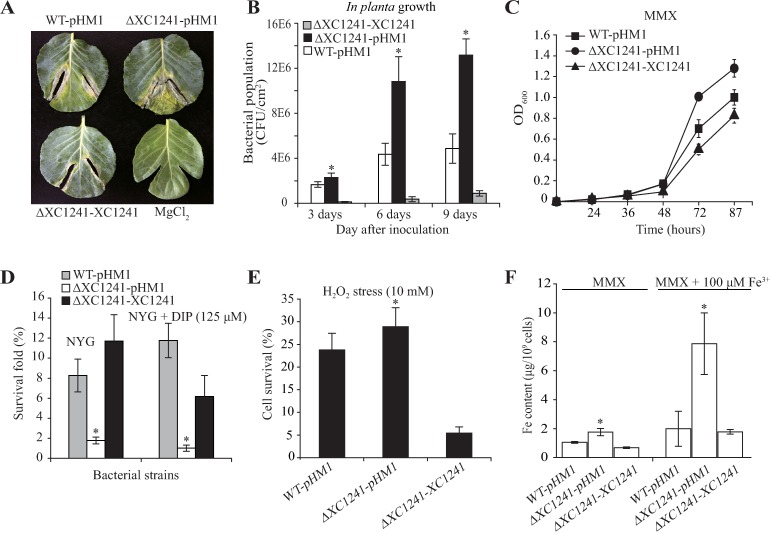
*XC1241* (*tdvA*) expression is detrimental to bacterial virulence and iron uptake under iron-depleted condition. (A) Inactivation of *tdvA* increased bacterial virulence against host cabbage (*B*. *oleraceae*). Virulence levels were recorded 8 days after inoculation. Inoculation of 1 mM MgCl_2_ was used as a negative control. (B) Bacterial population grown *in planta*. Bacterial strains were injected into plant leaves using a 1 ml syringe. Bacterial colonies were isolated from leaf disks and counted by serial dilution after inoculation. Each value is the average number calculated from six leaf disks. (C) Bacterial growth curves in iron-depleted MMX medium. Vertical bar indicates the standard deviation (n = 3). (D) Survival of bacterial strains subjected to streptonigrin treatment. Bacterial cultures (OD_600_ = 0.4) in NYG media were treated with streptonigrin (SNG, 1 μg/ml) for 16 h and the survival rates were calculated by counting the cells before and after stress. 2, 2′-dipyridyl (DIP) (125 μM) was added into the NYG medium to chelate free iron ions. Vertical bars represent the standard deviations (n = 3). (E) Survival rate of bacterial strains treated by H_2_O_2_ stress. Bacterial cultures (OD_600_ = 0.6) in NYG media were treated with H_2_O_2_ (10 mM) for 15 minutes and survival rates were calculated by counting the cells before and after stress. Vertical bars represent the standard deviations (n = 3). (F) Iron concentration in bacterial cells. Equal volumes of bacterial cells were cultured in minimal medium MMX or MMX plus 100 μM Fe^3+^. The amounts of total iron in the cells were measured using inductively coupled plasma spectroscopy (ICP-OES). Vertical bars represent the standard deviations (n = 3). In (B, D, E, and F), * indicates a significant difference (by Student’s *t*-test, P < 0.05) compared with the wild-type (WT) strain under the same test conditions.

*XC1241* encodes a 928 aa, typical TBDR with an N-terminal TBDR plug domain and a C-terminal TBDR domain; the biological function has not yet been studied experimentally. Plant inoculation showed that the bacterial virulence of a *XC1241* mutant against the host cabbage (*B*. *oleraceae*) was significantly increased, whereas overexpression of *XC1241* caused a substantial decrease in virulence (Fig 5A). The bacterial population *in planta* was also measured. After inoculation, the bacterial populations of the *XC1241* mutants were remarkably higher than those of the WT strain, whereas overexpression of *XC1241* significantly decreased the bacterial population *in planta* (Fig 5B). Correspondingly, the *XC1241* mutant had a higher growth rate than the WT and the overexpression strain when grown in iron-depleted media (MMX), albeit there was no substantial difference between the mutant and the WT strain when grown in iron-replete conditions (Fig 5C and S5 Fig).

We hypothesized that *XC1241* encodes a TBDR that is detrimental to iron uptake when bacteria are grown in an iron-depleted environment. To test this, the capability of bacterial resistance to SNG treatment and oxidative stress was measured. As shown in Fig 5D, under both iron-depleted and iron-replete conditions, the survival rates of the *XC1241* mutant were significantly lower than those of the WT strain (by 0.22- and 0.09-fold, respectively), and genetic complementation substantially restored the survival rates towards that of the WT strain (Fig 5D). In addition, the *XC1241* mutant exhibited a relatively high resistance to H_2_O_2_ challenge, while overexpression of *XC1241* resulted in extreme sensitivity to oxidative stress (Fig 5E). Based on this, the intracellular concentration of total iron in the *XC1241* mutant was determined by the ICP-OES method. It showed that under iron-depleted and iron-replete environments, the cellular iron concentrations of *XC1241* mutant were 1.77 and 7.86 μg/10^9^ cells, which were 1.68-fold and 3.95-fold higher than the WT levels, respectively. Overexpression of *XC1241* suppressed the increase in intracellular iron concentration (Fig 5F). Thus, this result confirmed that the expression of *XC1241* is detrimental to iron uptake. Inactivation of this gene promoted the bacteria’s ability to absorb extracellular iron into cells. Therefore, we named *XC1241* as *tdvA* (TonB-dependent receptor detrimental to virulence).

### Phosphorylated VgrR binds to the *tdvA* promoter with high affinity to repress its transcription

**Fig 6:**
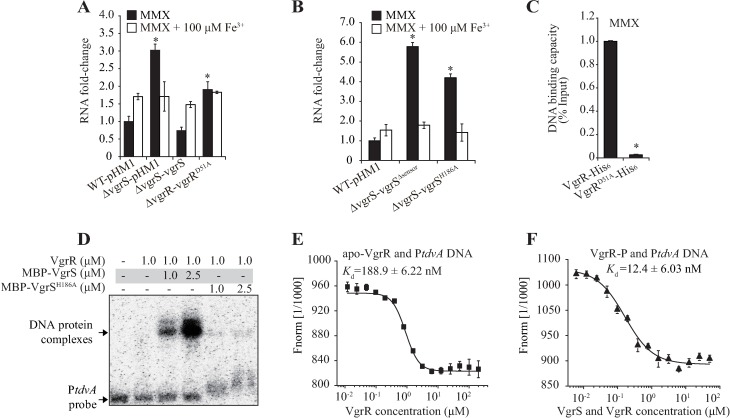
Phosphorylated VgrR represses *tdvA* transcription by binding to its promoter region with high affinity. (A and B) Dephosphorylation of VgrR and VgrS resulted in increased *tdvA* mRNA levels, as assessed by qRT-PCR. A representative of three independent experiments is shown. (C) Enhancement of the VgrR–P*tdvA* interaction by VgrR phosphorylation *in vivo*. ChIP-qPCR was conducted to quantify the enrichment of VgrR at the *tdvA* promoter. Bacteria containing a recombinant VgrR-His_6_ or a VgrR^D51A^-His_6_ were used as samples. Data are the average of three independent replicates. Vertical bars indicate the standard deviations. In (A, B and C), * indicates a significant difference (by Student’s *t*-test, P < 0.05) compared with of the wild-type (WT) strain under the same test conditions. (D) Enhancement of the VgrR–P*tdvA* interaction by VgrR phosphorylation *in vitro*. Electrophoretic mobility shift assays were used to detect the VgrR–P*tdvA* interaction *in vitro*. VgrR was phosphorylated by MBP-VgrS in the presence of ATP, as indicated. A recombinant MBP-VgrS^H186A^, whose phosphorylation site was mutated, was used as the negative control. The experiment was repeated three times. (E and F) Quantification of VgrR–P*tdvA* binding affinity by microscale thermophoresis (MST). The DNA sequence of P*tdvA* was labeled by a fluorescein (FAM, 45 nM) and incubated with VgrR in an NT standard capillary for the MST assay. (E) apo-VgrR-P*tdvA* binding affinity; (F) Phosphorylated VgrR binds P*tdvA* with a high affinity; In (E and F), three independent experiments were conducted. Vertical bars represent the standard deviation.

VgrR–VgrS is a TCS that modulates downstream gene expression through protein phosphorylation, the next question is whether the phosphorylated VgrR (VgrR-P) or unphosphorylated VgrR is the active form that represses *tdvA* transcription. To measure the effect of unphosphorylated VgrR on *tdvA* transcription, the mRNA levels of *tdvA* were measured in its cognate HK gene *vgrS* deletion mutant (Δ*vgrS*), vgrS^Δsensor^ mutant (the coding sequence of sensor region being deleted), and two amino acid variants (vgrR^D51A^ and vgrS^H186A^) with substitutions in the phosphorylation residues, respectively. Under iron-depleted conditions (MMX) the *tdvA* mRNA levels in the *vgrS* and *vgrR*^D51A^ mutants significantly increased to 300% and 190% of the level in the WT strain, respectively. In the genetic complementary strain of *vgrS*, the *tdvA* mRNA amount was restored to the WT level (Fig 6A). In the vgrS^Δsensor^ and vgrS^H186A^ mutants, the *tdvA* mRNA levels significantly increased to 580% and 420% of the level in the WT strain, respectively (Fig 6B). These genetic analyses suggested that phosphorylated VgrR-P, rather than the unphosphorylated VgrR, is the active form that represses *tdvA* transcription.

To detect how VgrR-P represses *tdvA* transcription by TF-promoter interaction, we first used ChIP-qPCR to quantify the *in vivo* VgrR–P*tdvA* occupancy under iron-depleted condition. As shown in Fig 6C, in iron-depleted conditions (MMX medium), the amount of co-immunoprecipitated P*tdvA* DNA from the sample of *vgrR*^D51A^ mutant was significantly lower than that from the WT (3% of the WT level), indicating that the total amount of unphosphorylated VgrR binding to P*tdvA* significantly decreased. To verify this biochemically, *in vitr*o EMSA was used to measure the binding between P*tdvA* and VgrR with different phosphorylation states. As shown in Fig 6D, in the presence of ATP, phosphorylation of VgrR by HK VgrS resulted in a substantial increase in the signal intensity that represents the VgrR–P*tdvA* complex. When a recombinant form of VgrS whose phosphorylation His residue being substituted (VgrS^H186A^) was added, the intensity of the bands representing the VgrR–P*tdvA* complex (lanes 5 and 6) were similar to that of the control using unphosphorylated VgrR (lane 2, Fig 6D). Thereafter, the VgrR–P*tdvA* binding affinity was quantified using microscale thermophoresis (MST) with 5′-FAM-labeled P*tdvA* DNA as the probe. Phosphorylation of VgrR by VgrS remarkably decreased the disassociation constant value (*K*_d_) of VgrR–P*tdvA* binding from 188.9 ± 6.22 nM (VgrR-P*tdvA* binding, Fig 6E) to 12.4 ± 6.03 nM (phosphorylated VgrR-P*tdvA* binding, Fig 6F). Taken together, these *in vivo* and *in vitro* analyses strongly support the view that phosphorylation of VgrR promotes the ability to bind to the promoter of *tdvA*, resulting in repression of *tdvA* transcription.

### VgrS binds iron by its sensor region to detect iron depletion

**Fig 7:**
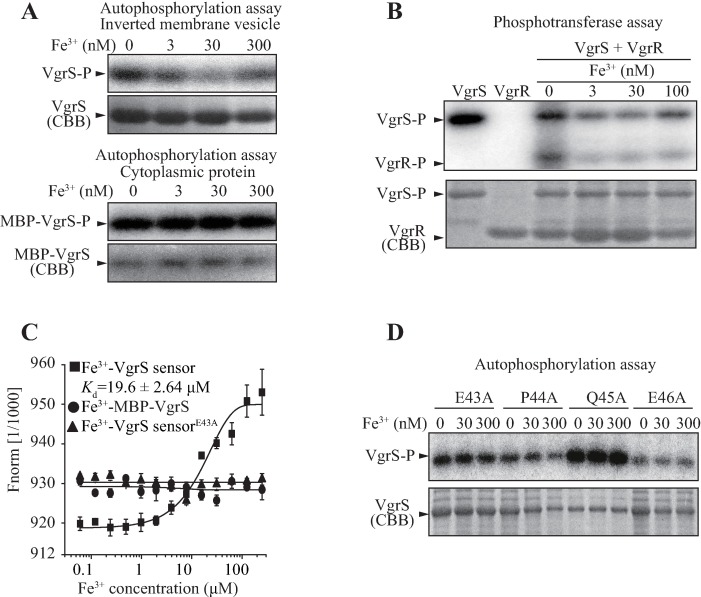
VgrS senses Fe^3+^ depletion by its sensor region. (A) Fe^3+^ inhibits the autophosphorylation of full-length VgrS. Upper panels: Inverted membrane vesicles containing full-length VgrS were phosphorylated with 100 μM ATP containing 10 μCi [γ-^32^P]ATP. Fe^3+^ was added at different concentrations. Lower panels: Soluble, truncated VgrS is not stimulated by Fe^3+^. MBP-VgrS without the input and transmembrane domains was used in the autophosphorylation assay. The experiment was repeated three times. (B) Iron excess decreased the phosphotransfer level from VgrS to VgrR. Full-length VgrS membrane was phosphorylated as described in (A) for 2 min in the presence of Fe^3+^, 15.0 μM VgrR was added into the mixture for 20 sec before stopping the reaction. (C) VgrS sensor directly binds Fe^3+^. 2 μM VgrS sensor, truncated VgrS (MBP-VgrS) and VgrS^E43A^ sensor were used in a microscale thermophoresis (MST) assay. The titer of Fe^3+^ ranged from 0.061 to 250 μM. The experiment was repeated three times. (D) Substitution of residues in the ExxE motif of VgrS sensor eliminated VgrS’s sensing of Fe^3+^. Inverted membrane vesicles with full-length VgrS^E43A^, VgrS^P44A^, VgrS^Q45A^ and VgrS^E46A^ were used in the autokinase assay as in (A). Fe^3+^ was added into the reaction mixtures. The experiment was repeated twice. In (A, B, and D), the reaction was stopped by adding loading buffer before SDS-PAGE separation and autoradiography. The gel was stained by Coomassie brilliant blue (CBB) to check the amount of proteins.

The fact that VgrR-P plays an important role in controlling *tdvA* transcription suggests that its cognate HK, VgrS, is a receptor that detects iron starvation and responds accordingly to modulate the VgrR phosphorylation level. To investigate biochemically whether VgrS responds to iron stimulation, membrane-bound VgrS was used in an *in vitro* phosphorylation assay. As shown in Fig 7A (upper panel), in the absence of Fe^3+^, the phosphorylation level of VgrS (VgrS-P) is high. However, when the physiological concentration of Fe^3+^ increased (3–300 nM), the level of VgrS-P remarkably decreased. As a control, the truncated cytoplasmic fragment of VgrS exhibited an autokinase activity that did not alter in response to Fe^3+^ stimulation (Fig 7A, lower panel), which suggested that the signal input region, rather than the cytosolic DHp and CA regions, detects the Fe^3+^ concentration. In addition, VgrS-VgrR phosphotransfer assay showed that addition of Fe^3+^ resulted in remarkable decreases in both VgrS-P and VgrR-P levels (Fig 7B).

VgrS encodes an N-terminal, periplasmic sensor domain that is located putatively in the bacterial periplasm (Fig 1A). The ligand or signal detected by this sensor region is unknown. To determine whether VgrS sensor interacts directly with Fe^3+^, the sensor peptide was expressed and purified, and an MST assay revealed that the VgrS sensor binds Fe^3+^ with a disassociation constant of 19.6 ± 2.64 μM (Fig 7C), but did not bind Fe^2+^ (S6 Fig). Meanwhile, the cytoplasmic region of VgrS did not bind Fe^3+^ (Fig 7C). To identify the possible Fe^3+^ binding site, a multiple sequence alignment revealed an ExxE motif (EPQE) within the VgrS sensor. This motif was demonstrated as a metal binding site in other proteins, such as BqsS in *Pseudomonas aeruginosa*, PmrB in *Salmonella enterica*, HbpS in *Streptomyces reticuli*, and FTR1 in *Saccharomyces cerevisiae* [23–26]. Therefore, we expressed four full-length VgrSs, each with a substitution of VgrS^E43A^, VgrS^P44A^, VgrS^Q45A^, or VgrS^E46A^, and then extracted the inverted membrane vesicles containing them. *In vitro* phosphorylation assays showed that these recombinant proteins were immune to iron stimulation (Fig 7D). Based on this result, we expressed and purified a recombinant VgrS sensor of which the 43rd Glu residue was substituted by Ala (VgrS sensor^E43A^). In addition, an MST assay showed that this substitution resulted in disassociation between the recombinant sensor and Fe^3+^ (Fig 7C).

Taken together, the above results revealed that VgrS is a membrane-bound receptor that detects iron starvation via its sensor region. Under growth conditions with limited iron, the phosphorylation level of VgrS is high because it is not inhibited by iron, and can then catalyze the phosphorylation of VgrR. Increased phosphorylation of VgrR causes the TF to bind P*tdvA* to repress the transcription of *tdvA*.

### Iron repletion releases VgrR repression on *tdvA* transcription by disassociating both VgrR-DNA and VgrR-VgrS binding

**Fig 8:**
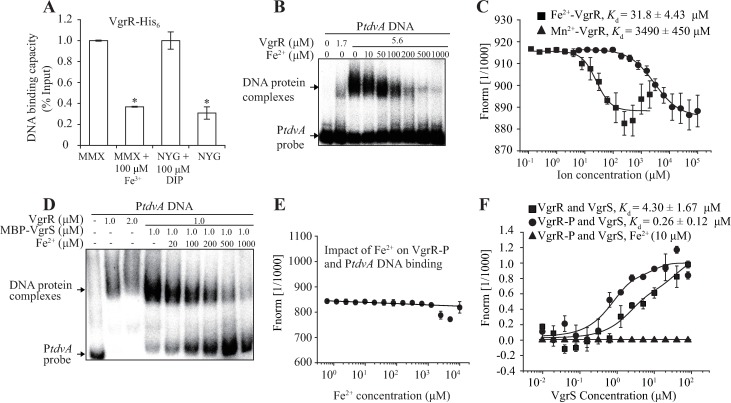
Iron-VgrR binding disassociates VgrR-DNA and VgrR-VgrS interactions. (A) The amount of VgrR–P*tdvA* binding was decreased in iron-replete conditions. ChIP-qPCR was conducted to quantify the enrichment of VgrR at the *tdvA* promoter *in vivo* when bacteria were grown under iron-replete and iron-depleted conditions, respectively. The experiment was repeated three times. Vertical bars indicate the standard deviations. (B) The presence of Fe^2+^ inhibits the formation of the VgrR–P*tdvA* complex *in vitro*. Electrophoretic mobility shift assays were conducted to determine the impact of Fe^2+^ on VgrR–P*tdvA* binding. The concentrations of Fe^2+^ were gradually increased from 10 μM to 1.0 mM. The assay was repeated independently three times. (C). Fe^2+^ directly binds VgrR. 2 μM VgrR and Fe^2+^ or Mn^2+^ was used in an MST assay. The titer of Fe^2+^ ranged from 0.12 μM to 2 mM. The titer of Mn^2+^ ranged from 6.1 to 100 mM. (D) Addition of ferrous iron disassociates the binding between phosphorylated VgrR and P*tdvA*. Electrophoretic mobility shift assays were conducted to determine the impact of Fe^2+^ on VgrR–P*tdvA* binding. VgrR was phosphorylated by MBP-VgrS and ATP. The concentrations of Fe^2+^ were gradually increased from 20 μM to 1.0 mM. The assay was repeated independently three times. (E) Ferrous iron disassociates the interaction between phosphorylated VgrR and P*tdvA*. 5′-FAM labelled P*tdvA* DNA was used in the MST assay. Different concentrations of Fe^2+^ (0.61 μM -10 mM) were added to the mixture as indicated. (F) Ferrous iron disassociates the interaction between VgrR and VgrS. VgrR protein was labelled in the MST assay. If needed, VgrR was phosphorylated by acetyl phosphate. Different concentrations of VgrS membrane were added to the mixture as indicated. In (C, E and F), the experiment was repeated independently three times.

Repression of *tdvA* transcription by VgrR-P aids the uptake of extracellular iron into bacterial cells (Fig 5). However, if the cellular iron concentration reaches physiological levels, the metal is toxic to the cells. As shown in Fig 3C (black column) and Fig 6A (white column), under iron-replete conditions, inactivation of *vgrS* or *vgrR* did not significantly affect *tdvA* transcription levels, which suggested that the regulatory relationship between VgrR and P*tdvA* is dissociated. To quantify the VgrR occupancy on the P*tdvA* promoter under iron-replete conditions, ChIP-qPCR analyses revealed that either in minimal MMX medium plus iron or in the rich medium NYG (both are iron-replete), significantly less P*tdvA* DNA could be co-immunoprecipitated with VgrR compared with the sample of bacteria grown in iron-depleted conditions (MMX or NYG plus the iron chelator DIP, Fig 8A). This *in vivo* evidence suggested that there is less VgrR–P*tdvA* interaction in an excess iron environment. Based on this, *in vitro* EMSA was used to observe the effect of ferrous iron on the VgrR–P*tdvA* interaction. As shown in Fig 8B, when Fe^2+^ was absent from the reaction mixture, the intensity of VgrR–P*tdvA* binding signal was the highest. However, increasing the iron concentration gradually decreased the signal representing VgrR–P*tdvA* complex. In addition, an MST assay demonstrated that VgrR indeed binds directly to Fe^2+^, with a *K*_d_ value of 31.8 ± 4.43 μM. As a control, the protein did not bind to Mn^2+^ (*K*_d_ value > 3.4 mM) (Fig 8C).

Since the binding affinity of VgrR–P*tdvA* interaction is significantly increased if the VgrR is phosphorylated (Fig 6D), it prompts a question that whether the accumulation of Fe^2+^ impacts the phosphorylated VgrR-DNA relationship. As shown in Fig 8D, *in vitro* EMSA assay revealed that even if VgrR was phosphorylated by VgrS, along with the increase of Fe^2+^ concentrations in the mixtures, the amounts of VgrR-P*tdvA* complex were substantially decreased (Fig 8D). MST quantification of binding affinity using 5′-FAM labelled P*tdvA* DNA as a probe also showed that addition of Fe^2+^ dissociated the VgrR-P*tdvA* interaction regardless of the phosphorylating state of VgrR (Fig 8E). In addition, when the VgrR protein was labelled and used in the MST assay to quantify its interaction with membrane-embedded, full-length VgrS, the results revealed that VgrR and VgrS directly interacted with a *K*_d_ value of 4.30 ± 1.67 μM (Fig 8F). Phosphorylation of VgrR by acetyl phosphate significantly increased the VgrR-VgrS binding affinity to a *K*_d_ value of 0.26 ± 0.12 μM. However, when the Fe^2+^ of physiological concentration level (10 μM) was added into the mixture, the VgrR-VgrS binding was completely dissociated even if the VgrR was phosphorylated (Fig 8F).

Collectively, both *in vivo* and *in vitro* results suggested that if the intracellular Fe^2+^ is high, the metal directly binds to the VgrR. This process not only impedes the binding between VgrR and P*tdvA* but also between VgrR and VgrS, regardless of the phosphorylation or dephosphorylation states of VgrR. As afore-mentioned, disassociation of VgrR from P*tdvA* releases the repression of *tdvA* transcription, which is deleterious to continuous iron uptake of the bacterium.

## Discussion

**Fig 9:**
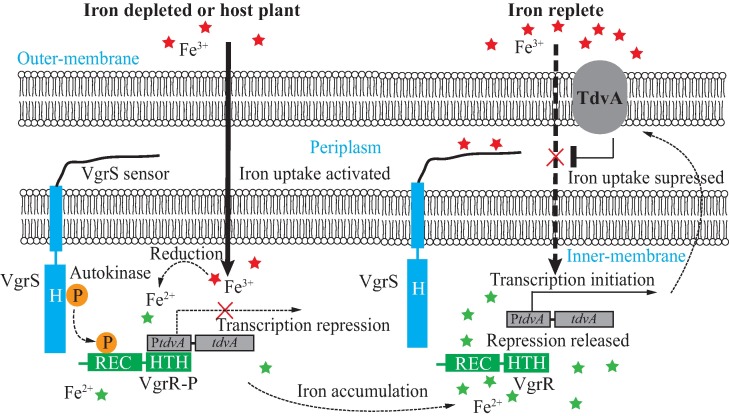
Molecular model for VgrS–VgrR regulated iron homeostasis by repressing *tdvA* transcription. P in the circle represents a phosphoryl group. Under iron-depleted or host plant environments, *tdvA* transcription is repressed by VgrR-P, which is require for the uptake of extracellular iron. Under the iron-replete conditions, the repression of *tdvA* transcription by VgrR is released.

How pathogenic bacteria detect iron concentrations and react adaptively is critical to successful infection. The molecular mechanism regulating this process is fragmentary. For example, previously identified iron-binding bacterial transcription factors, including proteins belonging to the MerR or DtxR family, are not membrane-bound sensors that detect extracytoplasmic iron. Previous reports revealed that Fur is a critical cytoplasmic regulator of iron homeostasis in *X*. *campestris* as in other bacteria [16, 27]. Our study revealed that in *X*. *campestris* pv. *campestris*, VgrS is a membrane-bound HK that monitors iron scarcity directly via its N-terminal sensor (Fig 9). Under low iron concentrations, suppression of the VgrS autophosphorylation by Fe^3+^ is relieved, resulting in VgrR phosphorylation. The activated VgrR then modulates the expression of a number of genes as identified by comparative proteomic and ChIP-seq approaches. Among these downstream genes, we found that the phosphorylated VgrR binds to the promoter region of a TBDR gene, *tdvA*, to repress its transcription. Repression of *tdvA* transcription is a prerequisite for the uptake of extracellular iron because the expression of *tdvA* is detrimental to efficient iron uptake under iron depleted conditions or in host plants. However, along with the elevation of intracellular Fe^2+^, excessive Fe^2+^ binds to VgrR and impedes its association with the *tdvA* promoter, which releases the repression of VgrR on *tdvA* transcription (Fig 9). In addition, our study identified the consensus binding DNA motif of VgrR. These results support a regulatory mechanism in which VgrR–VgrS is a prominent system of *X*. *campestris* pv. *campestris* that detects both extracellular and intracellular iron, and responds adaptively to maintain iron homeostasis. To our knowledge, VgrS is the first biochemically identified membrane-bound receptor to sense environmental stimuli in bacterial species belonging to the genus *Xanthomonas*.

Over the past three decades, the biochemistry of phosphorylation and the regulation of TCSs have been extensively documented. However, how HKs detect environmental stimuli, including metals, remains poorly understood. For example, as one of the prominent model organisms in studying plant pathology, *X*. *campestris* pv. *campestris* encodes 32 orthodox and 20 hybrid type HKs [28]. Only the HK RpfS was biochemically proved to detect a diffusible factor (DSF) eliciting cell–cell communication [29]. RpfS is a soluble, cytoplasmic HK without transmembrane helix so that it is likely a sensor to detect intracellular signals. For other HKs, especially those bound in the membrane to detect extracellular stimuli, no corresponding signals or ligands have been biochemically identified (including RpfC that was proposed to detect DSF by genetic analyses [30, 31]). VgrR–VgrS is a TCS controlling bacterial growth, production of extracellular protease and virulence. In this study, we demonstrated that VgrS is an HK that detects extracytoplasmic iron scarcity via its sensor region based on the following evidence: 1) High Fe^3+^ concentrations inhibited VgrS autophosphorylation, whereas under low Fe^3+^, VgrS phosphorylation level substantially increased (Fig 7A). 2) Increased levels of VgrS-P trigger the VgrR-P regulation of downstream gene expressions, including the repression of *tdvA* transcription and activation of iron uptake associated genes (Fig 6A and 6B). 3) Fe^3+^ binds directly to the ExxE motif within the VgrS sensor, whereas both *in vivo* and *in vitro* evidence showed that point mutations within the ExxE coding sequence not only eliminated sensor–Fe^3+^ binding, but also made VgrS immune to Fe^3+^ stimulation (Fig 7C and 7D). In *Salmonella enterica*, the HK PmrB, which contains ExxE motifs within periplasmic domain, has been found to detect ferric iron [9, 32, 33]. Comparison of PmrB and VgrS indicate they are different in terms of ligand-stimulated activation. The VgrS and PmrB sequences are 26% identical (BlastP search), and they share a similar architecture of domain organization, with an N-terminal periplasmic sensor, a HAMP linker and a C-terminal transmitter domain. However, activation of the PmrB autophosphorylation required a high concentration of Fe^3+^ (10–100 μM), which seems unlikely to exist in other host tissues, except the stomach and small intestine [9, 34]. Therefore, PmrB might detect high concentrations of iron when bacteria are in the digestive tract or soil [35]. In contrast to PmrB, VgrS maintains a higher level of phosphorylation under iron-depleted conditions compared with iron-replete condition: it is iron scarcity, rather than a high level of iron, which activates the VgrS autophosphorylation. In host plant tissues, although the physiological concentration of iron is around 0.1–10 μM, the majority of the iron is chelated by ferritin, reductases and phytosiderophores, or translocated into subcellular organelles, such as mitochondria or chloroplasts [36, 37]. This creates a low iron environment to activate VgrS to trigger the adaptive response to take up iron and promote bacterial growth in the host. However, the structural mechanism determining the activation of PmrB and VgrS by different iron concentrations remains to be investigated.

As Fig 1 showed, the phenotypic changes of the *vgrR* and *vgrS* were not completely coincident. For example, Co^2+^ stress caused difference in the growth of the two mutants (Fig 1B), and the cellular iron concentration in the *vgrS* mutant decreased much lower than that of the *vgrR* mutant when bacteria were grown under iron-replete condition (Fig 1F and 1G). One of the possible reasons to cause this inconsistency is that in TCS regulation, the RR is usually phosphorylated by other small molecular phospho-donors besides of its cognate HK, such as acetyl phosphate in cells [8]. In addition, VgrR-VgrS may interact with other TCSs in regulation. In *X*. *campestris* pv. *campestris*, VgrR-VgrS has two paralogous systems that were generated by operon duplication in genomic evolution: XC3126–XC3125 and XC3452–XC3451; Among them, the HK XC3125 also contains a ExxE motif within its putative periplasmic sensor region. However, the biological functions of these paralogs are unknown. During evolution, gene duplication usually resulted in the functional divergence of paralogs through sub-functionalization, neo-functionalization, or generating pseudogenes [38]. In TCSs, it has been found that paralogous TCSs cross-talk through multiple modes, including phosphotransfer, heterodimerization, and epistatic regulation [39, 40]. Our preliminary investigation revealed that VgrR-VgrS interacts with the other two paralogs in modulating stress response and bacterial virulence. In-depth dissection of this regulatory network will help to elucidate the complexity of the VgrR-VgrS regulation.

The ExxE motif acts as an iron-binding site in various proteins, such as HKs, iron transporters, and iron-binding proteins [23–26, 41, 42]. It is noticeable that the periplasmic sensor of PmrB contains two ExxE motifs to detect Fe^3+^, rather than Fe^2+^ [9, 35]. Although the VgrS sensor only has a single ExxE motif, it has a similar specificity to PmrB in sensing Fe^3+^. MST analysis revealed that the VgrS sensor did not bind Fe^2+^ (S6 Fig). However, in *Pseudomonas aeruginosa*, an HK, BqsS, was observed to contain a single ExxE motif in the sensor region to sense Fe^2+^ directly [10, 23]. These results suggested that the number of ExxE motifs may not be important to discriminate Fe^2+^/Fe^3+^; it is possible the sequence context around the ExxE motif is critical to determining the specificity of peptides in recognizing different iron ions. In addition, as shown in Fig 1B, other metal ions, such as copper, zinc and manganese also impacted the growth of *vgrR* and *vgrS* mutants, suggesting they have direct or indirect relationships with VgrR-VgrS system. Future work is needed to answer whether VgrS senses these metals.

RR is in the center of TCS regulation as it controls the expression of downstream genes or the behavior of cellular machineries [8]. As shown in Fig 9, we found that two intrinsic biochemical properties of VgrR control the de-repression of *tdvA* transcription: VgrR dephosphorylation and by formation of the VgrR-Fe^2+^ complex. The two pathways lead synergistically to dissociation between VgrR and P*tdvA* and activation of *tdvA* expression. Consequently, compared with prototypical RRs, the activity of VgrR is regulated not only by VgrS-mediated phosphorylation, but also by metal binding. However, because iron-VgrR binding disassociates the interaction between VgrR-DNA and VgrR-VgrS regardless of the phosphorylation state of VgrR (Fig 8E and 8F), we propose that when the intracellular iron is excessive, the binding of Fe^2+^-VgrR provides a faster and more efficient mechanism to disaggregate the VgrR-P*tdvA* interaction even if bacteria live in iron-depleted environments. Therefore, this process is a counter reaction to VgrS-P-triggered iron uptake to avoid continuous toxic accumulation of Fe^2+^ in the bacterial cytosol. In addition, the VgrR regulon was dissected by proteomic and ChIP-seq analyses (S3–S7 Tables). Besides of *tdvA*, these analyses revealed that VgrR is a global regulator that controls, directly or indirectly, the expressions of hundreds of genes. A number of genes known to be critical in bacterial virulence were identified, such as genes encoding type II secretion system (XpsE, XpsI and XpsN), type III secretion system (HrpB8), extracellular enzymes (XC0639 and XC3378), virulence-related pathways, such as the shikimate-chorismic acid pathway, biosynthesis of exopolysaccharides and aromatic amino acids, detoxification enzymes, and a recently identified periplasmic endopeptidase, Prc [43], suggesting that VgrR is a canonical regulator in virulence. Meanwhile, VgrR controls the transcription of other TCS genes, transcription factors, and alternative sigma factors (S3–S5 Tables). This implies that VgrR is central to the signaling network of *X*. *campestris* pv. *campestris*, but its regulatory role in this important phytopathogenic bacterium requires further investigation.

Gram-negative bacteria usually encode several to hundreds of TBDRs [44]; however, the functional diversity of these TBDRs remains unclear [45]. The present work identified that TdvA is a special TBDR. Genetic analysis revealed that inactivation of *tdvA* increased bacterial virulence against the host plant and promoted iron uptake into cells (Fig 5). TdvA is a putative outer membrane transporter with a typical secondary structure of a TBDR. However, under iron-depleted conditions or in the host environment, the expression of *tdvA* is detrimental to uptake extracellular iron and bacterial virulence, such that tight repression of *tdvA* by phosphorylated VgrR is necessary for bacterial growth (Figs 5 and 6). The biochemical function of TdvA and its substrate is unknown. We proposed several possible biochemical functions of TdvA: 1) TdvA might be an inhibitor to other TBDR during iron uptake process. In Gram-negative bacteria, TBDRs bind to an energy-transducing complex TonB-ExbB-ExbD by the C-terminus of TonB, which transduces energy to TBDRs to uptake iron into the periplasm. Since the amount of TonB protein is limited in bacterial cells [46, 47], one of the possibility is that TdvA competes with other TBDR to bind TonB when bacteria are grown in iron-depleted conditions. 2) TdvA might have a competitive relationship with other TBDRs when localized to a particular site of the outer-membrane. 3) The substrate imported by TdvA is not an iron-containing siderophore, but another chemical that is antagonistic to iron uptake. For example, in *E*. *coli*, Kadner et al. found a TBDR, BtuB that takes up vitamin B_12_. The process inhibited the uptake of a siderophore ferrichrome [46]. Therefore, further biochemical and electrophysiological investigations are necessary to elucidate the role of the TdvA family of TBDRs during bacterial pathogenesis.

In conclusion, based on the dissection of VgrS-VgrR regulon, the present study revealed that this TCS detects both extracytoplasmic iron scarcity and intracellular iron excess to modulate bacterial physiology in the host plant and iron-depleted environments. Future studies are necessary to investigate the molecular mechanism of metal recognition of the VgrS, and the functional divergence among the HK and its paralogs that are derived from gene duplications during bacterial evolution.

## Materials and Methods

### Bacterial strains, plasmids, and growth conditions

The bacterial strains and plasmids used in this study are listed in S1 Table. *X*. *campestris* pv. *campestris* strains were routinely cultivated at 28°C in rich NYG medium (tryptone 5 g/L, yeast extract 3 g/L, glycerol 20 g/L, pH 7.0), iron-depleted minimal MMX medium [glucose 5g/L, sodium citrate, 1.0 g/L, (NH_4_)_2_SO_4_ 2 g/L, K_2_HPO_4_ 4g/L, KH_2_PO_4_ 6.0 g/L, MgSO_4_ 0.2 g/L, pH 7.0], or iron-replete MMX medium (MMX plus 100 μM FeCl_3_). *Escherichia coli* strains were grown aerobically at 37°C in LB medium. As necessary, antibiotics were added at the following concentrations: for *X*. *campestris* pv. *campestris*, rifampicin (25 μg/ml), spectinomycin (150 μg/ml), tetracycline (5 μg/ml) and kanamycin (50 μg/ml); for *E*. *coli*, spectinomycin (150 μg/ml), kanamycin (50 μg/ml), ampicillin (100 μg/ml) and tetracycline (20 μg/ml). 2, 2′-dipyridyl (DIP), an iron chelator, and streptonigrin (SNG, Sigma, St. Louis, MO USA), were prepared as a 1 M and 1 mg/ml stocks, respectively, before use. Preparation of bacterial competent cells and transformation by electroporation were performed according to previous reports [17, 48].

### Bacterial genetic manipulation

Construction of in-frame deletion and marker exchange mutants were based on the homologous double-crossover method, according to previous studies, using the suicide vector pK18mobSacB [17, 48]. Construction of the insertion inactivation mutant of *tdvA* was based on the homologous, single cross-over method using suicide vector pK18mob. Genetic complementation or overexpression of genes was performed by constructing recombinant vectors pHM1 or pHM2, which were provided *in trans* into corresponding bacterial strains. All primer sequences used in this study are listed in S2 Table.

### Phenotypic characterization of bacterial strains

Bacterial tolerance to various metal stresses was performed by adding 2.5 mM FeSO_4_ (plus vitamin C 5 mM), 1.5 mM FeCl_3_, 0.4 mM ZnSO_4_, 0.3 mM CuSO_4_, 0.3 mM CoCl_2_, 0.5 mM NiSO_4_, or 2.6 mM MnSO_4_, respectively, into the NYG agar. The metal concentrations were lower than the minimal inhibitory concentrations that were determined by experiments. Bacteria stains were grown on the plates for 48–72 hours. For virulence assays, the host was 8-week-old cabbage (*Brassica oleraceae* cv. Jingfeng 1). Plant inoculation and *in planta* bacterial growth assays were carried out according to previous reports [15, 17].

To determine the sensitivity of bacterial strains to streptonigrin treatment, strains were grown in NYG medium to OD_600_ = 0.6, and streptonigrin was added to a concentration of 1 μg/ml. After incubation at 28°C without shaking for 16 h, bacterial numbers were estimated by serial dilution. The percent of inhibition of growth was calculated by comparing the bacterial numbers to those of the no streptonigrin control. To determine H_2_O_2_ susceptibility, 10 mM of H_2_O_2_ was added into bacterial cultures (OD_600_ = 0.6) for 15 min. The bacterial numbers were then determined by serial dilution and the relative survival rate was calculated by comparing the cell number with the no H_2_O_2_ control.

### Determination of cellular iron concentration

Cellular iron levels in different strains were determined by inductively coupled plasma-optical emission spectroscopy (ICP-OES, PerkinElmer, Waltham, MA USA), as reported previously [49, 50]. The bacterial cells were grown to an OD_600_ of 1.0 in NYG medium. Cells were collected by centrifugation and washed three times with fresh MMX medium. The bacteria were then inoculated into fresh MMX or MMX plus 100 μM FeCl_3_ media (OD_600_ = 0.6). After 3 h of growth, cells were harvested by centrifugation and washed three times with sterilized PBS buffer (KH_2_PO_4_ 0.27 g/L, Na_2_HPO_4_ 1.42 g/L, NaCl 8g/L, KCl 0.2 g/L, pH 7.4). The pelleted cells were oven-dried at 65°C for 72 h, and digested with HNO_3_–HClO_4_ (4:1, v/v). The digest was then transferred to a 25 ml volumetric flask and made up to 25 ml with H_2_O. The iron atom content was then measured by ICP-OES. Samples not treated by HNO_3_-HClO_4_ (4:1) were used as parallel controls. The total iron concentration was calculated by dividing the iron atom value by the number of bacterial cells.

### RT-PCR and qRT-PCR analysis

Total bacterial RNA was extracted using the TRIzol reagent (Invitrogen, Waltham, MA USA). Contaminating DNA was digested by RNase-free DNase I (Ambion, Waltham, MA USA). cDNA was synthesized using random primers (Promega, Madison, WI USA) and Superscript III reverse transcriptase (Invitrogen). To analyze the *vgrR-vgrS* operon structure, a series of primers were used to amplify the possible transcripts from the intergenic region between genes (Fig 1A), using amplification of the *vgrS* cDNA as the positive control.

The mRNA level of *tdvA* was quantified by qRT-PCR using Maxima SYBR Green (Fermentas, Waltham, MA USA) in a DNA Engine Option 2 System (Bio-Rad, Hercules, CA USA), according to the manufacturer’s instructions. Amplification of 16S rRNA was used as loading control. Generally, a qRT-PCR experiment was repeated independently three times, with three technical repeats of each sample. A representative of all the biological repeats was selected and reported.

### GUS reporter activity assay

To construct the promoter-GUS transcriptional fusion, DNA sequences corresponding to the P1, P2, P1+P2 of the *vgrR*-*vgrS* promoter, or P*tdvA* of the *XC1241* (*tdvA*) promoter were amplified and ligated to the *gusA* gene, with the Shine-Dalgarno sequence of *gusA* as the ribosome-binding site. The inserts were ligated into vector pHM2 and transformed into bacterial strains. For GUS activity assays, bacterial cells were collected by centrifugation and stored in liquid nitrogen immediately. Cells were resuspended by 1 × GUS extraction buffer (50 mM sodium phosphate pH 7.0, 5 mM DTT, 1 mM EDTA pH 8.0), lysed by sonication, centrifuged and the supernatants were used for GUS activity assays. The GUS activity was measured by the fluorometric method, using 4-methylumbelliferyl ß-D-glucuronide (MUG, Goldbio, St. Louis, MO USA) as the substrate. A standard curve was established by diluting the 4-MU (Sigma) stock solution. The fluorescence of samples and standard curve solutions were measured using an excitation wavelength of 360 nm and an emission wavelength of 460 nm.

For GUS activity quantification of bacterial cells grown *in planta*, bacteria containing a P*tdvA*-GUS fusion *in trans* provided by vector pHM2 vector were used. The bacteria were inoculated into leaves by a clipping. After 3, 5 and 10 days, six inoculated leave disks were homogenized in sterile H_2_O and proteins were extracted using GUS extraction buffer. The supernatant assay for GUS activity is as mentioned above. Bacterial cell numbers in the aqueous solution were calculated.

### Protein expression, purification and extraction of inverted membrane vesicles of VgrS

A prokaryotic expression system with vector pET30a and *E*. *coli* BL21(DE3) (Novagen, Darmstadt, Germany) was used to express recombinant full-length VgrS, truncated VgrS without the input domain, and VgrR. A C-terminal His_6_ epitope was fused to these proteins. The vector pMal-p2X (NEB, New England, UK) was used to express the truncated VgrS (MBP-VgrS) protein. His_6_-tagged proteins were expressed and purified by affinity chromatography using Ni-NTA agarose beads (Novagen) according to the manufacturer’s instructions.

Inverted membrane vesicles (IMVs) containing full-length VgrS were prepared according to a published method [48, 51]. In brief, bacteria were homogenized by sonication, and the membrane containing VgrS was collected by ultracentrifugation at 200,000 × g for 60 min at 4°C. The membranes were washed in high-salt buffer (20 mM sodium phosphate, pH 7.0, 2 M KCl, 10% glycerol, 5 mM EDTA, 5 mM DTT, 1 mM PMSF). IMVs were pelleted by ultracentrifugation (410,000 × g; 20 min; 4°C), resuspended in 0.2 mL storage buffer (20 mM Tris-HCl, pH 7.5, 10% glycerol) and used for in vitro phosphorylation assays.

### *In vitro* phosphorylation assays

For autokinase activity assays, IMVs containing full-length VgrS or truncated VgrS were incubated with 100 μM ATP containing 10 μCi [γ-^32^P]ATP (PerkinElmer) in 20 μl of reaction buffer (50 mM Tris-HCl, pH 7.8, 25 mM NaCl, 25 mM KCl, 5 mM MgCl_2_) for 2–20 minutes at 28°C. If needed, FeCl_3_ (3–300 nM) was added into the reaction mixture before addition of ATP to detect its effect on the VgrS autophosphorylation.

To detect the phosphotransferase activity of VgrS towards VgrR, VgrS was autophosphorylated as above, and then 20–80 μM of purified VgrR or VgrR^D51A^ was added into the reaction mixture, which was incubated at 28°C for the appropriate time. The reaction was stopped with 6 × SDS-PAGE loading buffer. The phosphorylated proteins were separated by 12% SDS-PAGE. After electrophoresis, SDS-PAGE gels were placed in a Ziploc bag and exposed to a phosphor screen for 1 h. The screen was scanned with a PhosphorImage system (GE Healthcare, Chicago, IL USA). After autoradiography, gels were stained by Coomassie brilliant blue to check the protein amounts.

### Primer extension

Total RNA was extracted and purified using Trizol (Invitrogen). A primer within *vgrR* gene (S2 Table) was labeled by [γ-^32^P]ATP at 37°C using T4 polynucleotide kinase (NEB) for 1 h. cDNAs of the samples from the WT strain and *vgrR* mutant (negative control) were synthesized by the labeled primer using Superscript III reverse transcriptase (Invitrogen). The reverse transcription reaction temperatures was set at 42°C and 52°C, respectively. The DNA sequencing ladders were generated using SequiTherm EXCEL II DNA sequencing Kit (Epicentre, Madison, WI USA), according to the manufacturer’s instructions. All samples were run on a 6% PAGE gel supplemented with 7% urea. After electrophoresis, the gel was placed in a Ziploc bag and exposed to a phosphor screen (GE Healthcare) for 1 h. The screen was scanned with a PhosphorImage system (GE Healthcare).

### Two-dimensional gel electrophoresis and MS identification

Two-dimensional gel electrophoresis (2-DE) was carried out as described previously [43, 52]. Briefly, before 2-DE, bacterial strains were grown in NYG, MMX and MMX plus 100 μM FeCl_3_ media. Cells were collect by centrifugation. Pellets were resuspended in 10 mL PBS containing 1 mM PMSF and immediately sonicated for 20 min to lyse the cells. The proteins were then purified with phenol saturated with Tris-HCl (pH 8.6), precipitated with an equal volume of 0.1 M ammonium acetate in methanol at −20°C. The pellets were washed twice with ice-cold 0.1 M ammonium acetate in methanol and twice with ice-cold 80% acetone. The proteins were resuspended to isoelectric focusing (IEF) buffer [7 M urea, 2 M thiourea, 4% CHAPS, 40 mM dithiothreitol, and 2% (v/v) IPG buffer (pH 4.0–7.0)]. Proteins were quantified using a 2-D Quant kit (GE Healthcare), following the manufacturer’s instructions.

For first dimension IEF, 500 μg of total protein was applied to a 24 cm pH 4–7 Dry Gel Strip (GE Healthcare). For the second dimension electrophoresis, strips were separated on 12.5% SDS-PAGE gels. Differentially abundant proteins were identified by comparing the spot abundance between the WT and the mutant (> 1.5-fold). Selected protein spots were rinsed, destained and digested with trypsin (Sigma). Afterward, the digested protein samples were identified directly using an ABI MALDI-TOF/TOF 4700 mass spectrometer (Applied Biosystems, Carlsbad, CA USA). Differentially abundant proteins were identified using the manufacturer’s software. Similarity searches were performed on a local *X*. *campestris* pv.*campestris* 8004 database.

### Electrophoretic mobility shift assay (EMSA)

To detect VgrR–DNA binding, PCR products of the corresponding promoter region or chemically synthesized double-stranded DNA probes were labeled by [γ-^32^P]ATP using T4 polynucleotide kinase (NEB), and purified using a ProbeQuant G-50 column (GE). Binding reactions were carried out in a 20-μl volume of reaction buffer [10 mM Tris-HCl, pH 7.5, 50 mM KCl, 1 mM DTT, 1 μl 50 ng/μl poly(dI-dC)]. Labeled DNA probe (2–4 fmol) and 2–6 μM VgrR were used in EMSA. For competition, a certain amount of unlabeled DNA probe was co-incubated for 20 min at room temperature before electrophoresis. If needed, Fe^2+^ was added into the mixture to check its impact on the formation of VgrR-DNA complexes. After the reaction, 4 μl of DNA loading buffer (0.25% bromophenol, 80% glycerol) was added to stop the EMSA reaction and the samples were loaded into a 5% native PAGE gel. Electrophoresis was performed under 120 V for about 40 min using 0.5 × TBE buffer before autoradiography.

### Chromatin immunoprecipitation (ChIP-seq) and ChIP-qPCR

ChIP was performed according to a previous study [48]. Briefly, bacterial strains were grown in MMX or MMX plus 100 μM Fe^3+^ media until the OD_600 nm_ reached 0.4. Cells were collected by centrifugation, cross-linked with 1% formaldehyde and subsequently quenched by 0.5 M glycine for 10 min. Bacterial cells were collected by centrifugation, washed in 10 ml of cold TBS buffer (150 mM NaCl, 20 mM Tris-HCl pH 7.5) and resuspended in 1 ml lysis buffer (10 mM Tris pH 8.0, 20% sucrose, 50 mM NaCl, 10 mM EDTA, 10 mg/ml lysozyme). Immunoprecipitation (IP) buffer (50 mM HEPES-KOH pH 7.5, 150 mM NaCl, 1 mM EDTA, 1% Triton X 100, 0.1% sodium deoxycholate, 0.1% SDS, 1 mM phenylmethylsulfonyl fluoride) was added to the bacterial cell suspension and the cells were sonicated using a Diagenode Bioruptor (Diagenode, Liège, Belgium) to generate DNA fragments of about 150–300 bp. After centrifugation, the solution was pre-cleared with 20 μl of protein A sepharose at 4°C for 10 min on a slow rotator, and a 100-μl aliquot was retained as the loading control DNA (input sample). For the ChIP assays, 50 μl of protein A sepharose (50% slurry) and 2 μl of an anti-His_6_ antibody were added to an 800-μl aliquot of the DNA sample and the mixture was incubated at 4°C overnight with slow rotation. Next day, the beads were collected by centrifugation and washed with IP buffer and wash buffer [10 mM Tris-HCl pH 8.0, 250 mM LiCl, 1 mM EDTA, 0.5% Nonidet-P40 (equivalent to Triton X-100), 0.5% sodium deoxycholate]. The immunoprecipitated chromatin was removed from the beads by adding 100 μl of elution buffer (50 mM Tris pH 7.5, 10 mM EDTA, 1% SDS) and the solution was incubated for 10 min at 65°C. RNase A and proteinase K were used to remove RNA and protein, respectively. The DNA was purified using a PCR purification kit (Qiagen, Duesseldorf, Germany) after overnight 65°C crosslinking reversal. High-throughput sequencing was performed using the Illumina Highseq-2000 system (Illumina, San Diego, CA USA)by the Beijing Institute of Genomics genomic service.

The high-throughput sequencing reads were analyzed by Burrows–Wheeler aligner (BWA) method. The cleaned read were aligned to the genomic sequence of *X*. *campestris* pv. *campestris* 8004. Peak calling was conducted by MACS2 [53]. The consensus VgrR-binding motif analysis was completed using the MEME and FIMO tools in the MEME software suite [54].

ChIP-qPCR was performed to quantify the amount of VgrR-PtdvA binding *in vivo*, ChIP was carried out as above. Typically, 10 ng of immunoprecipitated DNA input was quantified by qPCR using Maxima SYBR Green (Fermentas) in a DNA Engine Option 2 System (Bio-Rad), according to the manufacturer’s instructions. The proportion of immunoprecipitated *tdvA* promoter fragment was calculated in comparison to the amount of input PCR product.

### DNase I footprinting assay

DNase I footprinting experiments were performed to determine the VgrR binding sites on the promoter region of *tdvA* (*XC1241*). DNA probes of P*tdvA* were labeled by [γ-^32^P]ATP. The VgrR-P*tdvA* Binding reactions were carried out in a binding buffer [10 mM Tris-HCl, pH 7.5, 50 mM KCl, 1 mM DTT, 5 mM MgCl_2_, 1 μl poly(dI·dC) (50 ng/μl) and 2.5% glycerol]. The labeled DNA probes were mixed with VgrR protein at 0.15–15 μM in a 50-μl reaction volume for 30 min. Subsequently, the reaction mixture subjected to 0.1 U DNase I (Promega) treatment for 5 min. The reactions were stopped by adding 50 μl DNase I stop solution (20 mM EGTA, pH 8.0). Digested DNA samples were purified by phenol-chloroform extraction and ethanol precipitation. Pellets containing DNA were air-dried and resuspended in formamide containing loading dye (10 mM Tris-HCl, pH 8.0, 20 mM EDTA, pH 8.0, 0.05% bromophenol blue, 0.05% xylene cyanol). Samples were heated at 95°C for 3 min before loading into an 8% polyacrylamide gel containing 7 M urea. The dried gel was exposed to a phosphor screen and scanned using a PhosphorImage system (GE Healthcare). The sequencing ladder was generated using an Accupower DNA sequencing kit (Bioneer, Daejeon, Korea) with the same [γ-^32^P]-end-labeled oligonucleotide primer.

### Microscale thermophoresis (MST) measurement

MST was used to quantify the VgrS sensor-Fe^3+^ binding and VgrR-P*tdvA* binding affinities. 10 μM purified VgrR, truncated VgrS, VgrS sensor or VgrS^E43A^ sensor were labeled using Protein Labeling Kit RED-NHS (Nano Temper Technologies GMBH, München, Germany). The labeled protein was displaced by a buffer containing 50 mM Tris-HCl (pH 7.4), 150 mM NaCl, 10 mM MgCl_2_ and 0.05% (V/V) Tween-20. Binding reactions were measured using a microscale thermophoresis instrument (Nano Temper Technologies GMBH) in the Monolith NT.115 standard treated capillaries. To quantify the affinity of VgrR-P*tdvA* binding, 5′-FAM-labeled oligonucleotide primer (50 bp) for P*tdvA* was synthesized by Invitrogen technology company (China) and annealed with unlabeled complementary primer to form double-stranded DNA (dsDNA). The labeled dsDNA was added to serially diluted protein reaction volumes (at an initial concentration of 40 μM), containing 50 mM Tris-HCl (pH 7.4), 150 mM NaCl, 10 mM MgCl_2_ and 0.05% (V/V) Tween-20. If necessary, VgrR was phosphorylated by soluble, truncated VgrS in the presence of 100 μM ATP; the labeled dsDNA was then added to the serially diluted protein reaction volumes. To determine the impact of Fe^2+^ on VgrR-P*tdvA* binding, the ferrous ion was serially diluted from 0.061 to 10 mM. The KD Fit function of the Nano Temper Analysis Software Version 1.5.41 was used to fit the curve and calculate the value of the dissociation constant (*K*_d_).

## Supporting Information

S1 Fig Mutations in *vgrR* and *vgrS* impact the bacterial virulence and growth. (A) Mutations in *vgrR* and *vgrS* caused virulence attenuation against host plant cabbage (*Brassica oleraceae* cv. Jingfeng No. 1). Eight-weeks old plants were inoculated by bacterial strains. 1 mM MgCl_2_ was inoculated as negative control. Virulence scale was estimated 10 days after inoculation. (B) semi-quantification of the virulence scales of bacterial strains. * indicate significant difference (P < 0.05, n = 12). (C and D) Addition of iron in media remarkably increased growth of *vgrR* and *vgrS* mutants. Bacterial strains were grown in 28°C under iron-depleted (MMX) and replete (MMX plus 100 μM Fe^3+^) conditions. Each data point was the average of 3 experiments. Vertical bars indicates standard deviations.
(PDF)

S2 Fig Soluble, truncated VgrS phosphorylates VgrR. Truncated VgrS with linker-DHp-CA domain (5 μM) was incubated with 100 μM ATP containing 10 μCi [γ-^32^P]ATP for 20 min, 15 μM VgrR was added into the reaction. The reaction was stopped by loading buffer before SDS-PAGE separation and autoradiography. The gel was stained by Coomassie brilliant blue to check the amount of proteins (lower panel). Each experiment was repeated three times.
(PDF)

S3 Fig Verification of the expression of genes identified by comparative proteomics. Semi-quantitative RT-PCR was used to compare the expression levels of genes between different bacterial strains under iron-deplete (MMX) and replete conditions (MMX + 100 μM Fe^3+^), respectively. cDNAs synthesized from total RNA of bacterial strains were used as template. Amplification of cDNA of 16S RNA was used as loading control.–RT: negative control, in which reverse transcriptase was absent when synthesizing cDNA. The experiment was repeated 3 times.
(PDF)

S4 Fig VgrR directly binds to a 50 bp region in the *tdvA* promoter. EMSA was used to determine the VgrR-DNA interaction. 50 bp DNA probe was chemically synthesized and labeled by [γ-^32^P]ATP. Each lane contains 4 fmol probe. Unlabeled probe was used as competitor. The experiment was repeated 3 times.
(PDF)

S5 Fig *tdvA* (*XC1241*) mutant has similar growth rate as wild-type strain in iron-replete medium. Bacterial strains were grown under 28°C in MMX plus 100 μM Fe^3+^ medium (iron-replete). Each data point is the average of 3 experiments. Bars indicate standard deviations.
(PDF)

S6 Fig VgrS sensor did not binds Fe^2+^. Microscale thermopheresis (MST) was used to determine the possible binding between VgrS sensor and Fe^2+^. 4 μM VgrS sensor was used in MST assay. The titration of Fe^2+^ is from 0.061 μM to 2 mM. Three independent experiments were conducted. Vertical bars represent standard deviation.
(PDF)

S1 Table Strains and plasmids used in this study. (PDF)

S2 Table Primers used in this study. (PDF)

S3 Table Identification of differently expressed proteins of the *vgrR* mutant and the wild-type strain grown in rich NYG medium (iron-replete). (PDF)

S4 Table Identification of differently expressed proteins of the *vgrR* mutant and the wild-type strain grown in MMX medium (iron-depleted). (PDF)

S5 Table Identification of differently expressed proteins of the *vgrR* mutant and the wild-type strain grown in MMX medium plus iron (iron replete). (PDF)

S6 Table ChIP-seq analysis identifies genes with promoter regions bound by VgrR in *X*. *campestris*. pv. *campestris* grown in MMX medium. (PDF)

S7 Table ChIP-seq analysis identifies genes with promoter regions bound by VgrR in *X*. *campestris*. pv. *campestris* grown in MMX supplemented with 100 μM Fe^3+^ medium. (PDF)

## References

[1] Andrews SC, Robinson AK, Rodriguez-Quinones F. Bacterial iron homeostasis. FEMS Microbiol Rev. 2003;27(2–3):215–237. 12829269 10.1016/S0168-6445(03)00055-X

[2] Cassat JE, Skaar EP. Iron in infection and immunity. Cell Host Microbe. 2013;13(5):509–519. 10.1016/j.chom.2013.04.010 23684303 PMC3676888

[3] Fischbach MA, Lin H, Liu DR, Walsh CT. How pathogenic bacteria evade mammalian sabotage in the battle for iron. Nat Chem Biol. 2006;2(3):132–138. 10.1038/nchembio771 16485005

[4] Hantke K. Iron and metal regulation in bacteria. Curr Opin Microbiol. 2001;4(2):172–177. 11282473 10.1016/s1369-5274(00)00184-3

[5] Skaar EP. The battle for iron between bacterial pathogens and their vertebrate hosts. PLoS Pathog. 2010;6(8):e1000949 10.1371/journal.ppat.1000949 20711357 PMC2920840

[6] Noinaj N, Guillier M, Barnard TJ, Buchanan SK. TonB-dependent transporters: regulation, structure, and function. Annu Rev Microbiol. 2010;64:43–60. 10.1146/annurev.micro.112408.134247 20420522 PMC3108441

[7] Troxell B, Hassan HM. Transcriptional regulation by Ferric Uptake Regulator (Fur) in pathogenic bacteria. Front Cell Infect Microbiol. 2013;3:59 10.3389/fcimb.2013.00059 24106689 PMC3788343

[8] Stock AM, Robinson VL, Goudreau PN. Two-component signal transduction. Annu Rev Biochem. 2000;69:183–215. 10.1146/annurev.biochem.69.1.183 10966457

[9] Wosten MM, Kox LF, Chamnongpol S, Soncini FC, Groisman EA. A signal transduction system that responds to extracellular iron. Cell. 2000;103(1):113–125. 11051552 10.1016/s0092-8674(00)00092-1

[10] Kreamer NN, Wilks JC, Marlow JJ, Coleman ML, Newman DK. BqsR/BqsS constitute a two-component system that senses extracellular Fe(II) in *Pseudomonas aeruginosa*. J Bacteriol. 2012;194(5):1195–1204. 10.1128/JB.05634-11 22194456 PMC3294787

[11] Hyytiainen H, Sjoblom S, Palomaki T, Tuikkala A, Tapio Palva E. The PmrA-PmrB two-component system responding to acidic pH and iron controls virulence in the plant pathogen *Erwinia carotovora* ssp. *carotovora*. Mol Microbiol. 2003;50(3):795–807. 14617142 10.1046/j.1365-2958.2003.03729.x

[12] Juarez-Verdayes MA, Gonzalez-Uribe PM, Peralta H, Rodriguez-Martinez S, Jan-Roblero J, Escamilla-Hernandez R, et al Detection of *hssS*, *hssR*, *hrtA*, and *hrtB* genes and their expression by hemin in *Staphylococcus epidermidis*. Can J Microbiol. 2012;58(9):1063–1072. 10.1139/w2012-086 22906238

[13] Stauff DL, Torres VJ, Skaar EP. Signaling and DNA-binding activities of the *Staphylococcus aureus* HssR-HssS two-component system required for heme sensing. J Biol Chem. 2007;282(36):26111–26121. 10.1074/jbc.M703797200 17635909

[14] Blanvillain S, Meyer D, Boulanger A, Lautier M, Guynet C, Denance N, et al Plant carbohydrate scavenging through TonB-dependent receptors: a feature shared by phytopathogenic and aquatic bacteria. PLoS One. 2007;2(2):e224 10.1371/journal.pone.0000224 17311090 PMC1790865

[15] Qian W, Jia Y, Ren SX, He YQ, Feng JX, Lu LF, et al Comparative and functional genomic analyses of the pathogenicity of phytopathogen *Xanthomonas campestris* pv. *campestris*. Genome Res. 2005;15(6):757–767. 10.1101/gr.3378705 15899963 PMC1142466

[16] Jittawuttipoka T, Sallabhan R, Vattanaviboon P, Fuangthong M, Mongkolsuk S. Mutations of ferric uptake regulator (*fur*) impair iron homeostasis, growth, oxidative stress survival, and virulence of *Xanthomonas campestris* pv. *campestris*. Arch Microbiol. 2010;192(5):331–339. 10.1007/s00203-010-0558-8 20237769

[17] Qian W, Han ZJ, Tao J, He C. Genome-scale mutagenesis and phenotypic characterization of two-component signal transduction systems in *Xanthomonas campestris* pv. *campestris* ATCC 33913. Mol Plant Microbe Interact. 2008;21(8):1128–1138. 10.1094/MPMI-21-8-1128 18616409

[18] Zhang SS, He YQ, Xu LM, Chen BW, Jiang BL, Liao J, et al A putative *colR*(*XC1049*)-*colS*(*XC1050*) two-component signal transduction system in *Xanthomonas campestris* positively regulates *hrpC* and *hrpE* operons and is involved in virulence, the hypersensitive response and tolerance to various stresses. Res Microbiol. 2008;159(7–8):569–578. 10.1016/j.resmic.2008.06.010 18694822

[19] Subramoni S, Pandey A, Vishnu Priya MR, Patel HK, Sonti RV. The ColRS system of *Xanthomonas oryzae* pv. *oryzae* is required for virulence and growth in iron-limiting conditions. Mol Plant Pathol. 2012;13(7):690–703. 10.1111/j.1364-3703.2011.00777.x 22257308 PMC6638901

[20] Yeowell HN, White JR. Iron requirement in the bactericidal mechanism of streptonigrin. Antimicrob Agents Chemother. 1982;22(6):961–968. 6218780 10.1128/aac.22.6.961 PMC185701

[21] Wilson TJ, Bertrand N, Tang JL, Feng JX, Pan MQ, Barber CE, et al The *rpfA* gene of *Xanthomonas campestris* pathovar *campestris*, which is involved in the regulation of pathogenicity factor production, encodes an aconitase. Mol Microbiol. 1998;28(5):961–970. 9663682 10.1046/j.1365-2958.1998.00852.x

[22] Hassett DJ, Schweizer HP, Ohman DE. *Pseudomonas aeruginosa sodA* and *sodB* mutants defective in manganese- and iron-cofactored superoxide dismutase activity demonstrate the importance of the iron-cofactored form in aerobic metabolism. J Bacteriol. 1995;177(22):6330–6337. 7592406 10.1128/jb.177.22.6330-6337.1995 PMC177481

[23] Dong YH, Zhang XF, An SW, Xu JL, Zhang LH. A novel two-component system BqsS-BqsR modulates quorum sensing-dependent biofilm decay in *Pseudomonas aeruginosa*. Commun Integr Biol. 2008;1(1):88–96. 19513205 10.4161/cib.1.1.6717 PMC2633808

[24] Gunn JS, Ryan SS, Van Velkinburgh JC, Ernst RK, Miller SI. Genetic and functional analysis of a PmrA-PmrB-regulated locus necessary for lipopolysaccharide modification, antimicrobial peptide resistance, and oral virulence of *Salmonella enterica* serovar *typhimurium*. Infect Immun. 2000;68(11):6139–6146. 11035717 10.1128/iai.68.11.6139-6146.2000 PMC97691

[25] Bogel G, Schrempf H, Ortiz de Orue Lucana D. The heme-binding protein HbpS regulates the activity of the *Streptomyces reticuli* iron-sensing histidine kinase SenS in a redox-dependent manner. Amino Acids. 2009;37(4):681–691. 10.1007/s00726-008-0188-5 18931968

[26] Stearman R, Yuan DS, Yamaguchi-Iwai Y, Klausner RD, Dancis A. A permease-oxidase complex involved in high-affinity iron uptake in yeast. Science. 1996;271(5255):1552–1557. 8599111 10.1126/science.271.5255.1552

[27] Loprasert S, Sallabhan R, Atichartpongkul S, Mongkolsuk S. Characterization of a ferric uptake regulator (*fur*) gene from *Xanthomonas campestris* pv. *phaseoli* with unusual primary structure, genome organization, and expression patterns. Gene. 1999;239(2):251–258. 10548726 10.1016/s0378-1119(99)00412-6

[28] Qian W, Han ZJ, He C. Two-component signal transduction systems of *Xanthomonas* spp.: a lesson from genomics. Mol Plant Microbe Interact. 2008;21(2):151–161. 10.1094/MPMI-21-2-0151 18184059

[29] An SQ, Allan JH, McCarthy Y, Febrer M, Dow JM, Ryan RP. The PAS domain-containing histidine kinase RpfS is a second sensor for the diffusible signal factor of *Xanthomonas campestris*. Mol Microbiol. 2014;92(3):586–597. 10.1111/mmi.12577 24617591 PMC4159695

[30] Barber CE, Tang JL, Feng JX, Pan MQ, Wilson TJ, Slater H, et al A novel regulatory system required for pathogenicity of *Xanthomonas campestris* is mediated by a small diffusible signal molecule. Mol Microbiol. 1997;24(3):555–566. 9179849 10.1046/j.1365-2958.1997.3721736.x

[31] Wang LH, He Y, Gao Y, Wu JE, Dong YH, He C, et al A bacterial cell-cell communication signal with cross-kingdom structural analogues. Mol Microbiol. 2004;51(3):903–912. 14731288 10.1046/j.1365-2958.2003.03883.x

[32] Liang H, Deng X, Bosscher M, Ji Q, Jensen MP, He C. Engineering bacterial two-component system PmrA/PmrB to sense lanthanide ions. J Am Chem Soc. 2013;135(6):2037–2039. 10.1021/ja312032c 23350529

[33] Perez JC, Groisman EA. Acid pH activation of the PmrA/PmrB two-component regulatory system of *Salmonella enterica*. Mol Microbiol. 2007;63(1):283–293. 10.1111/j.1365-2958.2006.05512.x 17229213 PMC1804205

[34] Gunn JS. The Salmonella PmrAB regulon: lipopolysaccharide modifications, antimicrobial peptide resistance and more. Trends Microbiol. 2008;16(6):284–290. 10.1016/j.tim.2008.03.007 18467098

[35] Chen HD, Groisman EA. The biology of the PmrA/PmrB two-component system: the major regulator of lipopolysaccharide modifications. Annu Rev Microbiol. 2013;67:83–112. 10.1146/annurev-micro-092412-155751 23799815 PMC8381567

[36] Hindt MN, Guerinot ML. Getting a sense for signals: regulation of the plant iron deficiency response. Biochim Biophys Acta. 2012;1823(9):1521–1530. 10.1016/j.bbamcr.2012.03.010 22483849 PMC4008143

[37] Kobayashi T, Nishizawa NK. Iron uptake, translocation, and regulation in higher plants. Annu Rev Plant Biol. 2012;63:131–152. 10.1146/annurev-arplant-042811-105522 22404471

[38] Innan H, Kondrashov F. The evolution of gene duplications: classifying and distinguishing between models. Nat Rev Genet. 2010;11(2):97–108. 10.1038/nrg2689 20051986

[39] Capra EJ, Laub MT. Evolution of two-component signal transduction systems. Annu Rev Microbiol. 2012;66:325–347. 10.1146/annurev-micro-092611-150039 22746333 PMC4097194

[40] Laub MT, Goulian M. Specificity in two-component signal transduction pathways. Annu Rev Genet. 2007;41:121–145. 10.1146/annurev.genet.41.042007.170548 18076326

[41] Ainsaar K, Mumm K, Ilves H, Horak R. The ColRS signal transduction system responds to the excess of external zinc, iron, manganese, and cadmium. BMC Microbiol. 2014;14:162 10.1186/1471-2180-14-162 24946800 PMC4074579

[42] Steele KH, O'Connor LH, Burpo N, Kohler K, Johnston JW. Characterization of a ferrous iron-responsive two-component system in nontypeable *Haemophilus influenzae*. J Bacteriol. 2012;194(22):6162–6173. 10.1128/JB.01465-12 22961857 PMC3486404

[43] Deng CY, Deng AH, Sun ST, Wang L, Wu J, Wu Y, et al The periplasmic PDZ domain-containing protein Prc modulates full virulence, envelops stress responses, and directly interacts with dipeptidyl peptidase of *Xanthomonas oryzae* pv. *oryzae*. Mol Plant Microbe Interact. 2014;27(2):101–112. 10.1094/MPMI-08-13-0234-R 24200074

[44] Moeck GS, Coulton JW. TonB-dependent iron acquisition: mechanisms of siderophore-mediated active transport. Mol Microbiol. 1998;28(4):675–681. 9643536 10.1046/j.1365-2958.1998.00817.x

[45] Koebnik R. TonB-dependent trans-envelope signalling: the exception or the rule? Trends Microbiol. 2005;13(8):343–347. 10.1016/j.tim.2005.06.005 15993072

[46] Kadner RJ, Heller KJ. Mutual inhibition of cobalamin and siderophore uptake systems suggests their competition for TonB function. J Bacteriol. 1995;177(17):4829–4835. 7665457 10.1128/jb.177.17.4829-4835.1995 PMC177254

[47] Higgs PI, Larsen RA, Postle K. Quantification of known components of the *Escherichia coli* TonB energy transduction system: TonB, ExbB, ExbD and FepA. Mol Microbiol. 2002;44(1):271–281. 11967085 10.1046/j.1365-2958.2002.02880.x

[48] Wang FF, Deng CY, Cai Z, Wang T, Wang L, Wang XZ, et al A three-component signalling system fine-tunes expression kinetics of HPPK responsible for folate synthesis by positive feedback loop during stress response of *Xanthomonas campestris*. Environ Microbiol. 2014;16(7):2126–2144. 10.1111/1462-2920.12293 24119200

[49] Rai R, Javvadi S, Chatterjee S. Cell-cell signalling promotes ferric iron uptake in *Xanthomonas oryzae* pv. *oryzicola* that contribute to its virulence and growth inside rice. Mol Microbiol. 2015;96(4):708–727. 10.1111/mmi.12965 25656587

[50] Desrosiers DC, Sun YC, Zaidi AA, Eggers CH, Cox DL, Radolf JD. The general transition metal (Tro) and Zn^2+^ (Znu) transporters in *Treponema pallidum*: analysis of metal specificities and expression profiles. Mol Microbiol. 2007;65(1):137–152. 10.1111/j.1365-2958.2007.05771.x 17581125

[51] Le Moual H, Quang T, Koshland DE, Jr. Conformational changes in the cytoplasmic domain of the *Escherichia coli* aspartate receptor upon adaptive methylation. Biochemistry. 1998;37(42):14852–14859. 10.1021/bi980343y 9778360

[52] Yuan Z, Wang L, Sun S, Wu Y, Qian W. Genetic and proteomic analyses of a *Xanthomonas campestris* pv. *campestris purC* mutant deficient in purine biosynthesis and virulence. J Genet Genomics. 2013;40(9):473–487. 10.1016/j.jgg.2013.05.003 24053949

[53] Liu T. Use model-based Analysis of ChIP-Seq (MACS) to analyze short reads generated by sequencing protein-DNA interactions in embryonic stem cells. Methods Mol Biol. 2014;1150:81–95. 10.1007/978-1-4939-0512-6_4 24743991

[54] Bailey TL, Johnson J, Grant CE, Noble WS. The MEME Suite. Nucleic Acids Res. 2015;43(W1):W39–49. 10.1093/nar/gkv416 25953851 PMC4489269

