# Supplementary material for: Two-Component Signaling System VgrRS Directly Senses Extracytoplasmic and Intracellular Iron to Control Bacterial Adaptation under Iron Depleted Stress
Source: PLoS Pathog. 2016 Dec 30;12(12):e1006133. doi: 10.1371/journal.ppat.1006133 (PMC5231390; doi:10.1371/journal.ppat.1006133)
Supplement: S1 Fig — (A) Mutations in vgrR and vgrS caused virulence attenuation against host plant cabbage (Brassica oleraceae cv. Jingfeng No. 1). Eight-weeks old plants were inoculated by bacterial strains. 1 mM MgCl2 was inoculated as negative control. Virulence scale was estimated 10 days after inoculation. (B) semi-quantification of the virulence scales of bacterial strains. * indicate significant difference (P < 0.05, n = 12). (C and D) Addition of iron in media remarkably increased growth of vgrR and vgrS mutants. Bacterial strains were grown in 28°C under iron-depleted (MMX) and replete (MMX plus 100 μM Fe3+) conditions. Each data point was the average of 3 experiments. Vertical bars indicates standard deviations. (PDF) [file ppat.1006133.s001.pdf]

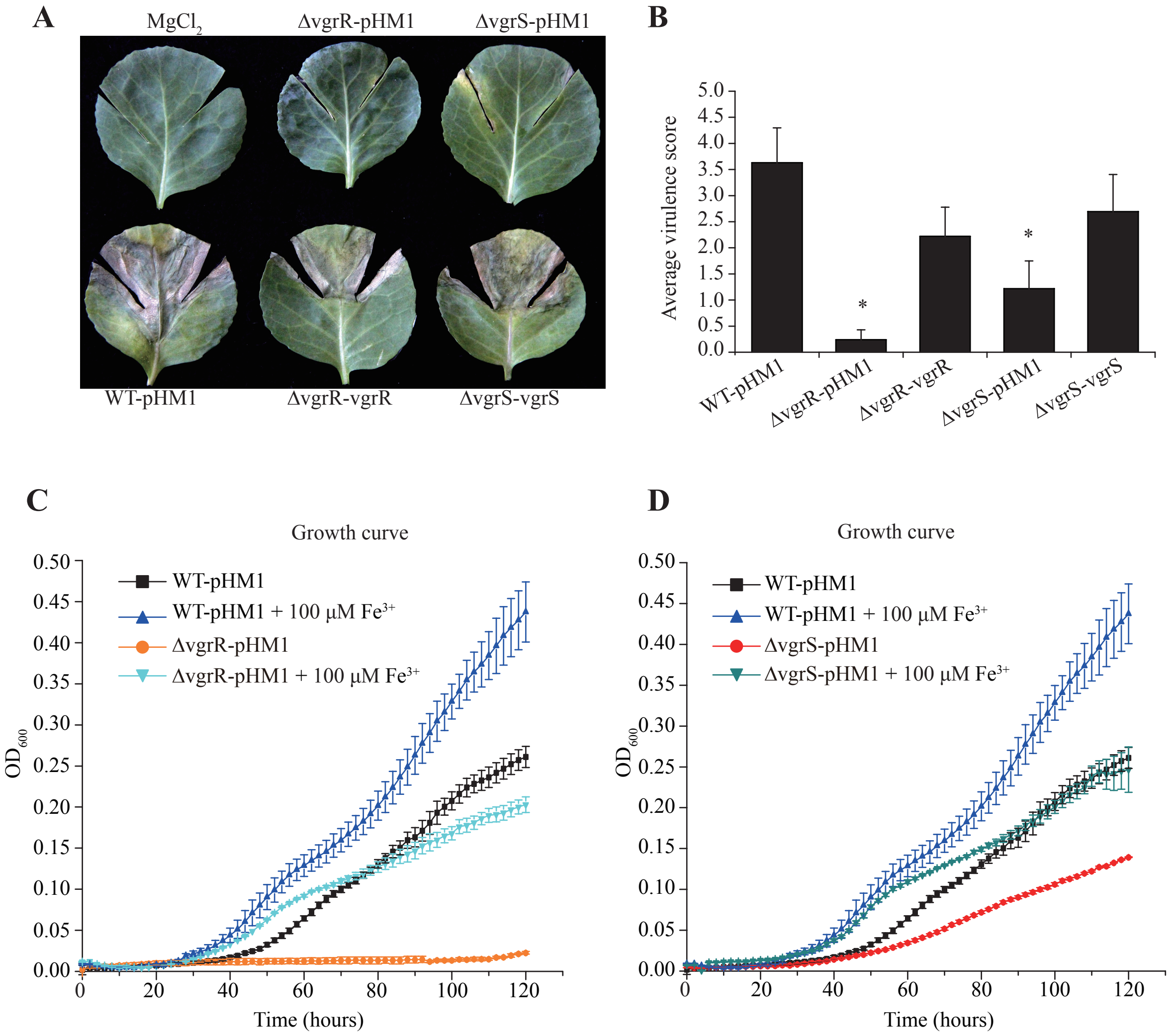

S1 Fig. Mutations in *vgrR* and *vgrS* impact the bacterial virulence and growth. (A) Mutations in *vgrR* and *vgrS* caused virulence attenuation against host plant cabbage (*Brassica oleraceae* cv. Jingfeng No. 1). Eight-weeks old plants were inoculated by bacterial strains. 1 mM $MgCl_2$ was inoculated as negative control. Virulence scale was estimated 10 days after inoculation. (B) Semi-quantification of the virulence scales of bacterial strains. * indicate significant difference ($P < 0.05$, $n = 12$). (C and D) Addition of iron in media remarkably increased growth of *vgrR* and *vgrS* mutants. Bacterial strains were grown in 28 °C under iron-depleted (MMX) and replete (MMX plus 100 μM Fe^{3+}) conditions. Each data point was the average of 3 experiments. Vertical bars indicates standard deviations.
