# Supplementary material for: Two-Component Signaling System VgrRS Directly Senses Extracytoplasmic and Intracellular Iron to Control Bacterial Adaptation under Iron Depleted Stress
Source: PLoS Pathog. 2016 Dec 30;12(12):e1006133. doi: 10.1371/journal.ppat.1006133 (PMC5231390; doi:10.1371/journal.ppat.1006133)
Supplement: S2 Fig — Truncated VgrS with linker-DHp-CA domain (5 μM) was incubated with 100 μM ATP containing 10 μCi [γ-32P]ATP for 20 min, 15 μM VgrR was added into the reaction. The reaction was stopped by loading buffer before SDS-PAGE separation and autoradiography. The gel was stained by Coomassie brilliant blue to check the amount of proteins (lower panel). Each experiment was repeated three times. (PDF) [file ppat.1006133.s002.pdf]

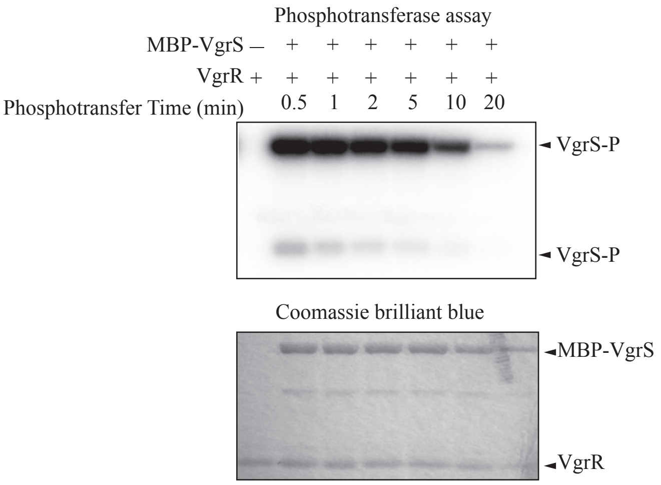

S2 Fig. Soluble, truncated VgrS phosphorylates VgrR. Truncated VgrS with linker-DHp-CA domain (5 μM) was incubated with 100 μM ATP containing 10 μCi [γ-^{32}P]ATP for 20 min, 15 μM VgrR was added into the reaction. The reaction was stopped by loading buffer before SDS-PAGE separation and autoradiography. The gel was stained by Coomassie brilliant blue to check the amount of proteins (lower panel). Each experiment was repeated three times.
