# Supplementary material for: Two-Component Signaling System VgrRS Directly Senses Extracytoplasmic and Intracellular Iron to Control Bacterial Adaptation under Iron Depleted Stress
Source: PLoS Pathog. 2016 Dec 30;12(12):e1006133. doi: 10.1371/journal.ppat.1006133 (PMC5231390; doi:10.1371/journal.ppat.1006133)
Supplement: S3 Fig — Semi-quantitative RT-PCR was used to compare the expression levels of genes between different bacterial strains under iron-deplete (MMX) and replete conditions (MMX + 100 μM Fe3+), respectively. cDNAs synthesized from total RNA of bacterial strains were used as template. Amplification of cDNA of 16S RNA was used as loading control.–RT: negative control, in which reverse transcriptase was absent when synthesizing cDNA. The experiment was repeated 3 times. (PDF) [file ppat.1006133.s003.pdf]

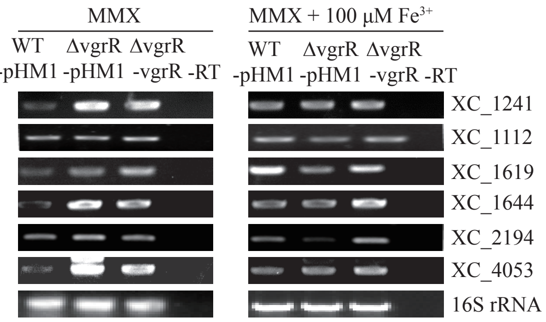

S3 Fig. Verification of the expression of genes identified by comparative proteomics. Semi-quantitative RT-PCR was used to compare the expression levels of genes between different bacterial strains under iron-deplete (MMX) and replete conditions (MMX + 100 μM Fe^{3+}), respectively. cDNAs synthesized from total RNA of bacterial strains were used as template. Amplification of cDNA of 16S RNA was used as loading control. –RT: negative control, in which reverse transcriptase was absent when synthesizing cDNA. The experiment was repeated 3 times.
