# Supplementary material for: Two-Component Signaling System VgrRS Directly Senses Extracytoplasmic and Intracellular Iron to Control Bacterial Adaptation under Iron Depleted Stress
Source: PLoS Pathog. 2016 Dec 30;12(12):e1006133. doi: 10.1371/journal.ppat.1006133 (PMC5231390; doi:10.1371/journal.ppat.1006133)
Supplement: S4 Fig — EMSA was used to determine the VgrR-DNA interaction. 50 bp DNA probe was chemically synthesized and labeled by [γ-32P]ATP. Each lane contains 4 fmol probe. Unlabeled probe was used as competitor. The experiment was repeated 3 times. (PDF) [file ppat.1006133.s004.pdf]

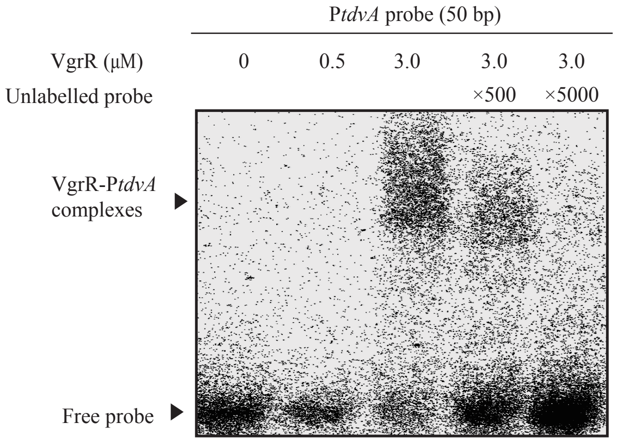

S4 Fig. VgrR directly binds to a 50 bp region in the *tdvA* promoter. EMSA was used to determine the VgrR-DNA interaction. 50 bp DNA probe was chemically synthesized and labeled by [γ-^{32}P]ATP. Each lane contains 4 fmol probe. Unlabeled probe was used as competitor. The experiment was repeated 3 times.
