# Supplementary material for: Two-Component Signaling System VgrRS Directly Senses Extracytoplasmic and Intracellular Iron to Control Bacterial Adaptation under Iron Depleted Stress
Source: PLoS Pathog. 2016 Dec 30;12(12):e1006133. doi: 10.1371/journal.ppat.1006133 (PMC5231390; doi:10.1371/journal.ppat.1006133)
Supplement: S5 Fig — Bacterial strains were grown under 28°C in MMX plus 100 μM Fe3+ medium (iron-replete). Each data point is the average of 3 experiments. Bars indicate standard deviations. (PDF) [file ppat.1006133.s005.pdf]

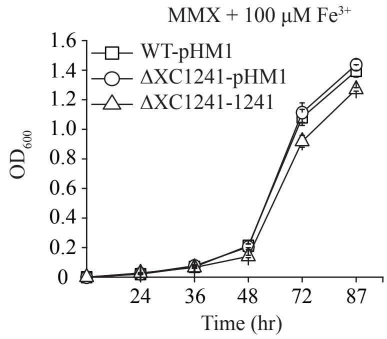

S5 Fig. *tdvA* (*XC1241*) mutant has similar growth rate as wild-type strain in iron-replete medium. Bacterial strains were grown under 28 °C in MMX plus 100 μM Fe^{3+} medium (iron-replete). Each data point is the average of 3 experiments. Bars indicate standard deviations.
