# Supplementary material for: Two-Component Signaling System VgrRS Directly Senses Extracytoplasmic and Intracellular Iron to Control Bacterial Adaptation under Iron Depleted Stress
Source: PLoS Pathog. 2016 Dec 30;12(12):e1006133. doi: 10.1371/journal.ppat.1006133 (PMC5231390; doi:10.1371/journal.ppat.1006133)
Supplement: S6 Fig — Microscale thermopheresis (MST) was used to determine the possible binding between VgrS sensor and Fe2+. 4 μM VgrS sensor was used in MST assay. The titration of Fe2+ is from 0.061 μM to 2 mM. Three independent experiments were conducted. Vertical bars represent standard deviation. (PDF) [file ppat.1006133.s006.pdf]

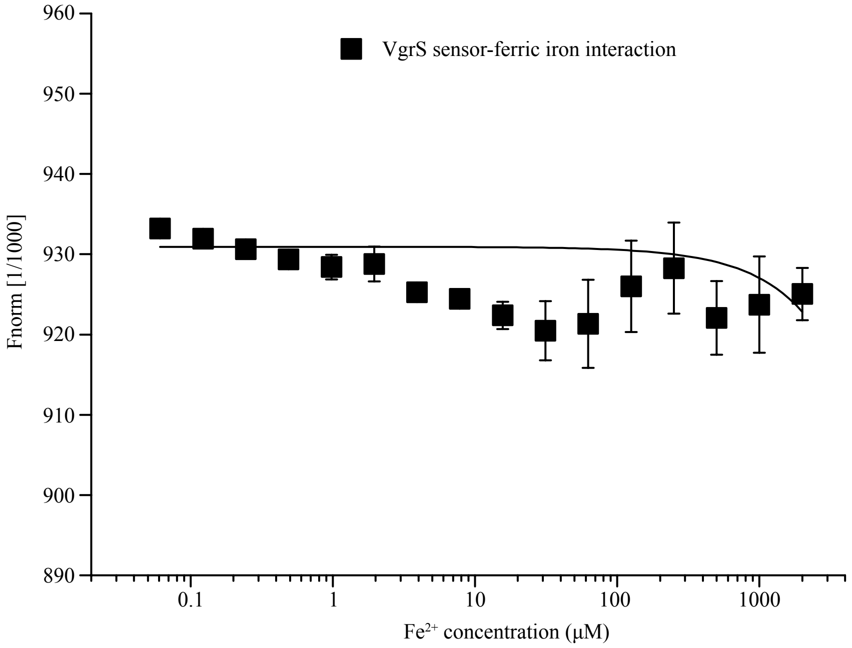

S6 Fig. VgrS sensor did not binds Fe^{2+}. Microscale thermopheresis (MST) was used to determine the possible binding between VgrS sensor and Fe^{2+}. 4 µM VgrS sensor was used in MST assay. The titration of Fe^{2+} is from 0.061 µM to 2 mM. Three independent experiments were conducted. Vertical bars represent standard deviation.
