# Supplementary material for: Two-Component Signaling System VgrRS Directly Senses Extracytoplasmic and Intracellular Iron to Control Bacterial Adaptation under Iron Depleted Stress
Source: PLoS Pathog. 2016 Dec 30;12(12):e1006133. doi: 10.1371/journal.ppat.1006133 (PMC5231390; doi:10.1371/journal.ppat.1006133)
Supplement: S1 Table — (PDF) [file ppat.1006133.s007.pdf]

## S1 Table. Strains and plasmids used in the study

| Strains and plasmid | Genotype or description* | Resource or reference |
|---|---|---|
| **Strains** | | |
| ***Escherichia coli*** | | |
| DH5α | *fhuA2 Δ(argF-lacZ)U169 phoA glnV44 Φ80 Δ(lacZ)M15 gyrA96 recA1 relA1 endA1 thi-1 hsdR17* | Lab collection |
| TB1 | *ara Δ*（*lac proAB*） *rpsL* （Φ80 *lacΔZM15*） *hsdR* | Biolabs |
| BL21(DE3) | *E.coli B F*⁻ *ompT hsdSB (rB*⁻ *mB*⁻*) gal dcm met* (DE3) | Novagen |
| ***Xanthomonas campestris* pv. *campestri*** | | |
| WT | WT, Wild-type of *X. campestris* pv. *campestris* 8004, $Rif^r$ | Lab collection |
| WT-pHM1 | WT-pHM1, WT strain containing a blank pHM1 vector, $Sp^r$ | Lab collection |
| M0120 | ΔvgrR, marker-exchange mutant of *vgrR*, replacement *vgrR* with a tetracycline resistance genes, $Tc^r$ | This work |
| M0121 | ΔvgrR-pHM1, ΔvgrR strain containing a blank pHM1 vector, $Sp^r$ | This work |
| M0122 | ΔvgrS, *vgrS* in-frame deletion mutant, $Rif^r$ | This work |
| M0123 | ΔvgrS-pHM1, ΔvgrS strain containing a blank pHM1 vector, $Sp^r$ | |
| M0124 | ΔvgrR–vgrR, genetic complementary strain of *vgrR* mutant which contains a vector of pHM1::vgrR, $Sp^r$ | This work |
| M0125 | ΔvgrS–vgrS, genetic complementary strain of *vgrS* mutant which contains a vector of pHM1::vgrS, $Sp^r$ | This work |
| M0126 | P1-GUS, WT strain containing a recombinant vector of pHM2::P1-GUS transcriptional fusion, $Sp^r$ | This work |
| M0127 | P2-GUS, WT strain containing a recombinant vector of pHM2::P2-GUS transcriptional fusion, $Sp^r$ | This work |
| M0128 | P1+P2-GUS, WT strain containing a recombinant vector of pHM2::P1+P2-GUS transcriptional fusion, $Sp^r$ | This work |
| M0128 | ΔXC1241, insertional mutant of *tdvA* (*XC1241*), $Kan^r$ | This work |
| M0129 | ΔXC1241-pHM1, ΔXC1241 strain containing a blank pHM1 vector, $Sp^r$ | This work |
| M0130 | ΔXC1241–XC1241, genetic complementary strain of *XC1241* mutant which contains a vector of pHM1::*tdvA*, $Sp^r$ | This work |
| M0131 | WT-P*tdvA*-GUS, WT strain containing a recombinant vector pHM2::P*tdvA-GUS*, $Sp^r$ | This work |
| M0132 | ΔvgrR-P*tdvA*-GUS, ΔvgrR strain containing a recombinant vector pHM2::P*tdvA-GUS*, $Sp^r$ | This work |
| M0133 | ΔvgrS-P*tdvA*-GUS, ΔvgrS strain containing a recombinant vector pHM2::P*tdvA-GUS*, $Sp^r$ | This work |
| M0134 | VgrR-$His_6$, WT strain containing a recombinant vector pHM1::*vgrR-his*$_6$, $Sp^r$ | This work |
| M0135 | $VgrR^{D51A}$-$His_6$, WT strain containing a recombinant vector pHM1::*vgrR*$^{D51A}$-*his*$_6$, $Sp^r$ | This work |
| M0136 | ΔvgrR-$vgrR^{D51A}$, ΔvgrR derivate containing a recombinant | This work |

| | | |
|---|---|---|
| | vector pHM1::*vgrR*$^{D51A}$, Sp$^r$ | |
| M0137 | ΔvgrS-vgrS$^{H186A}$, ΔvgrS derivate containing a recombinant vector pHM1::*vgrS*$^{H186A}$, Sp$^r$ | This work |
| M0138 | ΔvgrS-vgrS$^{\Delta sensor}$, ΔvgrS derivate containing a recombinant vector pHM1::*vgrS*$^{\Delta sensor}$, Sp$^r$ | This work |
| **Plasmids** | | |
| pHM1 | Broad-host-range *cos* IncW derivative of pRI40, Sp$^r$ | (Innes et al., 1988) |
| pHM2 | Complementary vector with no promoter before MCS, Sp$^r$ | Lab collection |
| pK18mob | Suicide plasmid for *Xcc*, Kan$^r$ | (Schafer et al., 1994) |
| pK18mobSacB | Suicide plasmid for *Xcc*, Kan$^r$ | (Schafer et al., 1994) |
| pET30a | Protein expression vector, Kan$^r$ | Novagen |
| pMal-p2X | Protein expression vector, Amp$^r$ | Biolabs |
| pET-vgrR | pET30a::*vgrR*, Kan$^{r,}$ expressing full-length VgrR | This work |
| pET-vgrR$^{D51A}$ | pET30a::*vgrR*$^{D51A}$, Kan$^{r,}$ expressing full-length VgrR$^{D51A}$ | This work |
| pET-vgrS | pET30a::*vgrS*, Kan$^r$, expressing full-length VgrS | This work |
| pET-vgrSa$^{H186A}$ | pET30a::*vgrS*$^{H186A}$, Kan$^r$, expressing full-length VgrSH$^{186A}$ | This work |
| pET-vgrS$^{cyto}$ | pET30a::*vgrS*$^{cyto}$, Kan$^r$, expressing truncated, cytosolic part of VgrS without input region. | This work |
| pMal-vgrS$^{cyto}$ | pMal-p2X:: *vgrS*$^{cyto}$, Amp$^r$, expressing truncated, cytosolic part of VgrS without input region (MBP-VgrS). | This work |
| pET-vgrS sensor | pET30a::*vgrS* sensor, Kan$^r$, expressing sensor region of VgrS | This work |
| pET-vgrS$^{E43A}$ | pET30a::*vgrS*$^{E43A}$ sensor, Kan$^r$, expressing sensor region of VgrS with a substitution of E43A | This work |
| pET-vgrS$^{P44A}$ | pET30a::*vgrS*$^{P44A}$ sensor, Kan$^r$, expressing sensor region of VgrS with a substitution of P44A | This work |
| pET-vgrS$^{Q45A}$ | pET30a::*vgrS*$^{Q45A}$ sensor, Kan$^r$, expressing sensor region of VgrS with a substitution of Q45A | This work |
| pET-vgrSs$^{E46A}$ | pET30a::*vgrS*$^{E46A}$ sensor, Kan$^r$, expressing sensor region of VgrS with a substitution of E46A | This work |
| pMBP-VgrS | pMal-p2X::*vgrSa*, Amp$^r$ | This work |
| pK18-vgrR | pK18mobSacB::*vgrR*-*tet*, recombinant suicide vector for replacing *vgrR* with tet, Tc$^r$ | This work |
| pK18-vgrS | pK18mobSacB::*vgrS*, recombinant suicide vector for constructing *vgrS* in-frame deletion mutant, kan$^r$ | This work |
| pK18-1241 | pK18mob::*tdvA*, recombinant suicide vector for constructing *tdvA* (XC1241) insertion inactivation mutant, kan$^r$ | This work |
| pHM1-vgrR | pHM1::*vgrR*, recombinant vector for genetic complementation of *vgrR* mutant, Sp$^r$ | This work |
| pHM1-vgrR$^{D51A}$ | pHM1::*vgrR*$^{D51A}$, recombinant vector for genetic complementation of *vgrR* mutant, containing a point mutation, Sp$^r$ | This work |

| | | |
|---|---|---|
| pHM1-vgrS$^{H186A}$ | pHM1::*vgrS*$^{H186A}$, recombinant vector for genetic complementation of *vgrS* mutant, containing a point mutation, Sp$^{r}$ | This work |
| pHM1-vgrR-his$_6$ | pHM1::*vgrR*-his$_6$, Sp$^{r}$, recombinant vector for ChIP-seq | This work |
| pHM1-vgrR$^{D51A}$-his$_6$ | pHM1::*vgrR*$^{D51A}$-his$_6$, Sp$^{r}$, recombinant vector for ChIP-seq, containing a point mutation | This work |
| pHM1-vgrS | pHM1::*vgrS*, recombinant vector for genetic complementation of *vgrS* mutant, Sp$^{r}$ | This work |
| pHM1-1241 | pHM1::*XC1241*, recombinant vector for genetic complementation of *tdvA* (*XC1241*) mutant, Sp$^{r}$ | This work |
| pHM2-P1-GUS | pHM2::P1-*gusA*, Sp$^{r}$, for quantify promoter activity of P1 by GUS reporter | This work |
| pHM2-P2-GUS | pHM2::P2-*gusA*, Sp$^{r}$, for quantify promoter activity of P2 by GUS reporter | This work |
| pHM2-P1+P2-GUS | pHM2::P1+P2-*gusA*, Sp$^{r}$, for quantify promoter activity of P1 + P2 by GUS reporter | This work |
| pHM2-PtdvA-GUS | pHM2::P*tdvA*-*gusA*, Sp$^{r}$, for quantify promoter activity of P*tdvA* by GUS reporter | This work |

* Kan$^{r,}$ kanamysin resistance; Sp$^{r,}$, Specinomycin resistance, Tet$^{r}$, tetracycline resistance, Rif$^{r}$, rifamysin resistance.

**References**

Innes, R.W., Hirose, M.A., and Kuempel, P.L. (1988). Induction of nitrogen-fixing nodules on clover requires only 32 kilobase pairs of DNA from the *Rhizobium trifolii* symbiosis plasmid. J Bacteriol *170*, 3793-3802.

Schafer, A., Tauch, A., Jager, W., Kalinowski, J., Thierbach, G., and Puhler, A. (1994). Small mobilizable multi-purpose cloning vectors derived from the *Escherichia coli* plasmids pK18 and pK19: selection of defined deletions in the chromosome of *Corynebacterium glutamicum*. Gene *145*, 69-73.
