# Supplementary material for: Two-Component Signaling System VgrRS Directly Senses Extracytoplasmic and Intracellular Iron to Control Bacterial Adaptation under Iron Depleted Stress
Source: PLoS Pathog. 2016 Dec 30;12(12):e1006133. doi: 10.1371/journal.ppat.1006133 (PMC5231390; doi:10.1371/journal.ppat.1006133)
Supplement: S2 Table — (PDF) [file ppat.1006133.s008.pdf]

S2 Table. Primers used in this study

Primer name	Sequence (5′-3′)	Description
vgrR-up-F<br>vgrR-up-R<br>vgrR-down-F<br>vgrR-down-R<br>Tet-F<br>Tet-R	GCAGAATTCAACACGCATGACGCAACAG<br>ACTGGATCCTCGCACTGGAACCTCGA<br>ATAGGATCCATGATCCAGACCCGTCACGG<br>CGCAAGCTTGTACGAATACGCGTT<br>ATCGGATCCTCAATCGTCACCCTTTCTCGGTCC<br>ACGTCTAGATCAGCGATCGGCTCGTTGCC	Used for construction of marker-exchange mutant of *vgrR* by pK18mobsacB
vRcomp-F<br>vRcomp-R	GGCAAGCTTGTGCGAATTCTAGTAATCGAAGAC<br>TGAGGTACCTCAGGCATCGGGCGAG	Used for construction genetic complementary vector of *vgrR*.
vgrS-up-F<br>vgrS-up-R<br>vgrS-down-F<br>vgrS-down-R	GCAgaattCTGATCAAGCCGTTCGCGTT<br>CGTggtacCACGTCCTCCACCAGCTGGTTT<br>CGTggtaccCTGGCGTTCCATCGCTAA<br>ATCaagcttCGCTTCCGTCGCAACATG	Used for construction of in-frame deletion mutant of *vgrS* by pK18mobsacB
vScomp-F<br>vScomp-R	CAAgtcgacATGAACCGCAATATCGACGC<br>TCAgagctcTTAGCGATGGAACGCCAGTGT	Used for construction genetic complementary vector of *vgrS*.
1241-sF<br>1241-sR	GAATTCGAGCTAATGAATTGCAAGTCCA<br>AAGCTTCAACGTTGACGCCAGCTTC	Used for construction of insertion inactivation mutant of *tdvA*
M13F<br>1241-sFA<br>M19R<br>1241-sRA	GTAAAACGACGGCCAGT<br>CCGATTTCACGTCTTTCT<br>GCGGATAACAATTTCACAC<br>CCAGCCATCACCGAATC	Used to verify the correction of *tdvA* insertional mutant
1241-cF<br>1241-cR	CTGCAGTTGGAGAGAGAGCTAATGAATTG<br>GAATTCTCAGAAGCGCTGACGGTAC	Used for construction genetic complementary vector of *tdvA*.
vgrR-RT-F02<br>vgrR-RT-R01	GCACCATCATCCAGCATCT<br>CGCTGTTGTCTTCGATTACTAG	Used for RT-PCR assay to check if *vgrR* and *XC_1048* are in an operon
vgrS-RT-F03<br>vgrS-RT-R03	GACCTATACGGCTGGCGC<br>CTGCTCGTGCATGCGCTCT	Used for RT-PCR assay to check if *vgrS* and *XC_1051* are in an operon
vgrR-F<br>vgrS-R	GGTGGACAAGCCGTTCGACGTA<br>ACGCAGCGGATCGGAATAGAAC	Used for RT-PCR assay to check if *vgrR* and *vgrS* are in an operon
vgrS-RT-F04<br>vgrS-RT-R04	GCGTACGACATGACCCAGA<br>GAGTAATCGTCCAGCGCTT	Used for RT-PCR of *vgrS* gene, positive control
vgrR-his-F<br>vgrR-his-R	CATATGGTGCGAATTCTAGTAATCGAAGAC<br>AAGCTTTCAGGCATCGGGCGAG	Used for construction of pET30a::VgrR vector
vgrSa-his-F	GCTCATATGATGAACCGCAATATCGACGC	

vgrSa-his-R	Used for construction of pET30a::VgrS vector, full length<br>AAACTCGAGGCGATGGAACGCCAGTGTT	
vgrSc-his-F<br>vgrSc-his-R	CATATGTCCGCATCCAAGGTCATGC<br>AAGCTTGCGATGGAACGCCAGTGTT	Used for construction of pET30a::VgrS vector, truncated protein
vgrSs-his-F<br>vgrSs-his-R	CATATGAACCGCAATATCGACGC<br>CTCGAGGGTGCGCTTGAGCTGGA	Used for construction of pET30a::VgrS sensor vector
MBP-VgrS-F<br>MBP-VgrS-R	GGATCCATGCCTGAGAGCTCCAGCGGA<br>AAGCTTTTAGCGATGGAACGCCAGTGT	Used for construction of vector to express MBP fusioned VgrS, truncated protein
vgrR-sp3	ACGGTCTTCCAGGTAGTCAC	Used for primer extension assay
D51A-vF<br>D51A-vR	GACGCCATCGTGCTCGCGCTCAACCTGCC<br>CGCGAGCACGATGGCGTCGAACTCGTGCAC	Used for construction of pint-mutated vectors of pHM1::VgrR-His$_6$ and pET30a::VgrR-His$_6$
H186A-vF<br>H186A-vR	CAATGCCGACGTCAGCGCCGAACTGCGTAC<br>GGCGCTGACGTCGGCATTGAATTCGCGGTC	Used for construction of pint-mutated vectors of pHM1::VgrS-His$_6$ and pET30a::VgrS-His$_6$
S-E43A-F<br>S-E43A-R	CGAAGCGCTGCGTCGCGCGCAGCCCGAGT<br>CGCGCGACGCAGCGCTTCGAACTTGTCGC	Used for construction of point mutated vector to express recombinant VgrS with substitution in ExxE motif within its sensor region
S-Q44A-F<br>S-Q44A-R	AGCGCTGCGTCGCGAGGCGCCCGAGTGGTA<br>CGCCTCGCGACGCAGCGCTTCGAACTTGTC	
S-P45A-F<br>S-P45A-R	CTGCGTCGCGAGCAGGCGGAGTGGTACCAG<br>CGCCTGCTCGCGACGCAGCGCTTCGAACTT	
S-E46A-F<br>S-E46A-R	CGTCGCGAGCAGCCCGCGTGGTACCAGCTA<br>CGCGGGCTGCTCGCGACGCAGCGCTTCGAA	
vgrR-TF<br>vgrR-TR	CTGCAACTTTCCAGCCCCATT<br>TCCATGCCCGGCAGGTTGA	Used for generating PCR products of *vgrR*, which act as a template in primer extension assay
P1-F<br>P1-R	GCAGTCGACAACACGCATGACGCAACAG<br>ACTGGATCCCAGGTAGTCACCCAGATT	Used for construction of promoter-GUS transcriptional fusion constructs.
P2-F<br>P2-R	GCAGTCGACAACACGCATGACGCAACAG<br>ACTGGATCCGAGTCACTGACGCGGCGG	
P3-F<br>P3-R	GCAGTCGACCTCAAAACAATCGAGGTTC<br>ACTGGATCCCAGGTAGTCACCCAGATT	
GUS-2F<br>GUS-2R	AGAGGATCCCCGGGTGGTCA<br>GGTACCGATCTAGTAACATAGATGACACCG	Used for amplify GUS gene that wused in constructing transcriptional fusion

		vectors
vRhis-F<br>vRhis-R	GGCAAGCTTGTGCGAATTCTAGTAATCGAAGAC<br>TGAGGTACCTCAGTGGTGGTGGTGGTGGTGGTGG TGGTGGTGGGCATCGGGCGAG	Used for construction of a VgrR-His_6 fusion, inserted into pHM1 vector
1241box-1-F<br>1241box-1-R	CCCGCCCGATTTCACGTCTTGGG<br>CCCAAGACGTGAAATCGGGCGGG	Used for amplify DNA probe of P*tdvA* in EMSA assay
1241box-2-F<br>1241box-2-R	CCCTCTTCTTTTTCATTAAGGG<br>CCCTTAATGAAAAAGAAGAGGG	
1241box-3-F<br>1241box-3-R	CCCCGGGACTTTAATTCCTGGGG<br>CCCCAGGAATTAAAGTCCCGGGG	
Motif-50-F<br>Motif-50-R	GCCCGATTTCACGTCTTTCTTCTTTTTCATTAACG GGACTTTAATTCCTG<br>CAGGAATTAAAGTCCCGTTAATGAAAAAGAAGA AAGACGTGAAATCGGGC	Used for construct of a DNA probe of P*tdvA* in EMSA. Containing VgrR-binding motif
1241-EF<br>1241-ER	GCACTGCAGGCGTTTTACGTTGTG<br>GTACCATGGCTCTCTCTCCAAGTGATC	Used for EMSA as P*tdvA* probe, also used for ChIP-qPCR amplification
P1241-F<br>P1241-R	GTCGACCGAGAGCAACAGCGACG<br>CCCGGGGTGATCTTGGGTACTGCCAA	Used for construction of P*tdvA*-GUS fusion
1112-F<br>1112-R	CGGTGGTTTCATGGACAAGG<br>CGGATAGATCGAGGGCACATT	Used for semi-quantitative RT-PCR to check the expression levels of corresponding genes
1241-F<br>1241-R	TATGAATACCGCAAGGAAAGC<br>GCATGGATCGGCATAGGTT	
1619-F<br>1619-R	TCGGGCAAGAACCAGAAT<br>AGAAGGTATCGGTCCAATCA	
1644-F<br>1644-R	GCAACTACAACGGCGGTTACAC<br>GCCCTTGAGCACGTCTTCC	
2194-F<br>2194-R	CGCCATTTCCACCAAGCA<br>CCATTGGGCACGGTCTTTT	
4053-F<br>4053-R	CAAGCCACTGGACAACATCACC<br>CGGACACGCCCAACGAAT	
16S-F<br>16S-R	GCCTAACACATGCAAGTCGAACGGC<br>AATATTCCCCACTGCTGCCTCCCG	
