# Supplementary material for: Two-Component Signaling System VgrRS Directly Senses Extracytoplasmic and Intracellular Iron to Control Bacterial Adaptation under Iron Depleted Stress
Source: PLoS Pathog. 2016 Dec 30;12(12):e1006133. doi: 10.1371/journal.ppat.1006133 (PMC5231390; doi:10.1371/journal.ppat.1006133)
Supplement: S3 Table — (PDF) [file ppat.1006133.s009.pdf]

S3 Table. Identification of differently expressed proteins of the *vgrR* mutant and the wild-type strain grown in rich NYG medium (iron-replete)

Spot ID	Protein Name	NCBI acc. no.[a]	Mascot Score	Sequence Coverage	Theoretical MW(Da)/pI	Wild type/Mutant
**Transport Protein**						
N40	Outer membrane protein	XC_0017	116	26	47114/5.18	+1.65
N1	MopB outer membrane protein	XC_3300	109	29	39566/4.59	+1.84
N2	FyuA TonB-dependent receptor	XC_0806	70	21	89119/4.97	+1.75
N3	FhuA TonB-dependent receptor	XC_1451	187	38	86053/4.95	+3.52
N32	OmpA-related protein	XC_4224	205	42	110800/5.11	-1.96
N33	Azi Div preprotein translocase subunit SecA	XC_3501	60	24	102596/5.47	-1000000
**Signal Transduction**						
N4	Two-component system regulatory protein	XC_1049	159	67	25063/5.27	+1000000
N8	DnaK supressor	XC_1861	50	41	19064/5.22	-1.63
N51	Methanol dehydrogenase regulatory protein	XC_0936	118	51	37533/5.59	+1000000
**Transcription and translation**						
N44	ProS prolyl-tRNA synthetase	XC_3596	86	20	61758/5.49	+1.51
N5	Tuf2 elongation factor Tu	XC_3354	50	30	43386/5.45	+1.58
N34	IleS isoleucyl-tRNA synthetase	XC_3087	92	22	105167/5.59	-1.58
N35	LysS lysyl-tRNA synthetase	XC_2336	145	44	56859/5.51	-1000000
N36	Rho DNA-dependent helicase II Transcription termination factor Rho	XC_3850	460	57	81730/5.82	-1.50
N37	DNA topoisomerase IV subunit B	XC_2536	172	37	70336/6.11	-1.73
N23	RpoB DNA-directed RNA polymerase subunit beta	XC_3347	237	29	155092/5.36	-1000000

N28	GltX glutamyl-tRNA synthetase	XC_1429	53	34	52065/5.39	-1.73
**Cell Division**						
N7	MinD septum site-determining protein	XC_3122	44	31	29091/5.22	+1.60
**Protein Maintenance and Folding**						
N6	PpiD peptidyl-prolyl cis-trans isomerase	XC_3261	204	38	71318/5.65	+1.50
N38	Tig trigger factor	XC_3266	165	48	48185/5.23	+1.51
**Degradive Enzymes**						
N10	Alpha-xylosidase	XC_2480	177	28	106538/6.06	+1.73
N11	Aminopeptidase	XC_0643	183	50	59259/6.49	+1.79
N39	Ribonuclease E	XC_2098	66	15	125816/5.33	-1.50
N14	Ribokinase	XC_3468	164	73	32525/5.56	+1.56
N18	Methylmalonate-semialdehyde dehydrogenase	XC_2981	128	31	53004/5.46	+1.61
**Fatty acid metabolish**						
N41	FabH 3-oxoacyl-(acyl carrier protein) synthase III	XC_3229	81	24	34899/4.96	+1.71
N16	AccD acetyl-CoA carboxylase subunit beta	XC_1578	48	36	32392/6.11	-1.98
**Carbonhydrate metabolism**						
N42	Beta-hexosaminidase	XC_2958	83	19	34666/5.29	+1.96
N43	Fructose-bisphosphate aldolase	XC_0979	98	23	36538/4.98	-1.62
N45	Transaldolase B	XC_3400	115	32	34801/4.91	-3.61
N9	Fbp fructose-1,6-bisphosphatase	XC_0098	131	49	37002/5.60	-1.82
N12	Aldose 1-epimerase	XC_3050	71	44	39664/5.56	+1.62
N15	PpsA phosphoenolpyruvate synthase	XC_1952	238	41	86515/5.12	-1.78
N17	Bifunctional aconitate hydratase 2/2-methylisocitrate dehydratase	XC_2326	91	29	93456/5.16	-1.62
N27	Ppa inorganic pyrophosphatase	XC_0851	82	48	19871/4.86	-1.55
N29	HemE uroporphyrinogen decarboxylase	XC_1265	95	32	38679/5.87	-1.78
N49	HemE uroporphyrinogen decarboxylase	XC_1265	62	13	38679/5.87	+1.54

**Nucleotides metabolism**						
N46	Adenylosuccinate synthetase	XC_3193	58	11	46477/5.56	-1.96
N24	Phosphoribosylaminoimidazole carboxylase ATPase subunit	XC_1614	99	42	39934/5.28	-1.57
N25	Gmk guanylate kinase	XC_0958	158	57	22781/5.82	-1.50
**Amino acid metabolism**						
N47	Family II 2-keto-3-deoxy-D-arabino-heptulosonate 7-phosphate synthase	XC_3313	106	26	53535/5.85	+1000000
N19	Branched-chain amino acid aminotransferase	XC_3380	137	61	39127/5.46	-1.72
N20	Tdh L-threonine 3-dehydrogenase	XC_3290	102	43	37533/6.06	-1.61
N21	Beta-alanine synthetase	XC_1919	290	50	32722/5.43	+1.56
N22	PepA leucyl aminopeptidase	XC_3585	89	25	51901/5.11	+4.07
**Energy metabolism**						
N48	NADH dehydrogenase subunit G	XC_1595	320	38	79889/6.15	-1.55
N26	AtpA ATP synthase F0F1 subunit alpha	XC_3680	192	49	55404/5.32	-1.53
**Detoxification**						
N13	Chloroacetaldehyde dehydrogenase	XC_0104	99	38	55823/5.34	+3.12
**Hypothetical Protein**						
N50	Hypothetical protein	XC_0732	113	25	43408/4.81	+1.82
N30	Hypothetical protein	XC_4310	167	32	89998/6.19	+1.87
N31	Hypothetical protein	XC_3269	77	181	14648/5.29	-1.82

[a] Names and codes of the identified proteins are according to the genomic annotation of *X. campestris* pv. *campestris* 8004.
