# Supplementary material for: Two-Component Signaling System VgrRS Directly Senses Extracytoplasmic and Intracellular Iron to Control Bacterial Adaptation under Iron Depleted Stress
Source: PLoS Pathog. 2016 Dec 30;12(12):e1006133. doi: 10.1371/journal.ppat.1006133 (PMC5231390; doi:10.1371/journal.ppat.1006133)
Supplement: S4 Table — (PDF) [file ppat.1006133.s010.pdf]

**S4 Table. Identification of differently expressed proteins of the *vgrR* mutant and the wild-type strain grown in MMX medium (iron-depleted)**

| Spot ID | Protein Name[a] | NCBI acc. no. | Mascot Score | Sequence Coverage | Theoretical MW(Da)/pI | Wild type/Mutant |
|---|---|---|---|---|---|---|
| **Transport Protein** | | | | | | |
| M1 | TonB-dependent receptor | XC_1241 | 412 | 45 | 101375/4.69 | -1.81 |
| M7 | TonB-dependent receptor | XC_1241 | 412 | 45 | 101375/4.69 | -2.26 |
| M8 | TonB-dependent receptor | XC_1241 | 412 | 45 | 101375/4.69 | -2.30 |
| M50 | TonB-dependent receptor | XC_1241 | 412 | 45 | 101375/4.69 | -1000000 |
| M2 | TonB-dependent receptor | XC_0806 | 563 | 63 | 89119/4.97 | +3.85 |
| M3 | TonB-dependent receptor | XC_2194 | 420 | 57 | 95083/5.02 | +1.51 |
| M4 | TonB-dependent receptor | XC_1644 | 223 | 33 | 102529/5.47 | +1.55 |
| M52 | TonB-dependent receptor | XC_4053 | 163 | 19 | 108228/4.90 | +1000000 |
| M53 | Outer membrane protein | XC_0017 | 300 | 43 | 47114/5.18 | +1.98 |
| M55 | Outer membrane protein | XC_3300 | 54 | 30 | 39566/4.59 | -7.34 |
| M24 | Outer membrane lipoprotein Blc | XC_3960 | 221 | 71 | 21056/5.49 | -1000000 |
| M31 | Ferric enterobactin receptor | XC_1112 | 103 | 27 | 80504/5.57 | -1000000 |
| M51 | Oar protein | XC_1619 | 73 | 9 | 117851/4.95 | -1000000 |
| M33 | Multiphosphoryl transfer protein | XC_1744 | 281 | 40 | 89092/5.28 | +1.52 |
| **Signal Transduction** | | | | | | |
| M11 | Two-component system regulatory protein | XC_1049 | 188 | 67 | 25063/5.27 | +10000000 |
| M45 | RegA two-component system regulatory protein | XC_2457 | 233 | 64 | 20995/5.60 | +1.60 |
| M54 | Response regulator | XC_0850 | 249 | 35 | 39637/4.94 | -1.96 |
| M57 | PhS histidine kinase/response regulator hybrid protein | XC_0635 | 91 | 39 | 35120/4.78 | +1.55 |
| M46 | DNA-binding related protein | XC_1163 | 279 | 67 | 20414/5.71 | +1.51 |

| | | | | | | |
|---|---|---|---|---|---|---|
| M9 | Obg GTPase ObgE | XC_3091 | 165 | 50 | 37731/5.17 | -1.79 |
| **Transcription and translation** | | | | | | |
| M34 | GTP-binding elongation factor protein | XC_3309 | 292 | 52 | 62469/5.15 | -1.58 |
| **Cell division** | | | | | | |
| M6 | FtsZ cell division protein FtsZ | XC_3505 | 321 | 57 | 42478/4.78 | -1.65 |
| **Oxidation-Reduction process** | | | | | | |
| M13 | Oxidoreductase | XC_3170 | 199 | 33 | 32119/4.87 | +2.97 |
| M14 | Oxidoreductase | XC_3170 | 199 | 33 | 32119/4.87 | +2.11 |
| M15 | Oxidoreductase | XC_3766 | 473 | 72 | 30466/5.06 | +4.13 |
| M41 | Oxidoreductase | XC_3740 | 555 | 73 | 35987/5.62 | +1.55 |
| **Fatty acid metabolism** | | | | | | |
| M40 | Enoyl-CoA hydratase | XC_2978 | 121 | 37 | 40497/5.91 | +1.66 |
| **Carbonhydrate metabolism** | | | | | | |
| M12 | Thioredoxin | XC_1495 | 163 | 52 | 31286/4.60 | -1.65 |
| M35 | Phosphomannose isomerase / GDP-mannose Pyrophosphorylase | XC_3609 | 216 | 49 | 51044/5.52 | +1.73 |
| M38 | Dihydrolipoamide succinyltransferase | XC_2750 | 230 | 41 | 42388/5.88 | +1.69 |
| **Amino acid metabolism** | | | | | | |
| M19 | MtnD acireductone dioxygenase | XC_2370 | 162 | 49 | 21446/5.25 | +1.52 |
| **Nucleotide metabolism** | | | | | | |
| M16 | Inosine-uridine preferring nucleoside hydrolase | XC_0946 | 132 | 33 | 33546/5.35 | +1.73 |
| M32 | Pnp polynucleotide phosphorylase/polyadenylase | XC_1609 | 120 | 27 | 75738/5.54 | +1000000 |
| **Energy metabolism** | | | | | | |
| M43 | Short chain dehydrogenase | XC_2192 | 225 | 42 | 30873/6.20 | +8.82 |
| M39 | Zn-dependent alcohol dehydrogenase | XC_3774 | 172 | 55 | 42221/5.62 | +2.11 |
| **Detoxification** | | | | | | |

| | | | | | | |
|---|---|---|---|---|---|---|
| M20 | SodA superoxide dismutase [Mn] | XC_1837 | 381 | 75 | 22707/5.45 | +2.57 |
| M18 | NonF-related protein | XC_0804 | 242 | 74 | 24214/5.22 | +1.64 |
| **Pathogenicity** | | | | | | |
| M26 | AtsE protein | XC_0082 | 165 | 68 | 14495/5.12 | +1.66 |
| M29 | Osmotically inducible protein | XC_1368 | 198 | 53 | 15476/5.60 | +1000000 |
| **Pili and flagella assembly** | | | | | | |
| M58 | PilM fimbrial assembly membrane protein | XC_0941 | 323 | 58 | 38462/4.75 | -1.73 |
| **Degradive Enzymes** | | | | | | |
| M22 | Protease | XC_1350 | 324 | 81 | 19235/4.88 | +1000000 |
| M30 | Ribonuclease E | XC_2098 | 165 | 36 | 125816/5.33 | -1.73 |
| M36 | GlgE alpha-amylase | XC_0141 | 227 | 44 | 114924/5.73 | +2.47 |
| M37 | Beta-mannosidase | XC_1218 | 142 | 29 | 100191/6.30 | +1.53 |
| **Hypothetical Protein** | | | | | | |
| M5 | Hypothetical protein | XC_3768 | 160 | 58 | 44333/4.97 | +1000000 |
| M17 | Hypothetical protein | XC_3971 | 232 | 61 | 29871/5.50 | +3.22 |
| M42 | Hypothetical protein | XC_3971 | 232 | 61 | 29871/5.50 | +2.77 |
| M27 | Hypothetical protein | XC_3883 | 117 | 53 | 14088/5.53 | +1000000 |
| M28 | Hypothetical protein | XC_2921 | 101 | 41 | 16465/5.82 | +8.82 |
| M47 | Hypothetical protein | XC_2921 | 101 | 41 | 16465/5.82 | +11.59 |
| M10 | Hypothetical protein | XC_3754 | 409 | 77 | 32399/4.70 | +7.65 |
| M59 | Hypothetical protein | XC_3291 | 49 | 31 | 25938/4.32 | -2.82 |
| M25 | Hypothetical protein | XC_3753 | 601 | 79 | 18405/5.04 | +2.10 |

[a] Names and codes of the identified proteins are according to the genomic annotation of *X. campestris* pv. *campestris* 8004.
