# Supplementary material for: Two-Component Signaling System VgrRS Directly Senses Extracytoplasmic and Intracellular Iron to Control Bacterial Adaptation under Iron Depleted Stress
Source: PLoS Pathog. 2016 Dec 30;12(12):e1006133. doi: 10.1371/journal.ppat.1006133 (PMC5231390; doi:10.1371/journal.ppat.1006133)
Supplement: S5 Table — (PDF) [file ppat.1006133.s011.pdf]

S5 Table. Identification of differently expressed proteins of the *vgrR* mutant and the wild-type strain grown in MMX medium plus iron (iron replete)

Spot ID	Protein Name[a]	NCBI acc. no.	Mascot Score	Sequence Coverage	Theoretical MW(Da)/pI	Wild type/ Mutant
**Transport Protein**						
F1	TonB-dependent receptor	XC_1241	560	43	101375/4.69	+2.90
F2	Outer membrane protein	XC_0017	59	16	47114/5.18	+1.87
F27	BamA outer membrane antigen	XC_2873	720	52	90687/5.33	+1000000
F32	Ferric enterobactin receptor	XC_1113	711	69	86199/5.15	+1.54
F20	Polyphosphate-selective porin O	XC_0819	212	55	42950/6.32	-1.82
F29	Polyphosphate-selective porin O	XC_0819	212	55	42950/6.32	-3.08
**Signal Transduction**						
F4	Two-component system sensor protein	XC_3068	215	33	33845/4.82	+3.41
F6	Two-component system regulatory protein	XC_1049	510	67	25063/5.27	+1000000
**Transcription and translation**						
F3	RplY 50S ribosomal protein L25	XC_3357	100	33	23179/5.01	+1.91
F26	TrpS tryptophanyl-tRNA synthetase	XC_4011	232	34	47217/5.74	+1000000
F12	RpoB RNA polymerase beta subunit	XC_3347	119	12	155053/5.38	+1.51
**Degradive enzymes**						
F18	PepQ proline dipeptidase	XC_0966	149	36	48505/5.68	-1.74
F33	Periplasmic protease	XC_2972	550	59	53938/7.77	-1.64
F8	PepA aminopeptidase A/I	XC_3585	172	30	51901/5.11	+1.63
F19	Cellulose	XC_1727	253	51	63016/6.07	+1.76
**Fatty acid metabolism**						
F23	3-oxoacyl-[ACP] synthase II	XC_3225	115	40	43160/5.68	+1.76

**Carbonhydrate metabolism**						
F28	LptD organic solvent tolerance precursor	XC_3437	692	58	92121/5.32	-1.59
F15	RTS beta protein	XC_4159	203	53	48222/5.21	-2.57
F14	2-deoxy-D-gluconate 3-dehydrogenase	XC_0161	557	64	26227/5.27	+1.62
F30	phosphoglucomutase; phosphomannomutase	XC_3608	129	42	49317/5.29	-1.66
**Amino acid metabolism**						
F7	Beta-alanine synthetase	XC_1919	176	44	32722/5.43	+1.73
F17	ArgG argininosuccinate synthase	XC_1871	620	65	43515/5.66	-1000000
F24	Homocysteine synthase	XC_1090	439	56	45646/5.67	+1.62
F21	Branched-chain amino acid aminotransferase	XC_3380	271	65	39127/5.46	+1.68
**Nucleotide metabolism**						
F9	PurH purine biosynthesis protein	XC_0510	161	45	55907/5.57	+1.72
F10	Pnp polynucleotide phosphorylase	XC_1609	326	46	75738/5.54	-1.83
F22	Pnp polynucleotide phosphorylase	XC_1609	326	46	75738/5.54	-1.71
**Detoxification**						
F16	NonF-related protein	XC_0804	76	46	24214/5.22	-1000000
**Hypothetical Protein**						
F5	Conserved hypothetical protein	XC_0025	122	68	15176/5.26	+2.97
F31	Conserved hypothetical protein	XC_0263	386	63	23402/6.23	+2.22

a Names and codes of the identified proteins are according to the genomic annotation of X. campestris pv. campestris 8004 (Qian et al., 2005)
