# Supplementary material for: Two-Component Signaling System VgrRS Directly Senses Extracytoplasmic and Intracellular Iron to Control Bacterial Adaptation under Iron Depleted Stress
Source: PLoS Pathog. 2016 Dec 30;12(12):e1006133. doi: 10.1371/journal.ppat.1006133 (PMC5231390; doi:10.1371/journal.ppat.1006133)
Supplement: S6 Table — (PDF) [file ppat.1006133.s012.pdf]

S6 Table. ChIP-seq analysis identifies genes with promoter regions bound by VgrR in *X. campestris*. pv. *campestris* grown in MMX medium

Code[a]	Gene function	Strand	Seq. peak Z-score	Peak start:peak end	Peak position	Direction
**Amino Acid Metabolism**						
XC_2568	serine protease	+	3.17102	3101057:3101644	3101528	ui
XC_2854	2,3,4,5-tetrahydropyridine-2,6-carboxylate N-succinyltransferase	-	2.99961	3433782:3433988	3433886	u
XC_0100	aromatic-amino-acid transaminase	-	3.16281	119196:119545	119316	u
XC_2806	asparagine synthase	-	4.28516	3376809:3377258	3377160	i
XC_0394	aspartyl/asparaginyl beta-hydroxylase	-	3.81535	457147:457616	457236	u
XC_0982	cysteine synthase	-	3.28515	1179244:1179495	1179412	ui
XC_3655	cysteine synthase	-	4.25257	4338272:4339122	4338701	u
XC_4041	shikimate 5-dehydrogenase	-	3.00527	4755711:4756082	4755790	di
XC_0293	serine-pyruvate aminotransferase	-	3.79324	348643:349738	348780	u
XC_1268	aroB 3-dehydroquinate synthase	-	2.44719	1557519:1557710	1557610	u
XC_2760	prephenate dehydrogenase	-	2.55841	3316905:3317229	3317006	ui
XC_2349	dihydrodipicolinate reductase	-	2.6568	2841772:2841893	2841798	u
XC_2388	homoserine kinase	-	2.39969	2880521:2880692	2880608	u
XC_2380	histidinol dehydrogenase	-	1.97117	2872929:2873104	2873091	u
XC_2378	imidazole glycerol-phosphate dehydratase/histidinol phosphatase	-	2.47465	2870607:2870770	2870703	u
**Biosynthesis of Small Molecules**						
XC_3088	bifunctional riboflavin kinase/FMN adenylyltransferase	-	3.91899	3701296:3701551	3701402	u
XC_0395	biotin synthesis protein	-	4.1451	458078:458966	458492	ui
XC_2553	biotin synthesis protein	-	4.02805	3088504:3088785	3088603	u
XC_1101	cobalamin synthase	+	3.33587	1327060:1328148	1328052	ui
XC_3887	dihydroneopterin aldolase	-	3.52469	4592757:4593090	4592993	u

XC_0301	gamma-glutamyltranspeptidase	-	4.33398	357710:358391	358256	ui
XC_0575	malonate decarboxylase subunit gamma	+	2.8686	687091:687520	687253	ui
XC_1169	pyrroloquinoline quinone biosynthesis protein PqqE	-	2.31235	1416268:1416390	1416338	u
XC_0397	biotin biosynthesis protein	-	3.10162	460623:461110	460723	ui
XC_3646	dithiobiotin synthetase	-	3.48255	4329064:4329226	4329161	u
XC_2174	uroporphyrin-III C-methyltransferase	-	3.51383	2620313:2620471	2620402	u
XC_2467	pantoate--beta-alanine ligase	-	2.48539	2985388:2985496	2985413	u
XC_3369	thioredoxin	-	2.79396	4019379:4019911	4019540	ui
XC_3165	thioredoxin reductase	-	2.93663	3793591:3793782	3793685	u
XC_4025	pantothenate kinase	-	4.00259	4738848:4739946	4739823	di
	**Fatty acid Metabolism**					
XC_1821	acetoacetyl-CoA reductase	-	2.79373	2214833:2215024	2214923	u
XC_0516	CDP-diacylglycerol--glycerol-3-phosphate 3-phosphatidyltransferase	+	2.83147	608585:609924	608998	ui
XC_4096	fatty acyl CoA synthetase	-	2.56449	4826736:4826838	4826792	u
XC_4117	cardiolipin synthase	-	4.97062	4852283:4853494	4853328	ui
	**Nucleotide Metabolism**					
XC_0670	cytosine deaminase	-	3.41497	804477:804640	804543	u
XC_0946	inosine-uridine preferring nucleoside hydrolase	-	2.75948	1136677:1136981	1136745	u
XC_1463	phosphodiesterase-nucleotide pyrophosphatase	-	2.6568	1765029:1765557	1765292	u
XC_0553	gluconolactonase	-	2.96421	663419:664592	664149	ui
	**Cell Structures and Division**					
XC_3941	phosphomannomutase	-	2.90435	4654212:4655082	4654333	ui
XC_3445	prolipoprotein diacylglyceryl transferase	-	2.77675	4095647:4095960	4095883	u
XC_0504	transmembrane protein	-	3.48505	592876:593061	592965	u

XC_4184	twin-arginine translocation protein TatA	-	3.43494	4940360:4940822	4940432	u
XC_1091	outer membrane receptor for transport of vitamin B	+	2.69567	1317012:1317409	1317134	ui
XC_3877	N-acetylmuramoyl-L-alanine amidase	-	3.38925	4581457:4582139	4582042	ui
XC_3512	phospho-N-acetylmuramoyl-pentapeptide-transferase	-	3.5334	4173526:4174391	4174162	ui
XC_3514	UDP-N-acetylmuramoylalanyl-D-glutamate--2,6-diaminopimelate ligase	-	3.31714	4175991:4176874	4176061	ui
XC_0517	acyltransferase	+	2.83147	608585:609924	608998	ui
XC_3504	UDP-3-O-[3-hydroxymyristoyl] N-acetylglucosamine deacetylase	-	4.32453	4163640:4163826	4163726	u
XC_3221	type IV fimbriae assembly protein	-	3.04544	3867009:3867302	3867153	u
XC_3934	chromosome partitioning protein	+	2.00597	4647454:4647739	4647510	ui
XC_1937	methyl-accepting chemotaxis protein	-	3.25672	2339540:2339719	2339637	u
	**Transport Protein**					
XC_3847	ABC transporter ATP-binding protein	-	3.55486	4544990:4545452	4545098	u
XC_3745	amino acid transporter	-	3.10672	4436038:4436202	4436099	u
XC_3785	ferrichrome-iron receptor	-	2.63575	4479909:4480043	4479958	u
XC_4249	ferrichrome-iron receptor 3	-	3.19827	5038890:5039164	5038977	u
XC_2355	ferrous iron transport protein	-	3.08531	2846911:2847390	2846999	u
XC_1104	iron transporter	-	2.96595	1332661:1333382	1333236	ui
XC_0570	iron utilization protein	-	3.73291	683273:683591	683498	u
XC_0460	monovalent cation/H+ antiporter subunit A	-	3.06135	547857:548052	547903	u
XC_4257	outer membrane efflux protein	+	4.10726	5048617:5049089	5048761	ui
XC_1783	polar amino acid transporter	-	2.31399	2170380:2170565	2170437	u
XC_1813	(Fe-S)-binding protein	-	2.5711	2205415:2205830	2205630	ui
XC_1122	TonB-dependent receptor	-	2.96305	1363532:1363736	1363585	u
XC_4141	TonB-dependent receptor	-	3.22711	4882614:4883158	4882964	u
XC_1241	TonB-dependent receptor	+	3.25945	1518206:1518353	1518281	u
XC_1462	chloride channel	-	4.11375	1763530:1764182	1763620	ui
XC_2839	outer membrane efflux protein	-	2.6568	3415346:3415487	3415412	u

XC_3459	permease	+	3.38732	4108547:4108942	4108737	ui
XC_0640	sulfate permease	+	37.89331	767442:768475	768312	ui
XC_4182	sec-independent protein translocase	-	1.8835	4939501:4939693	4939629	u
	**Central Intermediary**					
XC_3359	4-diphosphocytidyl-2-C-methyl-D-erythritol kinase	-	3.14283	4009126:4009426	4009241	u
XC_3537	6,7-dimethyl-8-ribityllumazine synthase	-	3.03168	4201302:4201497	4201439	u
XC_3830	tropinone reductase	-	3.44212	4527336:4527733	4527456	u
XC_1334	sulfite reductase	-	3.00228	1621826:1622021	1621920	u
	**Degradive Enzymes**					
XC_0378	beta-ketoadipyl CoA thiolase	+	2.49145	439600:439963	439637	ui
XC_2978	enoyl-CoA hydratase	-	3.77094	3565951:3566138	3566046	u
XC_0374	phenoxybenzoate dioxygenase subunit beta	-	2.66328	436738:436933	436829	u
XC_3868	HAD superfamily hydrolase	-	3.04859	4574496:4574911	4574843	u
XC_2654	N-formimino-L-glutamate deiminase	-	3.77094	3192522:3192921	3192680	u
XC_0165	phhA; phenylalanine 4-monooxygenase	-	2.27133	207069:207188	207125	u
	**Energy Metabolism**					
XC_1743	1-phosphofructokinase	-	3.85664	2110172:2110316	2110260	u
XC_3684	ATP synthase F0F1 subunit A	-	2.51238	4371778:4371880	4371826	u
XC_0441	branched-chain alpha-keto acid dehydrogenase subunit E2	-	3.57534	524267:524741	524623	i
XC_1885	cytochrome D ubiquinol oxidase subunit I	-	4.88508	2281618:2281987	2281712	u
XC_3691	dihydrolipoamide acetyltransferase	-	4.14863	4379751:4379975	4379880	u
XC_3689	dihydrolipoamide dehydrogenase	-	3.44997	4377363:4377866	4377794	u
XC_4120	FldA protein	+	3.11274	4853939:4854459	4854025	ui
XC_3287	phosphoglycerate mutase	-	2.80924	3936410:3936580	3936457	u
XC_3896	protoheme IX farnesyltransferase	-	2.36054	4602997:4603157	4603088	u

XC_2571	cytochrome c-type biogenesis protein CcmE	+	3.06285	3103588:3103862	3103782	ui
XC_3979	mercuric reductase	-	3.87182	4696300:4697192	4696701	ui
XC_2326	bifunctional aconitate hydratase 2/2-methylisocitrate dehydratase	-	2.48539	2813665:2813773	2813726	u
XC_2822	fumarate hydratase	-	3.25672	3393199:3393500	3393338	ui
	**Signal Transduction**					
XC_3141	AraC family transcriptional regulator	-	4.0823	3754979:3755270	3755080	u
XC_2486	galactose-binding protein regulator	-	3.08531	3017020:3017202	3017106	u
XC_0562	glutamine synthetase	+	4.66677	671351:672679	672562	ui
XC_0522	PbsX family transcriptional regulator	-	3.54119	619434:619888	619735	u
XC_0367	PobR regulator	-	2.87838	428274:428410	428337	u
XC_1153	chromosome replication initiation inhibitor protein	-	3.09086	1395486:1395903	1395675	u
XC_2652	histidine utilization repressor	-	3.08531	3191052:3191324	3191186	u
XC_0506	LysR family transcriptional regulator	-	2.98787	596106:596545	596455	u
XC_2840	MarR family transcriptional regulator	-	3.51383	3415840:3416615	3416086	u
XC_4314	sugar diacide regulator	-	3.29885	5121398:5122943	5121523	ui
XC_0246	transcriptional regulator	-	3.27655	297036:297238	297119	u
XC_4261	transcriptional regulator	-	2.48427	5056153:5056446	5056250	u
XC_0987	sensor histidine kinase	-	3.81482	1183931:1184389	1184094	u
XC_1474	RNA polymerase ECF-type sigma factor	-	2.6568	1773738:1773874	1773837	u
XC_1193	RNA polymerase sigma-70 factor	-	2.39969	1450328:1450586	1450541	u
XC_0114	two-component system regulatory protein	+	3.75402	133399:134337	133703	ui
XC_0496	two-component system regulatory protein	-	3.13862	587146:587443	587345	u
XC_3055	two-component system regulatory protein	-	2.4518	3656912:3657299	3657022	u
XC_3997	two-component system regulatory protein	-	3.1008	4712960:4713751	4713096	ui
XC_1939	two-component system sensor protein	-	2.43666	2343598:2343795	2343740	u
XC_3125	two-component system sensor protein	-	15.25212	3732825:3734906	3732989	ui
XC_3451	two-component system sensor protein	-	6.42023	4099702:4100744	4100377	i
XC_3529	two-component system sensor protein	-	3.60057	4191855:4192033	4191934	u

	**Transcription and Translation**					
XC_3943	bifunctional phosphopantothenoylcysteine decarboxylase/phosphopantothenate synthase	-	3.49932	4657069:4657268	4657181	u
XC_2667	chromosome segregation protein	-	3.34242	3215725:3216588	3215790	u
XC_0520	Fis family transcriptional regulator	-	4.56886	617324:617805	617696	u
XC_2343	single stranded DNA exonuclease	-	2.5711	2834013:2834492	2834214	ui
XC_1464	6-O-methylguanine-DNA methyltransferase	-	3.59953	1766102:1766578	1766194	u
XC_2951	ATP-dependent DNA ligase	+	4.19946	3535812:3536495	3536101	di
XC_4189	recombination associated protein	-	2.65038	4945666:4945832	4945748	u
XC_4123	50S ribosomal protein L28	-	2.49873	4858412:4858649	4858496	u
XC_4178	glycyl-tRNA synthetase subunit alpha	-	2.21892	4934235:4934454	4934331	u
XC_2663	lysyl-tRNA synthetase	-	3.02482	3207540:3207719	3207617	u
XC_3370	ribonuclease BN/unknown domain fusion protein	-	3.38107	4021031:4021191	4021097	u
XC_3256	ribonuclease H	-	2.74769	3898772:3898938	3898856	u
XC_3709	ribosomal small subunit pseudouridylate synthase	-	2.32399	4397791:4397938	4397844	u
XC_3592	ribosomal-protein-alanine acetyltransferase	-	3.99238	4272971:4273687	4273354	ui
XC_2383	histidyl-tRNA synthetase	-	2.7425	2875830:2876058	2875977	u
XC_0219	tRNA/rRNA methyltransferase	-	2.99299	266618:266863	266781	u
XC_1366	Holliday junction resolvase-like protein	+	3.34242	1650827:1651485	1651235	ui
XC_4360	tRNA Ser	-	2.6568	3177120:3177368	3177199	u
XC_4364	tRNA-Thr	-	2.32859	3695489:3695606	3695541	u
	**Other Macromolecules**					
XC_0505	2-acylglycerophosphoethanolamine acyltransferase	-	3.83105	594965:595391	595026	ui
XC_3360	molecular chaperone LolB	-	2.53691	4009852:4010222	4010140	u
XC_0652	outer membrane lipoprotein	-	2.84509	786729:786942	786788	u
XC_2910	cyclopropane-fatty-acyl-phospholipid synthase	+	2.70682	3495190:3495506	3495260	ui

XC_3594	phosphatidylserine synthase	-	3.94235	4274499:4274883	4274588	u
XC_0424	4-alpha-glucanotransferase	+	4.6261	499315:500467	499444	ui
XC_2173	exopolysaccharide biosynthesis protein	+	3.42813	2617995:2618330	2618247	ui
	**Protein Maintenance and Folding**					
XC_0765	disulfide oxidoreductase	-	3.44979	920642:921148	920724	di
XC_3086	lipoprotein signal peptidase	-	2.64896	3697165:3697299	3697256	u
XC_0077	metalloprotease	-	4.62589	91583:92184	91707	ui
XC_3653	oligopeptidase A	-	3.75504	4336999:4337250	4337111	u
XC_1249	prfC peptide chain release factor 3	-	3.94235	1529958:1530248	1530117	u
XC_3198	curved DNA binding protein	-	2.22828	3833728:3833931	3833894	u
XC_1721	heat shock protein 90	-	3.77094	2082215:2082677	2082477	u
XC_0253	dipeptidyl anminopeptidase	-	2.14475	308082:308191	308123	u
XC_3394	proline iminopeptidase chain A	-	2.37866	4046599:4046757	4046680	u
XC_0714	tail-specific protease	+	6.78533	856541:857309	856870	ui
XC_4317	dipeptidase	-	2.27506	5126321:512655	5126462	u
XC_3378	extracellular protease	-	2.31047	4028571:4028730	4028644	u
XC_0237	peptidyl-tRNA hydrolase domain-containing protein	+	2.89594	285192:285796	285367	ui
XC_3431	hydrolase	-	3.92703	4080892:4081125	4080977	u
	**Pathogenicity, Virulence, and Adaptation**					
XC_1013	sulfur deprivation response regulator	-	2.45885	1226905:1227023	1226929	u
XC_2461	glucan 1,4-beta-glucosidase	-	3.17102	2979412:2979556	2979493	u
XC_0639	cellulase	-	37.89331	767442:768475	768312	ui
XC_3004	HrpB8 protein	-	3.75144	3600676:3600904	3600821	u
XC_3006	type III secretion system ATPase	-	4.19946	3602206:3602944	3602810	ui
XC_3007	type III secretion system protein HrpB	-	4.28516	3603117:3603871	3603765	u
XC_3563	general secretion pathway protein D	-	4.62126	4232320:4232853	4232450	ui

XC_3569	general secretion pathway protein I	-	6.23889	4237109:4237475	4237373	u
XC_3564	general secretion pathway protein N	-	4.62126	4232320:4232853	4232450	di
XC_0740	type II secretion system protein E	+	5.45234	887912:888306	888053	ui
XC_2031	Type IV secretory pathway, VirB4 components	-	3.17102	2448405:2448538	2448475	u
XC_0152	carboxylesterase type B	-	3.30607	191674:192008	191935	ui
XC_4126	cation efflux system protein	-	4.20065	4864586:4865672	4865302	u
XC_3283	colicin V production protein	-	2.88076	3931966:3932490	3932297	u
XC_2807	glutathione S-transferase	-	3.471	3379172:3379658	3379462	u
XC_3833	multidrug efflux protein	-	3.8656	4531065:4532968	4532441	u
XC_2838	multidrug resistance efflux pump	-	2.14258	3413805:3413973	3413897	u
XC_0042	NonF-like protein	-	2.24805	53064:53229	53111	u
XC_1181	TonB protein	-	2.7425	1433328:1433476	1433425	u
XC_4127	cation efflux system protein	-	4.20065	4864586:4865672	4865302	i
XC_3656	copper resistance protein A	+	4.25257	4338272:4339122	4338701	ui
	**Hypothetical Protein**					
XC_0785		-	2.30203	943068:943175	943098	u
XC_4241	phytochrome-like protein	-	3.21581	5029233:5029432	5029320	u
XC_1699	sarcosine oxidase alpha subunit	+	3.08531	2039158:2039540	2039445	ui
XC_3817	Sun protein	-	3.62178	4513456:4513944	4513870	ui
XC_4305	alginate lyase	-	3.19423	5108042:5108682	5108392	u
XC_0566	hypothetical protein	-	3.89093	679660:680412	680278	u
XC_0033	hypothetical protein	-	2.28719	44784:44911	44826	u
XC_0053	hypothetical protein	-	2.86978	68827:69024	68905	u
XC_0074	hypothetical protein	-	2.80961	88476:89062	88561	ui
XC_0088	hypothetical protein	-	4.21637	103446:103931	103826	u
XC_0089	hypothetical protein	-	3.82322	104654:107292	105822	ui
XC_0091	hypothetical protein	-	4.01616	107438:108349	107956	ui
XC_0117	hypothetical protein	-	2.28527	138673:138806	138773	u

XC_0164	hypothetical protein	-	3.29544	205171:205435	205269	i
XC_0168	hypothetical protein	-	2.25365	211527:211657	211583	u
XC_0189	hypothetical protein	+	3.48507	234116:234475	234383	ui
XC_0236	hypothetical protein	-	2.89594	285192:285796	285367	ui
XC_0244	hypothetical protein	-	4.76878	295082:295801	295570	ui
XC_0392	hypothetical protein	+	3.56312	454190:455202	454598	ui
XC_0454	hypothetical protein	-	3.22916	540933:541320	541038	ui
XC_0497	hypothetical protein	-	2.6296	587786:588059	587897	u
XC_0508	hypothetical protein	-	3.1845	599802:600190	599890	u
XC_0514	hypothetical protein	+	4.70401	606427:607371	607024	ui
XC_0528	hypothetical protein	-	2.90259	625192:625321	625249	u
XC_0561	hypothetical protein	+	4.66677	671351:672679	672562	ui
XC_0571	hypothetical protein	-	3.13779	684055:684298	684110	u
XC_0583	hypothetical protein	-	2.81243	696101:696204	696155	u
XC_0605	hypothetical protein	-	2.4361	723445:723839	723780	u
XC_0606	hypothetical protein	-	3.38798	723969:724375	724071	ui
XC_0612	hypothetical protein	-	2.8959	730526:730700	730574	u
XC_0619	hypothetical protein	-	2.27334	737474:737887	737514	u
XC_0653	hypothetical protein	+	4.05234	787079:787670	787573	ui
XC_0715	hypothetical protein	-	5.4962	860226:860498	860342	u
XC_0782	hypothetical protein	-	3.28637	940107:940269	940201	u
XC_0791	hypothetical protein	-	3.40344	946431:946676	946589	ui
XC_0798	hypothetical protein	-	3.27293	954096:954325	954239	u
XC_0817	hypothetical protein	-	2.90238	976645:977144	976685	u
XC_0825	hypothetical protein	+	2.67447	990102:990505	990338	ui
XC_0877	hypothetical protein	-	3.51915	1058439:1058668	1058538	u
XC_0883	hypothetical protein	-	2.5589	1064372:1064865	1064426	ui
XC_0900	hypothetical protein	-	5.27321	1084452:1085088	1084879	u
XC_0902	hypothetical protein	-	2.73542	1085963:1086161	1086105	u

XC_0931	hypothetical protein	-	5.66923	1117616:1118064	1117804	i
XC_0933	hypothetical protein	-	3.63761	1122070:1122380	1122274	u
XC_0934	hypothetical protein	-	3.79788	1122561:1123329	1122963	u
XC_0935	hypothetical protein	-	4.32961	1123520:1123699	1123608	u
XC_1052	hypothetical protein	-	2.91391	1268562:1268757	1268704	u
XC_1072	hypothetical protein	+	3.85664	1288465:1289544	1288948	ui
XC_1100	hypothetical protein	+	3.33587	1327060:1328148	1328052	ui
XC_1106	hypothetical protein	-	3.02885	1335799:1335933	1335847	u
XC_1250	hypothetical protein	-	2.36445	1531044:1531357	1531306	u
XC_1485	hypothetical protein	-	2.80262	1791994:1792394	1792241	u
XC_1488	hypothetical protein	-	3.24975	1793873:1794103	1793964	u
XC_1826	hypothetical protein	-	2.31399	2220762:2220956	2220938	u
XC_1906	hypothetical protein	-	3.34242	2302964:2303080	2303014	u
XC_2382	hypothetical protein	-	2.22828	2874260:2874516	2874337	u
XC_2385	hypothetical protein	-	2.05688	2877507:2877654	2877564	u
XC_2481	hypothetical protein	-	3.08531	3005015:3005163	3005106	i
XC_2501	hypothetical protein	-	3.08531	3029743:3030026	3029948	u
XC_2696	hypothetical protein	-	3.02482	3243462:3244139	3243827	u
XC_2775	hypothetical protein	-	5.91352	3332997:3333259	3333127	u
XC_2805	hypothetical protein	-	2.64442	3376356:3376574	3376495	u
XC_2932	hypothetical protein	-	3.31218	3518455:3518769	3518604	u
XC_2950	hypothetical protein	+	2.89959	3533615:3534328	3534228	ui
XC_2960	hypothetical protein	-	4.37086	3546101:3546612	3546233	ui
XC_3044	hypothetical protein	-	3.73565	3643816:3644249	3643950	ui
XC_3127	hypothetical protein	+	15.25212	3732825:3734906	3732989	ui
XC_3149	hypothetical protein	-	4.19946	3767774:3768009	3767901	i
XC_3162	hypothetical protein	-	2.54359	3790452:3790588	3790538	u
XC_3166	hypothetical protein	-	3.40128	3794406:3794566	3794481	u
XC_3231	hypothetical protein	-	2.96652	3876685:3876891	3876812	u

XC_3234	hypothetical protein	-	2.15534	3879597:3879826	3879811	u
XC_3250	hypothetical protein	-	2.59725	3894046:3894187	3894116	u
XC_3284	hypothetical protein	-	3.24075	3933087:3933566	3933183	u
XC_3286	hypothetical protein	-	2.78729	3935851:3935971	3935931	u
XC_3403	hypothetical protein	-	2.03222	4055615:4055889	4055688	u
XC_3407	hypothetical protein	-	3.45724	4059145:4059405	4059243	u
XC_3446	hypothetical protein	-	2.60573	4096178:4096376	4096280	u
XC_3461	hypothetical protein	-	3.1053	4111540:4111837	4111748	ui
XC_3494	hypothetical protein	-	1.94702	4152457:4152567	4152512	u
XC_3498	hypothetical protein	-	2.25036	4155925:4156426	4156178	ui
XC_3522	hypothetical protein	-	4.8783	4183694:4184664	4184135	u
XC_3524	hypothetical protein	+	3.10525	4185122:4185422	4185377	ui
XC_3525	hypothetical protein	+	3.27819	4185558:4185954	4185854	ui
XC_3553	hypothetical protein	-	3.33736	4218311:4218902	4218681	ui
XC_3583	hypothetical protein	-	2.93452	4260696:4261029	4260744	ui
XC_3584	hypothetical protein	-	2.23904	4261991:4262202	4262048	u
XC_3692	hypothetical protein	-	3.74909	4380579:4380885	4380727	u
XC_3726	hypothetical protein	+	4.1898	4411988:4412310	4412097	ui
XC_3787	hypothetical protein	-	3.32901	4482293:4482745	4482361	ui
XC_3793	hypothetical protein	-	3.1008	4488709:4489349	4488802	u
XC_3811	hypothetical protein	+	3.49638	4504854:4505216	4505081	u
XC_3827	hypothetical protein	+	3.96963	4523317:4523966	4523479	ui
XC_3865	hypothetical protein	-	3.03905	4572094:4572495	4572285	u
XC_3878	hypothetical protein	+	3.38925	4581457:4582139	4582042	ui
XC_3897	hypothetical protein	-	3.79497	4604153:4604462	4604321	u
XC_4024	hypothetical protein	-	4.00259	4738848:4739946	4739823	ui
XC_4039	hypothetical protein	-	3.00527	4755711:4756082	4755790	u
XC_4091	hypothetical protein	-	2.15225	4821990:4822222	4822010	u
XC_4111	hypothetical protein	-	3.1251	4843713:4844358	4844291	u

XC_4113	hypothetical protein	-	2.35746	4845331:4845441	4845395	u
XC_4124	hypothetical protein	-	2.77808	4859348:4859723	4859448	u
XC_4172	hypothetical protein	-	2.82742	4923829:4924108	4924012	u
XC_4251	hypothetical protein	-	2.82683	5045065:5045354	5045118	u
XC_4264	hypothetical protein	-	3.08381	5059045:5059560	5059287	u
XC_4266	hypothetical protein	+	3.08381	5059045:5059560	5059287	ui
XC_4304	hypothetical protein	-	2.51094	5106258:5106629	5106558	u

[a] Names and codes of identified genes are according to genomic annotation of *X. campestris* pv. *campestris* 8004.
