# Supplementary material for: Two-Component Signaling System VgrRS Directly Senses Extracytoplasmic and Intracellular Iron to Control Bacterial Adaptation under Iron Depleted Stress
Source: PLoS Pathog. 2016 Dec 30;12(12):e1006133. doi: 10.1371/journal.ppat.1006133 (PMC5231390; doi:10.1371/journal.ppat.1006133)
Supplement: S7 Table — (PDF) [file ppat.1006133.s013.pdf]

S7 Table. ChIP-seq analysis identifies genes with promoter regions bound by VgrR in *X. campestris*. pv. *campestris* grown in MMX supplemented with 100 µM Fe^{3+} medium

Code[a]	Gene function	Strand	Seq. peak Z-score	Peak start:peak end	Peak position	Direction
	**Amino Acid Metabolism**					
XC_2854	2,3,4,5-tetrahydropyridine-2,6-carboxylate N-succinyltransferase	-	2.55441	3433767:3433967	3433922	u
XC_0100	aromatic amino acid aminotransferase	-	2.53656	119207:119678	119264	u
XC_2806	asparagine synthase	-	2.59845	3377543:3377693	3377610	i
XC_0394	aspartyl/asparaginyl beta-hydroxylase	-	2.85514	457177:457603	457533	u
XC_0982	cysteine synthase	-	2.53866	1179280:1179489	1179349	u
XC_3655	cysteine synthase	-	3.90456	4338333:4339128	4338998	u
XC_0484	indole-3-glycerol phosphate synthase	+	3.38318	573490:573950	573551	ui
XC_4041	shikimate 5-dehydrogenase aroE	-	4.21687	4755651:4756703	4756408	di
XC_0293	serine-pyruvate aminotransferase	-	3.40328	348624:349321	348909	u
XC_0623	cyclohexadienyl dehydratase	-	2.90618	740555:741075	740705	ui
	**Biosynthesis of Small Molecules**					
XC_1225	adenosylmethionine-8-amino-7-oxononanoate aminotransferase	-	2.04368	1499407:1499629	1499468	i
XC_3088	bifunctional riboflavin kinase/FMN adenylyltransferase	-	1.9696	3701337:3701670	3701402	u
XC_0400	biotin synthase	-	3.21032	463537:464186	463952	u
XC_0395	biotin synthesis protein	-	2.56919	458376:458997	458463	u
XC_2553	biotin synthesis protein	-	2.73058	3088500:3088802	3088573	u
XC_1101	cobalamin synthase	+	2.55441	1327660:1328172	1328036	ui
XC_4108	coproporphyrinogen III oxidase	+	3.04467	4838458:4838922	4838855	ui
XC_3887	dihydroneopterin aldolase	-	2.05985	4592730:4593180	4592810	u

XC_0301	gamma-glutamyltranspeptidase	-	3.31985	357732:358528	357980	ui
XC_0796	glutaredoxin-like protein	-	2.40296	951344:951709	951625	i
XC_0575	malonate decarboxylase subunit gamma	+	2.98607	687951:688408	688008	di
XC_0206	porphyrin biosynthesis protein	-	2.51323	255574:255909	255741	i
XC_0733	pteridine reductase	-	2.14796	878285:878510	878391	u
XC_1169	pyrroloquinoline quinone biosynthesis protein PqqE	-	2.49686	1416286:1416411	1416355	u
XC_2468	3-methyl-2-oxobutanoate hydroxymethyltransferase	-	2.51037	2986231:2986511	2986269	u
XC_4025	pantothenate kinase	-	3.07628	4738841:4739957	4739687	di
	**Fatty acid Metabolism**					
XC_1821	acetoacetyl-CoA reductase	-	2.50927	2214857:2214998	2214913	u
XC_0578	ACP S-malonyltransferase	+	2.61194	689376:690212	689567	ui
XC_4101	acyl carrier protein	-	1.86867	4829659:4829758	4829715	u
XC_0516	CDP-diacylglycerol--glycerol-3-phosphate 3-phosphatidyltransferase	+	3.72638	608607:610206	608966	ui
XC_4096	fatty acyl CoA synthetase	-	1.92599	4826631:4826862	4826649	u
XC_0254	biotin carboxylase	-	2.88556	308348:308994	308536	i
XC_4117	cardiolipin synthase	-	3.96869	4852176:4853510	4853307	ui
	**Nucleotide Metabolism**					
XC_0510	bifunctional phosphoribosylaminoimidazolecarboxamide formyltransferase/IMP cyclohydrolase	-	3.6758	603672:604160	604060	u
XC_0670	cytosine deaminase	-	2.6846	804469:804700	804586	u
XC_2957	hypoxanthine-guanine phosphoribosyltransferase	-	2.15804	3542293:3542370	3542334	u
XC_0553	gluconolactonase	-	3.8171	664053:664583	664145	ui
XC_2686	transferase	-	2.24612	3232785:3232890	3232844	u

	**Cell Structures and Division**					
XC_3449	diacylglycerol kinase	-	1.89439	4098516:4098655	4098637	u
XC_3941	phosphomannomutase	-	2.58744	4654201:4654540	4654275	ui
XC_0695	rod shape-determining protein	+	2.19958	835038:835521	835455	ui
XC_0504	transmembrane protein	-	1.97806	592831:593187	592861	u
XC_4184	twin-arginine translocation protein TatA	-	3.83786	4940351:4940808	4940419	u
XC_3508	D-alanine--D-alanine ligase ddl	-	1.90284	4168540:4169114	4168589	u
XC_0232	D-alanyl-alanine synthetase A	-	2.7463	280714:281461	280952	ui
XC_3877	N-acetylmuramoyl-L-alanine amidase	-	2.79042	4581487:4582158	4581631	ui
XC_3510	undecaprenyldiphospho-muramoylpentapeptide beta-N-acetylglucosaminyltransferase MurG	-	2.7159	4170244:4171355	4170946	ui
XC_0517	acyltransferase	+	3.72638	608607:610206	608966	ui
XC_4168	NdvB protein	-	2.2424	4918834:4918962	4918898	u
XC_3504	UDP-3-O-[3-hydroxymyristoyl] N-acetylglucosamine deacetylase lpxC	-	2.52212	4163661:4164041	4163723	u
XC_3506	cell division protein	-	2.29016	4166690:4166820	4166725	u
XC_3507	cell division protein	-	1.98187	4167529:4167633	4167612	u
XC_0843	chemotaxis protein	+	2.59661	1015481:1015860	1015548	ui
	**Transport Protein**					
XC_3847	ABC transporter ATP-binding protein	-	3.04833	4545019:4545485	4545118	u
XC_3745	amino acid transporter	-	2.6745	4436047:4436224	4436142	u
XC_2844	bacterioferritin	-	2.11399	3421151:3421258	3421213	u
XC_3785	ferrichrome-iron receptor 3	-	2.14628	4479916:4480052	4480011	u
XC_4249	ferrichrome-iron receptor 3	-	3.6819	5038869:5039709	5039547	u
XC_0167	ferripyoverdine receptor	+	3.51518	207517:207966	207822	ui
XC_2355	ferrous iron transport protein	-	2.02591	2846926:2847014	2846956	u

XC_0952	glucarate transporter	-	1.67796	1145193:1145308	1145293	u
XC_0371	glycerol uptake facilitator protein	-	2.65196	432780:433235	433121	u
XC_1104	iron transporter	-	2.31042	1332682:1333344	1333041	ui
XC_0570	iron utilization protein	-	3.61842	683229:683597	683492	ui
XC_0218	MFS transporter	+	2.41272	265603:266036	265652	ui
XC_0460	monovalent cation/H+ antiporter subunit A	-	2.66748	547807:548044	547949	u
XC_0459	monovalent cation/H+ antiporter subunit C	-	2.52541	545052:545627	545370	u
XC_0457	monovalent cation/H+ antiporter subunit E	-	1.73009	542979:543421	543306	u
XC_4257	outer membrane efflux protein	+	2.66338	5048568:5049110	5048745	ui
XC_0814	periplasmic iron-binding protein	+	2.93413	973791:974197	974131	ui
XC_0687	TonB-dependent receptor	+	1.77926	822044:822459	822222	ui
XC_2296	TonB-dependent receptor	-	1.93783	2768717:2768794	2768748	u
XC_4141	TonB-dependent receptor	-	2.97302	4882526:4883171	4882962	u
XC_1241	TonB-dependent receptor	+	1.25854	1518223:1518502	1518274	u
XC_3990	transporter	+	2.97418	4704343:4704715	4704408	ui
XC_4182	sec-independent protein translocase	-	2.11982	4939517:4939650	4939573	u
	**Central Intermediary**					
XC_3359	4-diphosphocytidyl-2-C-methyl-D-erythritol kinase	-	2.92933	4009104:4009402	4009192	u
XC_3537	6,7-dimethyl-8-ribityllumazine synthase ribH	-	2.28827	4201309:4201709	4201675	u
XC_3830	tropinone reductase	-	2.70452	4527432:4527568	4527499	u
XC_1334	sulfite reductase	-	1.94684	1621887:1622186	1621925	u
	**Degradive Enzymes**					
XC_0381	3-carboxy-cis,cis-muconate cycloisomerase	+	3.77236	441972:442800	442521	ui
XC_4227	acid phosphatase	-	1.95321	5003164:5003478	5003413	ui

XC_0822	alpha-1,2-mannosidase	-	1.94477	988767:988942	988908	u
XC_3886	beta-glucosidase	-	2.49685	4592086:4592344	4592132	u
XC_0378	beta-ketoadipyl CoA thiolase	+	2.24662	439406:439945	439680	ui
XC_3749	D-amino acid oxidase	+	2.5378	4438506:4439258	4438816	ui
XC_2978	enoyl-CoA hydratase	-	2.46633	3565924:3566115	3566052	u
XC_4315	glycerate kinase	-	2.10949	5123326:5123524	5123472	u
XC_0150	L-fucose dehydrogenase	+	2.61187	187658:188180	188033	ui
XC_0683	MoxJ protein	+	2.18077	817383:817888	817805	ui
XC_0374	phenoxybenzoate dioxygenase subunit beta	-	1.95128	436723:436940	436863	u
XC_4154	xylosidase	-	2.63087	4898287:4898737	4898539	u
XC_4214	xylosidase/arabinosidase	+	2.51154	4982083:4982394	4982260	ui
XC_2477	xylose isomerase	-	1.71762	2996557:2996628	2996617	u
	**Energy Metabolism**					
XC_1743	1-phosphofructokinase	-	2.06995	2110224:2110313	2110261	u
XC_0686	alcohol dehydrogenase	+	2.14112	820635:821175	820786	ui
XC_3684	ATP synthase F0F1 subunit A	-	2.20083	4371755:4371869	4371821	u
XC_0441	branched-chain alpha-keto acid dehydrogenase subunit E2	-	1.82972	525054:525371	525104	u
XC_1885	cytochrome D ubiquinol oxidase subunit I	-	2.73058	2281639:2281772	2281691	u
XC_3691	dihydrolipoamide acetyltransferase	-	2.66059	4379732:4379977	4379908	u
XC_3689	dihydrolipoamide dehydrogenase	-	2.69622	4377335:4377856	4377756	u
XC_4120	FldA protein	+	2.9759	4853713:4854964	4854846	ui
XC_3287	phosphoglycerate mutase	-	2.67651	3936411:3936556	3936460	u
XC_3896	protoheme IX farnesyltransferase	-	2.4293	4602843:4603342	4602933	ui
XC_0922	reductase	-	1.61274	1107002:1107181	1107142	u
XC_1700	oxidoreductase	+	2.29016	2039385:2040158	2040097	ui

XC_3948	oxidoreductase	+	2.57611	4660659:4661360	4661115	ui
XC_3965	oxidoreductase	-	3.38208	4682365:4682719	4682433	di
XC_4157	oxidoreductase	+	3.30324	4900074:4900518	4900333	ui
XC_3431	hydrolase	-	2.71646	4080868:4081147	4080988	u
XC_3708	hydrolase	-	2.42843	4397011:4397445	4397377	ui
XC_0289	monoxygenase	-	2.52185	345702:345954	345765	u
	**Signal Transduction**					
XC_3141	AraC family transcriptional regulator	-	2.57896	3754989:3755203	3755082	u
XC_4163	AraC family transcriptional regulator	+	1.88624	4907366:4907726	4907514	ui
XC_3852	bifunctional isocitrate dehydrogenase kinase/phosphatase protein aceK	-	2.10617	4552197:4552505	4552261	ui
XC_3853	B-lactamase regulatory protein	-	2.10617	4553341:4553463	4553433	u
XC_3829	diguanylate cyclase GGDEF family protein	-	1.78662	4526743:4526813	4526749	u
XC_2486	galactose-binding protein	-	2.64249	3017081:3017203	3017148	u
XC_0562	glutamine synthetase	+	3.43338	671321:672950	671461	ui
XC_3929	signal transducer	-	1.86811	4644338:4644427	4644369	u
XC_0522	PbsX family transcriptional regulator	-	2.79301	619473:619841	619709	u
XC_0367	PobR regulator	-	2.44102	428279:428492	428382	u
XC_0312	LysR family transcriptional regulator	-	2.22596	370711:371217	371132	u
XC_0506	LysR family transcriptional regulator	-	3.40993	596022:596876	596197	u
XC_2840	MarR family transcriptional regulator	-	2.14432	3415838:3415930	3415873	u
XC_4192	sal operon transcriptional repressor	-	2.32355	4949453:4950056	4949649	ui
XC_0072	transcriptional regulator	-	2.91342	86200:86583	86358	ui
XC_0246	transcriptional regulator	-	2.63052	296989:297540	297202	u
XC_3386	transcriptional regulator	-	2.4586	4036327:4036518	4036475	u
XC_4118	transcriptional regulator	-	2.9759	4853713:4854964	4854846	u

XC_4261	transcriptional regulator	-	3.1846	5056142:5056513	5056354	u
XC_0987	sensor histidine kinase	-	3.49549	1183950:1184405	1184238	u
XC_0114	two-component system regulatory protein	+	3.85235	133599:134702	133676	ui
XC_3055	two-component system regulatory protein	-	2.09336	3656960:3657345	3657013	u
XC_3117	two-component system regulatory protein	-	1.90306	3727744:3727866	3727805	u
XC_3126	two-component system regulatory protein	-	2.44581	3734153:3734320	3734275	u
XC_3997	two-component system regulatory protein	-	3.23127	4712924:4713810	4713476	ui
XC_3529	two-component system sensor protein	-	2.92755	4191857:4192118	4191943	u
XC_3998	two-component system sensor protein	-	1.68687	4714346:4714543	4714357	u
XC_3845	response regulator protein	-	2.85883	4543408:4544086	4543902	u
	**Transcription and Translation**					
XC_4038	ATP-dependent DNA helicase DinG	-	2.81713	4754318:4755514	4755163	ui
XC_0947	ATP-dependent DNA helicase RecG	-	2.52009	1138965:1139078	1139024	u
XC_3943	bifunctional phosphopantothenoylcysteine decarboxylase/phosphopantothenate synthase	-	2.89632	4657069:4657301	4657132	u
XC_2667	chromosome segregation protein	-	3.3912	3215746:3216026	3215835	u
XC_3148	DNA polymerase III subunit alpha	-	2.28391	3766780:3766868	3766815	u
XC_3112	DNA-3-methyladenine glycosylase	-	1.85849	3724773:3724908	3724901	u
XC_0520	Fis family transcriptional regulator	-	3.85993	617332:617842	617681	u
XC_4203	histone	-	2.02266	4964451:4964554	4964499	u
XC_1464	6-O-methylguanine-DNA methyltransferas	-	2.20208	1766129:1766622	1766157	u
XC_4123	50S ribosomal protein L28 rpmB	-	1.698	4858439:4858597	4858488	u
XC_4178	glycyl-tRNA synthetase subunit alpha glyQ	-	2.35722	4934191:4934833	4934577	u
XC_2663	lysyl-tRNA synthetase	-	1.61983	3207554:3207655	3207595	u
XC_3370	ribonuclease BN/unknown domain fusion protein	-	1.94575	4021044:4021171	4021146	u

XC_3256	ribonuclease H	-	1.86281	3898817:3898913	3898854	u
XC_3709	ribosomal small subunit pseudouridylate synthase	-	2.50504	4397802:4397959	4397872	u
XC_3592	ribosomal-protein-alanine acetyltransferase	-	4.36154	4272987:4273693	4273285	ui
XC_0219	tRNA/rRNA methyltransferase	-	3.25418	266622:267015	266798	u
XC_1224	16S ribosomal RNA methyltransferase RsmE	-	2.04368	1499407:1499629	1499468	u
XC_3807	D-tyrosyl-tRNA(Tyr) deacylase	-	2.03227	4502111:4502423	4502152	u
XC_1366	Holliday junction resolvase-like protein	+	2.29016	1650855:1651182	1650908	ui
	**Other Macromolecules**					
XC_0505	2-acylglycerophosphoethanolamine acyltransferase	-	3.16142	594961:595904	595027	ui
XC_3360	molecular chaperone LolB	-	2.06182	4009864:4010187	4009924	u
XC_0652	outer membrane lipoprotein	-	2.56544	786719:786985	786808	u
XC_3594	phosphatidylserine synthase	-	3.73264	4274445:4274857	4274570	u
	**Protein Maintenance and Folding**					
XC_0021	carboxyl-terminal protease	-	2.06945	25774:25859	25829	u
XC_0611	dipeptidyl peptidase IV	-	3.06078	728746:729687	729077	ui
XC_0765	disulfide oxidoreductase	-	4.28651	920661:921110	920824	d
XC_3086	lipoprotein signal peptidase	-	2.04052	3697158:3697292	3697226	u
XC_0077	metalloprotease	-	3.37818	91588:92557	91646	ui
XC_3653	oligopeptidase A	-	2.68484	4337031:4337366	4337144	u
XC_1249	peptide chain release factor 3	-	3.34716	1529935:1530218	1530109	u
XC_3550	serine protease	+	2.28541	4212641:4213003	4212698	ui
	**Pathogenicity, Virulence, and Adaptation**					
XC_1013	sulfur deprivation response regulator	-	1.78614	1226890:1227063	1226925	u

XC_0126	pectate lyase E	+	2.5256	153723:154126	153947	ui
XC_0639	cellulase	-	3.91447	768302:768408	768344	u
XC_3004	HrpB8 protein	-	2.00343	3600718:3600895	3600831	u
XC_3006	type III secretion system ATPase	-	2.64249	3602490:3602882	3602743	u
XC_3563	general secretion pathway protein D	-	3.65545	4232351:4232887	4232795	ui
XC_3569	general secretion pathway protein I	-	3.91259	4237238:4237480	4237367	u
XC_0739	type II secretion system protein D	+	2.94963	885232:886108	885673	ui
XC_0740	type II secretion system protein E	+	3.7757	887857:888222	888025	ui
XC_0741	type II secretion system protein F	+	3.33946	889160:889687	889471	ui
XC_4286	virulence associated protein	-	2.34474	5079509:5079704	5079636	u
XC_1069	competence lipoprotein	-	1.89379	1287658:1287856	1287816	u
XC_0152	carboxylesterase type B	-	3.05679	191665:192049	191829	ui
XC_3993	catalase	+	2.54129	4707833:4708828	4707973	ui
XC_4126	cation efflux system protein	-	3.16541	4864611:4865717	4865238	u
XC_3283	colicin V production protein	-	2.42588	3931961:3932446	3932120	u
XC_0671	ElaA protein	-	3.84608	804867:805565	805390	ui
XC_0672	multidrug resistance efflux pump	-	2.40412	806108:806229	806162	u
XC_0042	NonF-like protein	-	1.77339	53068:53198	53086	u
XC_3809	phosphinothricin acetyltransferase	-	3.16541	4503789:4504168	4504013	u
XC_0828	TolC protein	-	1.84752	996563:996736	996681	u
XC_1181	TonB protein	-	2.77462	1433369:1433494	1433435	u
XC_0907	beta-lactamase	-	3.51171	1090587:1090741	1090675	u
XC_3656	copper resistance protein A	+	3.90456	4338333:4339128	4338998	ui
	**Hypothetical Protein**					
XC_3817	Sun protein	-	2.63497	4512488:4513917	4513133	ui

XC_4305	alginate lyase	-	3.01323	5107994:5108954	5108391	u
XC_0615		-	2.03093	734148:734309	734274	u
XC_0785		-	2.15824	943066:943241	943152	u
XC_0030	hypothetical protein	-	2.74439	37975:38596	38038	u
XC_0033	hypothetical protein	-	2.74794	44720:44936	44838	u
XC_0048	hypothetical protein	+	2.51819	58323:58679	58582	ui
XC_0053	hypothetical protein	-	3.09554	67890:69213	68032	ui
XC_0078	hypothetical protein	-	3.05537	92694:92939	92763	u
XC_0088	hypothetical protein	-	3.44436	103507:104392	103890	u
XC_0089	hypothetical protein	-	4.24829	104603:108393	107597	ui
XC_0099	hypothetical protein	-	1.85169	117989:118179	118054	u
XC_0106	hypothetical protein	-	2.12819	125620:125807	125669	u
XC_0115	hypothetical protein	-	3.24262	136363:136970	136439	ui
XC_0116	hypothetical protein	+	3.24262	136363:136970	136439	ui
XC_0117	hypothetical protein	-	2.42392	138655:139236	139028	u
XC_0140	hypothetical protein	-	2.21403	174874:174959	174902	u
XC_0147	hypothetical protein	+	2.41821	185098:185514	185194	ui
XC_0164	hypothetical protein	-	2.78843	205882:206059	206022	u
XC_0168	hypothetical protein	-	2.15989	211472:211628	211521	u
XC_0169	hypothetical protein	-	3.00467	212485:213106	212628	ui
XC_0172	hypothetical protein	+	2.83758	214852:215315	214931	ui
XC_0199	hypothetical protein	+	1.52183	248800:249053	248820	u
XC_0211	hypothetical protein	+	2.04153	259505:259806	259597	ui
XC_0236	hypothetical protein	-	1.95128	285550:285800	285700	u
XC_0244	hypothetical protein	-	3.29692	295113:295839	295655	u
XC_0260	hypothetical protein	-	2.33719	315489:315841	315542	u

XC_0261	hypothetical protein	-	2.27876	317093:317326	317146	ui
XC_0288	hypothetical protein	-	2.33801	344333:344530	344425	u
XC_0332	hypothetical protein	-	2.66192	394020:394116	394050	u
XC_0351	hypothetical protein	-	2.46633	411495:411584	411548	u
XC_0358	hypothetical protein	-	1.92684	417966:418370	418080	u
XC_0359	hypothetical protein	-	1.50277	419037:419143	419037	u
XC_0392	hypothetical protein	+	3.06293	454131:455124	454499	ui
XC_0454	hypothetical protein	-	3.10665	540924:541410	541020	ui
XC_0462	hypothetical protein	-	1.88418	549430:549611	549499	u
XC_0470	hypothetical protein	-	1.92958	556912:557030	556965	u
XC_0497	hypothetical protein	-	2.56678	587886:588084	588021	u
XC_0508	hypothetical protein	-	2.10578	599755:600247	600147	ui
XC_0514	hypothetical protein	+	3.97504	606410:607328	606873	u
XC_0515	hypothetical protein	+	3.72638	608607:610206	608966	di
XC_0561	hypothetical protein	+	3.43338	671321:672950	671461	ui
XC_0566	hypothetical protein	-	3.37904	679601:680441	680221	u
XC_0571	hypothetical protein	-	3.60226	684037:684538	684102	u
XC_0583	hypothetical protein	-	2.41525	696119:696337	696156	u
XC_0591	hypothetical protein	-	1.9202	709119:709222	709164	u
XC_0604	hypothetical protein	-	2.81892	721497:722601	722326	ui
XC_0606	hypothetical protein	-	2.97155	723941:724423	724196	ui
XC_0612	hypothetical protein	-	2.79449	730028:730703	730604	ui
XC_0619	hypothetical protein	-	2.56544	737478:737913	737579	u
XC_0624	hypothetical protein	+	2.90618	740555:741075	740705	ui
XC_0653	hypothetical protein	+	3.94366	787110:787665	787592	ui
XC_0715	hypothetical protein	-	3.35541	860218:860486	860357	u

XC_0782	hypothetical protein	-	2.14919	940079:940295	940203	u
XC_0788	hypothetical protein	-	3.1064	944892:945404	945311	u
XC_0789	hypothetical protein	-	3.1064	944892:945404	94531	ui
XC_0791	hypothetical protein	-	2.64228	946486:946711	946573	u
XC_0795	hypothetical protein	-	2.40296	951344:951709	951625	u
XC_0798	hypothetical protein	-	2.07126	954040:954314	954080	ui
XC_0815	hypothetical protein	-	2.02325	975488:975637	975519	u
XC_0817	hypothetical protein	-	2.89005	976593:976942	976695	u
XC_0825	hypothetical protein	+	2.73862	989694:990559	990383	ui
XC_0883	hypothetical protein	-	1.91902	1064673:1064835	1064796	u
XC_0902	hypothetical protein	-	2.10397	1085965:1086089	1086027	u
XC_0931	hypothetical protein	-	1.98429	1119319:1119505	1119348	u
XC_0933	hypothetical protein	-	3.11289	1122133:1122341	1122221	u
XC_0934	hypothetical protein	-	2.80286	1122552:1123331	1123268	u
XC_0935	hypothetical protein	-	2.00412	1123486:1123698	1123649	u
XC_1052	hypothetical protein	-	2.59845	1268617:1268753	1268713	u
XC_1072	hypothetical protein	+	2.46633	1288500:1289595	1288613	ui
XC_1100	hypothetical protein	+	2.18808	1327214:1327523	1327439	ui
XC_1106	hypothetical protein	-	2.27579	1335765:1335909	1335803	u
XC_1338	hypothetical protein	-	2.06995	1625227:1625360	1625265	u
XC_1585	hypothetical protein	-	2.29016	1906117:1906215	1906168	u
XC_1612	hypothetical protein	-	2.06529	1936124:1936212	1936152	u
XC_1906	hypothetical protein	-	1.76166	2303015:2303086	2303029	u
XC_2290	hypothetical protein	-	2.81866	2758667:2759043	2758887	u
XC_2501	hypothetical protein	-	2.06995	3029746:3030000	3029921	u
XC_2696	hypothetical protein	-	2.14429	3243487:3243938	3243597	u

XC_2715	hypothetical protein	-	2.29016	3263122:3263229	3263176	u
XC_2775	hypothetical protein	-	2.8627	3333018:3333219	3333154	u
XC_2932	hypothetical protein	-	2.31517	3518483:3518774	3518634	u
XC_2960	hypothetical protein	-	2.95078	3546120:3546571	3546260	ui
XC_2989	hypothetical protein	-	2.3342	3583296:3583439	3583336	u
XC_3044	hypothetical protein	-	2.18677	3643802:3644247	3644025	ui
XC_3149	hypothetical protein	-	2.51037	3768422:3768968	768585	ui
XC_3166	hypothetical protein	-	2.32013	3794428:3794547	3794498	u
XC_3168	hypothetical protein	+	2.153	3795234:3795551	3795314	u
XC_3231	hypothetical protein	-	2.0326	3876653:3876890	3876721	u
XC_3238	hypothetical protein	+	2.13567	3882561:3882911	3882875	ui
XC_3284	hypothetical protein	-	2.06922	3933096:3933446	3933288	u
XC_3367	hypothetical protein	-	3.00295	4017872:4018538	4017951	i
XC_3403	hypothetical protein	-	2.10222	4055635:4055820	4055696	u
XC_3407	hypothetical protein	-	3.03314	4059189:4059395	4059339	u
XC_3446	hypothetical protein	-	2.5871	4096059:4096431	4096183	u
XC_3461	hypothetical protein	-	2.75762	4111545:4111894	4111832	u
XC_3522	hypothetical protein	-	3.79415	4183722:4184619	4184087	u
XC_3553	hypothetical protein	-	3.42944	4218263:4218841	4218534	ui
XC_3583	hypothetical protein	-	2.45431	4260921:4261017	4260960	u
XC_3584	hypothetical protein	-	2.45431	4260921:4261017	4260960	i
XC_3638	hypothetical protein	-	1.88475	4321607:4321857	4321818	u
XC_3692	hypothetical protein	-	3.20098	4380630:4380987	4380749	u
XC_3699	hypothetical protein	-	2.8952	4386309:4386841	4386535	ui
XC_3713	hypothetical protein	-	1.94994	4401134:4401222	4401157	u
XC_3716	hypothetical protein	-	1.92848	4404234:4404321	4404293	u

XC_3723	hypothetical protein	+	3.40561	4409334:4409864	4409738	ui
XC_3726	hypothetical protein	+	3.29719	4411927:4412294	4412159	u
XC_3755	hypothetical protein	+	2.33037	4443237:4443689	4443637	ui
XC_3793	hypothetical protein	-	3.07893	4488713:4489076	4488773	u
XC_3811	hypothetical protein	+	2.95481	4504818:4505322	4505087	ui
XC_3827	hypothetical protein	+	3.29761	4523269:4524356	4523854	ui
XC_3834	hypothetical protein	-	3.67471	4531264:4532998	4532415	ui
XC_3865	hypothetical protein	-	3.04505	4572039:4573162	4572747	ui
XC_3867	hypothetical protein	-	3.04505	4572039:4573162	4572747	di
XC_3878	hypothetical protein	+	2.79042	4581487:4582158	4581631	ui
XC_3894	hypothetical protein	+	2.97995	4598527:4599277	4598652	ui
XC_3897	hypothetical protein	-	2.78602	4604113:4604484	4604385	u
XC_3911	hypothetical protein	-	5.28056	4618205:4618688	4618475	ui
XC_4005	hypothetical protein	-	2.15262	4722980:4723390	4723252	u
XC_4024	hypothetical protein	-	3.07628	4738841:4739957	4739687	ui
XC_4039	hypothetical protein	-	4.21687	4755651:4756703	4756408	u
XC_4040	hypothetical protein	-	4.21687	4755651:4756703	4756408	ui
XC_4078	hypothetical protein	-	3.65002	4801258:4801739	4801652	i
XC_4086	hypothetical protein	-	3.12205	4811851:4815053	4813882	di
XC_4091	hypothetical protein	-	2.99515	4822010:4822252	4822126	u
XC_4095	hypothetical protein	-	2.72764	4825804:4826051	4825858	ui
XC_4111	hypothetical protein	-	2.78416	4843728:4844360	4844068	u
XC_4116	hypothetical protein	-	3.48894	4849286:4850124	4849777	i
XC_4124	hypothetical protein	-	2.34362	4859205:4859706	4859430	ui
XC_4128	hypothetical protein	-	2.13214	4866368:4866506	4866415	u
XC_4129	hypothetical protein	-	2.34802	4868414:4868830	4868757	u

XC_4156	hypothetical protein	+	3.30324	4900074:4900518	4900333	di
XC_4172	hypothetical protein	-	2.84661	4923808:4924291	4924060	ui
XC_4176	hypothetical protein	-	2.01095	4930791:4931027	4930831	u
XC_4229	hypothetical protein	-	2.48364	5005590:5005979	5005925	u
XC_4242	hypothetical protein	-	2.49184	5029765:5029970	5029819	u
XC_4251	hypothetical protein	-	2.16555	5045054:5045341	5045136	u
XC_4264	hypothetical protein	-	2.75174	5059039:5059545	5059225	u
XC_4266	hypothetical protein	+	2.75174	5059039:5059545	5059225	ui
XC_4304	hypothetical protein	-	2.85717	5105648:5106710	5106532	ui
XC_4312	hypothetical protein	-	1.80344	5118781:5118865	5118807	u
XC_4313	hypothetical protein	-	1.95471	5120606:5120712	5120646	u

[a] Names and codes of identified genes are according to genomic annotation of *X. campestris* pv. *campestris* 8004.
